# DFT Study on Fused *N*-Heteroaromatic Frameworks: Stability, Aromaticity, and Energetic Insights from Five-Membered Fused Six-Membered *N*-Heteroaromatic Skeletons

**DOI:** 10.3390/molecules30051101

**Published:** 2025-02-27

**Authors:** Zujia Lu, Cong Li, Shaoqun Li, Qiyao Yu, Jianguo Zhang

**Affiliations:** 1 State Key Laboratory of Explosion Science and Technology, Beijing Institute of Technology, Beijing 100081, China; 15201657211@163.com (Z.L.); l18656815056@163.com (C.L.); 3220235040@bit.edu.cn (S.L.); 2 State Key Laboratory of Transient Chemical Effects and Control, Shaanxi Applied Physics and Chemistry Research Institute, Xi’an 710061, China

**Keywords:** energetic skeleton, DFT, wave function analysis, molecule screening

## Abstract

The five-membered fused six-membered nitrogen heteroaromatic ring system is a crucial skeleton in the design and synthesis of energetic compounds. Based on this skeleton, many high-performance energetic compounds have been synthesized. However, to date, no one has conducted a systematic study on the characteristics of this skeleton itself. To assess how the number and position of nitrogen atoms affect the energy and stability of this type of skeleton, one to four nitrogen-substituted skeleton molecules were analyzed using Density Functional Theory (DFT) calculations. Natural population analysis (NPA), Laplacian bond order (LBO) analysis, aromaticity studies, and enthalpy of formation calculations were performed. Patterns observed in the computational results were summarized, and their potential correlations were analyzed. Based on these findings, design recommendations for derivatives of these skeletons in energetic compounds were proposed to serve as a reference for energetic material chemists.

## 1. Introduction

Energetic materials, with their high energy density, rapid energy release rate, and portability, have become indispensable in the military and aerospace fields. They also have significant applications in civilian fields such as civil engineering and mining. Since the time of Alfred Bernhard Nobel, generations of energetic material scientists have been dedicated to developing materials with superior performance. The energetic derivatization of nitrogen heteroaromatic rings is currently the leading approach in designing and synthesizing new energetic compounds [1,2]. This trend stems from the balanced energy and stability of nitrogen heteroaromatic rings. Compared to pure carbon skeletons, the C-N, N-N, and N=N bonds in nitrogen heteroaromatic rings release more energy during decomposition and recombination, while their aromaticity provides additional stability. Notable energetic modifications of nitrogen heteroaromatic rings include NTO (nitrotriazolone) [3] and TNP (3,4,5-trinitropyrazole) [4] based on azole ring skeletons, and LLM-105 (2,6-diamino-3,5-dinitropyrazine-1-oxide) [5] and ICM-102 (2,4,6-triamino-5-nitropyrimidine-1,3-dioxide) [6] based on azine ring skeletons. These explosive molecules are designed and synthesized based on single nitrogen heteroaromatic rings.

Subsequently, energetic material chemists have expanded their focus to a broader chemical space involving bridged and fused nitrogen heteroaromatic rings, achieving significant outcomes. Energetic compounds based on fused nitrogen heteroaromatic rings typically possess high density and structural stability, making them ideal for designing heat-resistant explosives and high-energy insensitive explosives. In this field, Professors Yang [7,8] and Tang [9] from Nanjing University of Science and Technology and Pang [10] from Beijing Institute of Technology have made outstanding contributions.

Among fused skeletons, the five-membered fused six-membered nitrogen heteroaromatic ring skeleton is the most common due to its simplicity in synthesis and structural stability. The number of such skeletons is relatively limited. We found that there are only 213 five-membered fused six-membered aromatic ring skeletons with one to four nitrogen atoms, making a comprehensive and systematic study of this skeleton both feasible and necessary, particularly to investigate the impact of different numbers and positions of nitrogen atoms on the skeleton’s energy and stability.

In this study, we use Density Functional Theory (DFT) to perform natural population analysis [11] (NPA), Laplacian bond order [12,13] (LBO) analysis, aromaticity studies (localized orbital locator function for π-electrons [14,15] (LOL-π), multicenter bond order [16] (MCBO)), enthalpy of formation (EOF) calculations, and correlation studies of related properties. We summarize the relevant patterns, propose design suggestions for energetic compounds based on these skeletons, and aim to provide a reference for energetic material chemists.

## 2. Result and Discussion

### 2.1. NPA Charges Analysis

**Figure 1:**
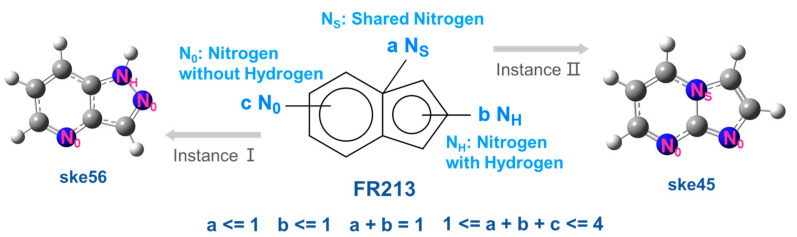
Schematic diagram of three types of nitrogen atoms in FR213; a, b, and c represent the number of N_S_, N_H_, and N_0_ atoms, respectively.

As shown in Figure 1, the nitrogen atoms in FR213 can be classified into three types. The first type consists of nitrogen atoms on C-N bonds shared by fused rings, labeled as N_S_. The second type includes nitrogen atoms that form N-H bonds, labeled as N_H_. The third type comprises nitrogen atoms where the bonds at both ends are single and double bonds, labeled as N_0_. The lone pairs of the first two types of nitrogen atoms (N_S_ and N_H_) participate in the conjugation of the ring system, with their electron-donating conjugation effect being greater than their electron-withdrawing inductive effect. The lone pairs of the third type of nitrogen atom (N_0_) are perpendicular to the π orbitals and do not participate in ring system conjugation, exhibiting electron-withdrawing inductive and conjugation effects. In organic chemistry, N_S_ and N_H_ are referred to as pyrrole-like nitrogen atoms, while N_0_ is referred to as pyridine-like nitrogen atoms. It is important to note that the requirement to maintain the aromaticity of the skeleton molecule stipulates that N_S_ and N_H_ are mutually exclusive. That is, if a skeleton contains N_S_, it cannot contain N_H_, and vice versa. Their total number can only be 1.

**Figure 2:**
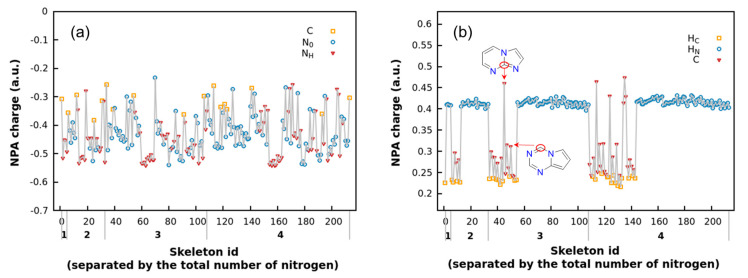
The most negative (**a**) and positive (**b**) NPA charge values of each skeleton.

Natural population analysis (NPA) [11] was conducted on FR213, and the population diagrams with specific charge values for each skeleton are shown in Figure S2, with the corresponding color mapping provided in Figure S3. To investigate the relationship between NPA charge distribution and skeleton structure, the most negative and most positive NPA charges of FR213, along with their corresponding atomic types, are visualized in Figure 2. It can be observed that no significant differences are found between the skeletons with varying numbers of substituted nitrogen atoms.

As shown in Figure 2a, the most negative charges correspond to three atomic types, C, N_0_, and N_H_, with the general trend of corresponding charge values being N_H_ < N_0_ < C. Combining this with the structural analysis of each skeleton, we find that the atomic types associated with the most negative charges are determined by the following patterns:

(1) Electronegativity trend: N > C > H.
(2) N_H_ tends to carry more negative charge due to the electron-donating effect of the H atom.
(3) Catenated nitrogen substructures (N3 or N4) reduce the negative charge on nitrogen atoms compared to isolated nitrogen atoms. Nitrogen atoms at the ends of catenated nitrogen substructures carry more negative charge, whereas those in the middle exhibit less.
(4) Both shared nitrogen (N_S_) and carbon (C_S_) atoms exhibit less negative charge compared to their isolated counterparts of the same type.
(5) For the same type of atom, those in five-membered rings carry more negative charge than those in six-membered rings.
(6) Charges tend to alternate in distribution; that is, if an adjacent atom carries a higher positive charge, the atom in question is likely to carry more negative charge.

Using the patterns described above, the atom corresponding to the most negative charge in a framework can be roughly identified. For example, if a framework molecule contains an N_H_ group on a five-membered ring that is not part of a consecutive nitrogen substructure, it can typically be identified as the atom with the most negative charge. Similarly, nitrogen atoms on a five-membered ring near a shared bond often carry the most negative charge, as they are in close proximity to C_S_ atoms, which carry more positive charge.

As shown in Figure 2b, most of the atoms with the highest positive charge are hydrogen atoms attached to nitrogen (H_N_), with a smaller number being carbon (C) or hydrogen atoms attached to carbon (H_C_). In fact, whenever an N-H bond is present, the atom with the highest positive charge will always be H_N_. In the absence of an N-H bond, the atom with the highest positive charge could be either H_C_ or a carbon atom located between two or three nitrogen atoms. The patterns used to determine the most negatively charged atom can also assist in identifying the most positively charged atom. For instance, when no N-H bond is present, a C_S_ atom located between two nitrogen atoms can typically be identified as the atom with the highest positive charge.

### 2.2. Laplacian Bond Order Analysis

**Figure 3:**
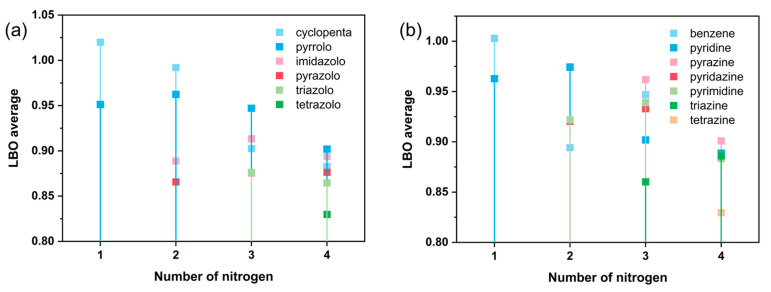
FR213 includes different numbers of nitrogen atoms subdivided by ring type for average LBO_min-noH_; (**a**) subdivided by five-membered ring type; (**b**) subdivided by six-membered ring type.

Laplacian bond order (LBO), proposed by Lu [12] and particularly suitable for studying covalent bonding, has been demonstrated to exhibit a strong linear correlation with bond dissociation energies [13] (BDE), which is advantageous for analyzing cyclic systems where calculating BDEs is challenging. Therefore, we primarily use LBO to assess the structural stability of FR213. As depicted in Figure 3, there is a general trend where the average minimum skeleton LBO (LBO_min-noH_) gradually decreases as the number of nitrogen atoms in the skeleton increases. This trend becomes more pronounced when nitrogen atoms are present in six-membered rings. Six-membered rings, being six-center six-electron systems, exhibit lower average electron density compared to the five-center six-electron systems of five-membered rings. The introduction of electron-withdrawing pyridine-like nitrogen intensifies the electron deficiency effect, further reducing the system’s stability.

As shown in Figure S4, the bond corresponding to the minimum LBO (LBO_min_) is most often the N-H bond when N_H_ is present. Excluding N-H or C-H bonds and considering only the bonds within the ring skeleton, the LBO values generally follow the order C-C > C-N > N-N, which aligns with chemical knowledge. However, when a molecule’s Lewis structure contains a double N-N bond (i.e., N=N), the LBO of the N-N bond can exceed that of C-N bonds. Additionally, when N_S_ is present, the smallest C-N bond is always the shared C-N bond, likely because the connected carbon atom has relatively fewer charges.

Excluding N-H and C-H bonds, the average LBO_min-noH_ of skeletons with N_H_ is greater than those without N_H_. This is due to the electron-donating inductive effect of hydrogen in N-H bonds, which strengthens the C-N or N-N bonds involving the nitrogen in the N-H bond. Moreover, the C-N bond in N_S_ skeletons often becomes the weakest bond, with its LBO being smaller than other C-N bonds. Therefore, in terms of bond stability within the ring skeleton, excluding N-H bonds and focusing only on LBO_min-noH_, skeletons with N_H_ have an advantage. However, N_H_-type skeletons include weaker N-H bonds, potentially leading to proton transfer and making the entire ring unstable.

To investigate the effect of the relative position of nitrogen atoms on bond strengths, we conducted a statistical analysis comparing the LBO of skeletons that include and exclude catenated nitrogen substructures. First, we examined the average LBO of the weakest N-N bonds (average LBO_min-N-N_) in three nitrogen-substituted skeletons, considering both cases with and without the N3 substructure. It was found that when the N3 substructure was present, the average LBO_min-N-N_ was 0.912, while without the N3 substructure, it was 0.914. Similarly, no statistically significant differences were observed in the average weakest LBO for other bond types. Next, we analyzed four nitrogen-substituted frameworks. When the N3 substructure was included, no statistically significant differences were observed in the average weakest LBO for various bond types. However, when the N_4_ substructure was included, the average LBO_min-N-N_ was 0.868, compared to 0.898 when the N_4_ substructure was absent, showing a noticeable difference. For other bond types, no statistically significant differences were found.

**Figure 4:**
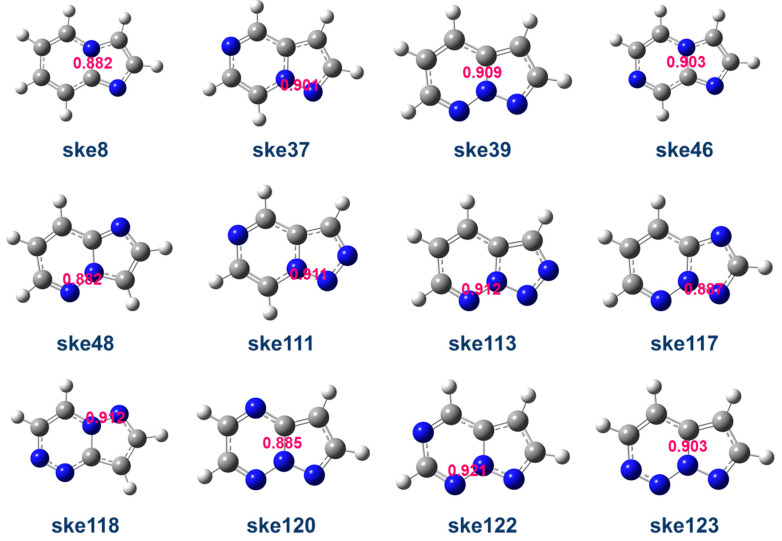
The top 12 skeletons sorted by LBO_min-noH_ in FR213 (blue = N, gray = C, white = H).

We observed that the LBO of C-H bonds is not significantly affected by the number or position of substituted nitrogen atoms, and their average value of 0.872 can be considered a benchmark. When a framework with the in-ring bonds whose LBO is greater than 0.872 decomposes, it tends to lose protons first rather than undergo ring opening and can thus be considered a relatively stable structure. Figure 4 shows 12 skeleton molecules that meet this criterion, which we regard as ideal skeletons for energetic materials from a structural stability perspective. It should be noted that molecules containing N_H_ were not considered in this selection.

### 2.3. LOL-π Analysis

To qualitatively assess the aromaticity of FR213, we used the localized orbital locator function for π electrons (LOL-π) and generated color-filled plots. These plots were created using the Multiwfn program, taking the LOL-π values at 1 Å above the plane of the skeleton. The results are shown in Figure S5. The blue-green-red color scale corresponds to LOL-π values in the range of 0 to 0.8.

It can be observed that the LOL-π paths traverse the entire skeleton in all molecules, indicating varying degrees of aromaticity. Certain bond regions display deeper red colors, indicating areas with higher concentrations of π electrons. These regions correspond to the double-bond areas in the Lewis structures of the skeleton molecules. Since all molecules in FR213 have only one resonance structure, this single Lewis structure effectively reflects the distribution of bond strength. Double-bond regions contain more π bond components, corresponding to richer π electron densities.

### 2.4. Multicenter Bond Order Analysis

The multicenter bond order (MCBO) can be used to quantitatively analyze the aromaticity of FR213, and normalized MCBO (NMCBO) can compare the aromaticity of rings of different sizes. As shown in Table 1, the presence of N_H_ atom leads to a decrease in the average NMCBO-5R (where NMCBO-5R refers to the NMCBO of five-membered rings; similarly, NMCBO-6R refers to the NMCBO of six-membered rings). N_H_-6R decreases both the average NMCBO-6R and NMCBO-5R, while N_H_-5R significantly increases the average NMCBO-6R. This indicates that N_S_ is beneficial for the aromaticity of five-membered rings, N_H_-5R enhances the aromaticity of six-membered rings, and N_H_-6R is detrimental to the aromaticity of six-membered rings. Generally, NMCBO-5R and NMCBO-6R both tend to decrease with an increasing number of nitrogen atoms in the six-membered ring. However, when N_H_-5R is present, NMCBO-6R generally increases and no longer follows this trend. By analyzing these patterns, we can infer that the placement and type of nitrogen atoms in the fused ring systems significantly influence the aromaticity and stability of the compounds, as measured by NMCBO values.

### 2.5. Enthalpy of Formation Analysis

**Figure 5:**
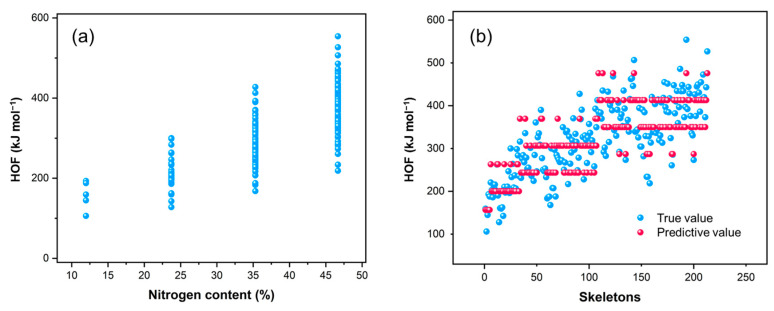
(**a**) Distribution of enthalpy of formation for FR213 with different nitrogen content; (**b**) scatter plot comparing predicted values from Equation (1) with true values.

The enthalpy of formation for FR213 was calculated using the definition method (for calculation details, see the SI). As shown in Figure 5, the overall trend indicates that the enthalpy of formation for the skeleton increases with increasing nitrogen content (Figure 5a), which is consistent with chemical intuition. Similarly, the density of the skeletons shows a comparable pattern (Figure S6).

We performed a multiple linear regression of the number of each type of bond in the skeleton against the enthalpy of formation, resulting in the fitting formula shown in Equation (1). Here, *B_CN_* represents the number of C-N bonds, *B_NN_* represents the number of N-N bonds, and *B_CC_* represents the number of C-C bonds. Since all bonds in the ring skeletons are aromatic, we do not strictly distinguish between single and double bonds. The regression shows a very good correlation, with a correlation coefficient *R*^2^ = 0.98521 and a root mean square error (RMSE) of 40.4 kJ mol^−1^. Figure 5b compares the predicted values from the fitting formula with the actual values.

From the coefficients of each term in Equation (1), we can infer the relative contribution of each type of bond to the enthalpy of formation. The order of contribution is N-N > C-N > C-C, with the coefficient for C-N bonds being three times that of C-C bonds and the coefficient for N-N bonds being ten times that of C-C bonds. This highlights the significant contribution of nitrogen-involved chemical bonds to the enthalpy of formation.

(1) $$HOF=33.04B_{CN}+117.72B_{NN}+11.31B_{CC}$$

In tetra-nitrogenous skeletons, when the number of N-N bonds is two, comparing two different arrangements of nitrogen atoms—either forming a direct N-N bond with N3 or having two separate N-N bonds—the corresponding average molecular enthalpies of formation are 404.9 and 421.6 kJ mol^−1^, respectively. This indicates that under the same nitrogen content and N-N bond count, the linear chain structure of nitrogen atoms does not always exhibit higher energy than expected. Additionally, when comparing the average enthalpies of formation in tetra-nitrogenous skeletons with and without N-H bonds, they are 379.6 and 379.0 kJ mol^−1^, respectively. This suggests that N-H bonds have minimal impact on energy levels.

**Figure 6:**
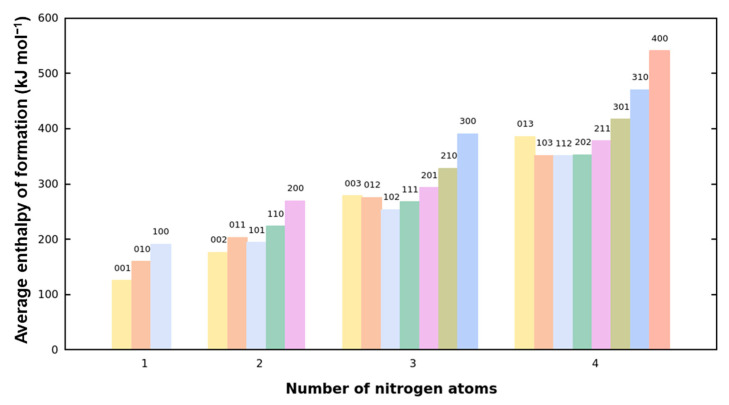
Average EOF of skeletons composed of different nitrogen atom types. The numbers at the top of each bar represent the nitrogen atom composition, with the hundreds digit indicating the number of N_6_ atoms, the tens digit representing the number of N_S_ atoms, and the ones digit corresponding to the number of N_5_ atoms. The data are grouped by the total number of nitrogen atoms.

We found that nitrogen atoms in six-membered rings (N_6_), five-membered rings (N_5_), and shared nitrogen atoms (N_S_) contribute differently to the EOF of skeleton molecules. N_6_ contributes the most, N_5_ the least, and NS falls in between. Figure 6 shows the histogram of average EOF for skeletons composed of different nitrogen atom types. The numbers at the top of each bar represent the nitrogen atom composition: the hundreds digit indicates the number of N_6_ atoms, the tens digit represents the number of N_S_ atoms, and the ones digit corresponds to the number of N_5_ atoms. It can be observed that the average EOF generally increases with the composition number, supporting our findings. However, there are some exceptions to this trend, which can be attributed to certain nitrogen atom compositions containing more N-N bonds, thereby releasing additional energy. In the LBO analysis, we noted that an increase in N_6_ relative to N_5_ leads to a more significant decrease in stability. This is consistent with the fact that N_6_ provides higher energy, offering an explanation for this behavior.

### 2.6. Correlation Analysis

**Figure 7:**
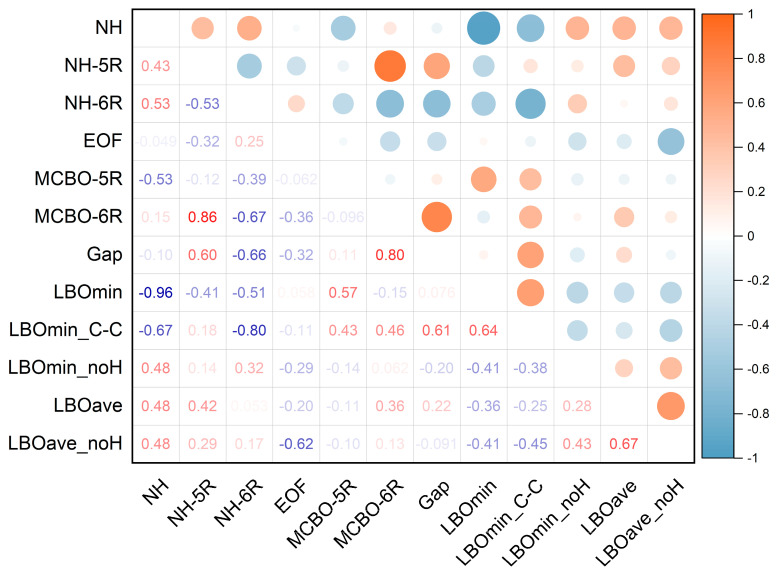
Pearson correlation heatmap of FR213-related properties. NH: N_H_ atom; NH-5R: N_H_ atom located in a five-membered ring; NH-6R: N_H_ atom located in a six-membered ring; EOF: Enthalpy of formation; MCBO-5R: MCBO of a five-membered ring; MCBO-6R: MCBO of a six-membered ring; Gap: HOMO-LUMO gap; LBOmin: Minimum LBO among all bonds; LBOmin_C-C: Minimum LBO of the C-C bond; LBOmin_noH: Minimum LBO of all bonds excluding C-H and N-H bonds; LBOave: Average LBO of all bonds; LBOave_noH: Average LBO of all bonds excluding C-H and N-H bonds.

To explore the potential correlations among the structural features, aromaticity, stability, and HOMO-LUMO gap of FR213, we conducted a Pearson correlation analysis with a significance level of 0.05. As shown in Figure 7, N_H_ is strongly negatively correlated with the minimum LBO value, indicating that when N_H_ is present, the weakest bond in the molecule tends to be the N-H bond. Moreover, N_H_ shows moderate positive correlations with LBO_min-noH_, LBO_ave-noH_, and LBO_ave_ of the skeleton rings, suggesting its enhancing effect on other bonds within the skeleton. These findings align with the conclusions from our LBO analysis in Section 2.2.

Additionally, we observed that N_H_ is negatively correlated with the MCBO of the five-membered rings, particularly on six-membered rings, where it exhibits a more pronounced negative correlation with MCBO. This indicates that N_H_ on six-membered rings simultaneously reduces the aromaticity of five- and six-membered rings. Interestingly, N_H_ on five-membered rings shows a positive correlation with the MCBO of six-membered rings.

Furthermore, the MCBO of six-membered rings shows a weak negative correlation with the enthalpy of formation (EOF), implying that the enhanced aromaticity of six-membered rings slightly lowers the molecule’s energy. Additionally, there is a significant positive correlation between the MCBO of six-membered rings and the HOMO-LUMO gap, suggesting that the aromaticity of six-membered rings has a greater impact on the overall stability of the molecule. LBO_min_C-C_ shows a significant negative correlation with NH-6R (correlation coefficient of −0.80), while it exhibits moderate positive correlations with MCBO-5R and MCBO-6R (correlation coefficients of 0.43 and 0.46, respectively). The underlying mechanism may be that NH-6R reduces the aromaticity of the skeleton, thereby indirectly weakening the C-C bond.

Therefore, from the perspective of enhancing the aromatic stability of the skeleton molecule, it is advisable to minimize the introduction of N_H_ on six-membered rings when designing energetic materials.

## 3. Computing Method

Using SMILES encoding [17], we enumerated and deduplicated one to four nitrogen-containing five-membered fused six-membered skeletons with a self-developed program. We then used the Open Babel 3.1.1 program [18] to remove skeletons that did not satisfy aromaticity criteria, ultimately obtaining 213 five-membered fused six-membered nitrogen heteroaromatic ring skeletons. For ease of discussion, we name this system FR213 (where FR stands for Fused Rings), and the 213 skeletons are labeled with a numerical sequence prefixed by “ske”. Their 2D structures are shown in Figure S1. The 3D structures were geometrically optimized and subjected to vibrational analysis at the M062X/def2TZVP level using the Gaussian 09 program [19]. All subsequent analyses are based on the optimized structures. NPA charges were calculated using the NBO module of Gaussian 09, while LBO, LOL-π, and MCBO analyses were performed using the Multiwfn 3.8 program [20,21].

## 4. Summary and Recommendations

Nitrogen, as an element with properties distinctly different from carbon, when introduced into five-membered and six-membered aromatic carbon frameworks, can cause significant changes in the properties of the original framework. The number and positions of nitrogen atoms can lead to subtle differences in the reactivity, stability, and energy of the molecular frameworks. Through systematic calculations and analyses of all 213 five-membered and six-membered aromatic nitrogen-containing frameworks (FR213), ranging from mono-nitrogen to tetra-nitrogen substitutions, we have derived some general patterns.

(1) The nitrogen atoms in FR213 can be classified into three types, N_S_, N_H_, and N_0_, as described in the main text. They exhibit completely different conjugative and inductive properties. Different atomic types and their relative positions can have subtle effects on the NPA charge population of the skeletons.
(2) In the context of LBO analysis, the LBO_min_ decreases as the number of nitrogen atoms increases, suggesting a reduction in the stability of the framework with higher nitrogen content. This trend is particularly pronounced when nitrogen atoms are incorporated into six-membered rings. N-H bonds are unequivocally the weakest bonds in the framework, yet their presence enhances the strength of other bonds in the framework. The LBO values of the bonds in the framework rings generally follow the trend C-C > C-N ≈ N=N > N-N. Among the C-N bonds, C-N_S_ is the weakest. Besides, the N3 substructure does not have a significant effect on bond strength, but the N4 substructure does result in relatively weaker N-N bonds.
(3) All molecules in FR213 have unique Lewis structures, and parameters such as bond length, LBO, and LOL-π paths can be associated with these structures. For example, regions corresponding to double bonds in the structure will exhibit shorter bond lengths, higher LBO values, and greater π electron density. Therefore, the strength of bonds can be qualitatively discussed based on the Lewis structures of the skeleton molecules.
(4) In the context of MCBO analysis, the presence and position of N-H have a significant impact on the aromaticity of the framework molecules. N-H on the five-membered ring enhances the aromaticity of the six-membered ring, while N-H on the six-membered ring weakens the aromaticity of both the five-membered and six-membered rings. The MCBO of the six-membered ring shows a clear positive correlation with the HOMO-LUMO gap.
(5) The EOF of FR213 is strongly correlated with the composition of its chemical bonds. By fitting the EOF to the quantities of C-C, C-N, and N-N bonds, we obtain a fitting equation with R^2^ = 0.98521 and RMSE = 40.4 kJ mol^−1^. The coefficients of this equation reflect the relative contributions of each bond type to the EOF. Specifically, the coefficient for the C-N bond is three times that of C-C, and the coefficient for the N-N bond is ten times that of C-C, indicating a significant contribution of nitrogen-containing bonds to the EOF. Furthermore, for a given bond composition, longer nitrogen chains do not result in a higher EOF. The presence or absence of N-H bonds has minimal impact on the EOF of the framework. Additionally, for the contribution to EOF: N_6_ > N_S_ > N_5_.

Based on these patterns, we can make some recommendations for designing energetic compounds based on such frameworks:

(1) In the synthesis of elemental explosives, skeletons containing N-H bonds are undesirable. Although N-H bonds can strengthen the framework, the N-H bond itself is the most unstable component, potentially leading to instability in explosive molecules, manifested as acidity or high sensitivity.
(2) Achieving a balance between energy and stability depends on the distribution of nitrogen atom positions. While there is a general trend of decreasing stability with increasing nitrogen content, as shown in Figure 4, many tri-nitrogen and tetra-nitrogen frameworks can still maintain good structural stability. Specifically, placing more than two nitrogen atoms on six-membered rings should be avoided, as nitrogen atoms on these rings have a more detrimental effect on the framework’s stability. Moreover, continuous nitrogen chain structures do not confer energetic benefits and should be avoided in the framework to mitigate their negative impact on stability.

Overall, this research advances our understanding of nitrogen-containing aromatic frameworks and provides a foundational basis for the rational design of new energetic materials. By elucidating the relationships between structure, aromaticity, stability, and energetic properties, this work offers valuable insights for future synthetic and computational strategies aimed at optimizing the performance and stability of energetic compounds.

## Figures and Tables

**Table 1:** The average NMCBO for different types of six-membered rings within various skeleton types.

Skeleton Types	Six-Membered Rings	Average NMCBO-5R	Average NMCBO-6R
**N_H_-5R**	benzene	0.51567	0.57977
	pyridine	0.51199	0.58495
	pyrazine	0.50957	0.58365
	pyridazine	0.50939	0.58466
	pyrimidine	0.50873	0.58265
	triazine	0.50859	0.58706
	**Summary Average**	**0.51087**	**0.58411**
**N_H_-6R**	pyridine	0.52345	0.51568
	pyridazine	0.51281	0.51010
	pyrimidine	0.50961	0.50912
	pyrazine	0.49399	0.51652
	triazine	0.48808	0.50332
	tetrazine	0.46313	0.49413
	**Summary Average**	**0.50449**	**0.50922**
**N_S_**	pyrazine	0.54020	0.53373
	pyridazine	0.53737	0.52897
	pyridine	0.53629	0.53414
	triazine	0.52938	0.52845
	pyrimidine	0.52827	0.53232
	tetrazine	0.52249	0.52450
	**Summary Average**	**0.53163**	**0.53039**

## Data Availability

Data are contained within the article and Supplementary Materials.

## Supplementary Materials

The following supporting information can be downloaded at www.mdpi.com/article/10.3390/molecules30051101/s1; Figure S1: The 2D structures of FR213, along with their enthalpy of formation (in kJ mol^–1^), are shown below; Figure S2: The geometric structure of optimized FR213 with NPA annotation; Figure S3: NPA charge color mapping. Red represents negative values, and green represents positive values; Figure S4: The LBO visualization of FR213, where red dots represent the bonds with LBO_min_, and yellow dots represent the bonds with the LBO_min-noH_; Figure S5: The LOL-π color-filled plots of FR213; Figure S6: Distribution of densities for FR213 with different nitrogen content. Ref. [22] is cited in the Supplementary Materials.
