# Supplementary material for: DFT Study on Fused N-Heteroaromatic Frameworks: Stability, Aromaticity, and Energetic Insights from Five-Membered Fused Six-Membered N-Heteroaromatic Skeletons
Source: Molecules. 2025 Feb 27;30(5):1101. doi: 10.3390/molecules30051101 (PMC11901481; doi:10.3390/molecules30051101)
Supplement: Supplementary file 1 [file molecules-30-01101-s001.zip › molecules-3476166-supplementary.pdf]

Supplement Information

DFT Study on Fused *N*-Heteroaromatic Frameworks: Stability, Aromaticity, and Energetic Insights from Five-membered Fused Six-membered *N*-Heteroaromatic Skeletons

Zujia Lu [a], Cong Li [a], Shaoqun Li [a,b], Qiyao Yu [a,*] and Jianguo Zhang [a,*]

[a] *State Key Laboratory of Explosion Science and Technology, Beijing Institute of Technology, Beijing 100081, China.*

E-mail: zjgbit@bit.edu.cn; qiyaoyu@bit.edu.cn

[b] *State Key Laboratory of Transient Chemical Effects and Control, Shaanxi Applied Physics and Chemistry Research Institute, Xi'an 710061, Shaanxi, China.*

1-5

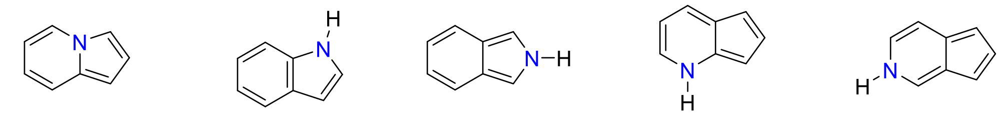

159.4 106.0 144.7 193.2 187.7

6-10

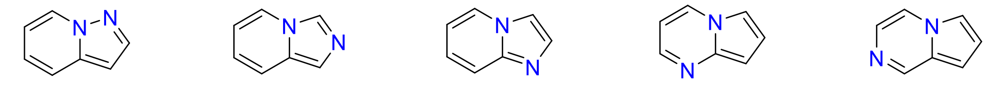

220.6 202.9 185.9 212.6 215.6

11-15

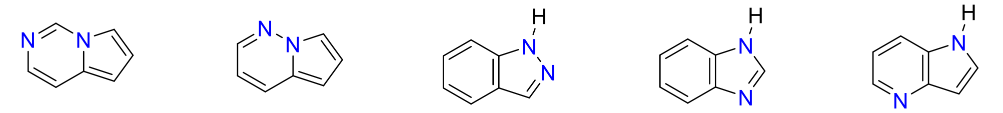

202.3 262.4 190.1 127.9 160.6

16-20

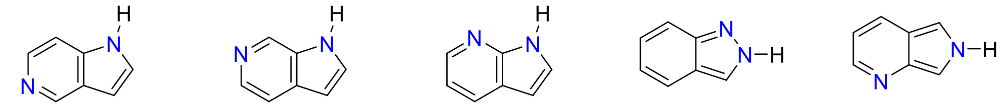

159.5 162.4 142.6 210.4 197.0

21-25

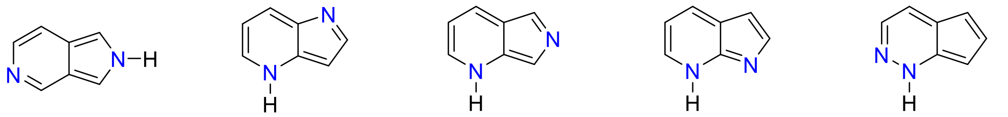

199.4 210.8 246.7 195.9 300.1

26-30

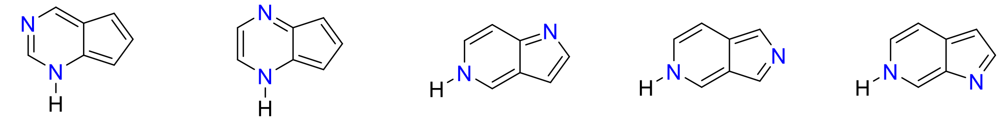

233.2 264.0 208.9 237.3 206.7

31-35

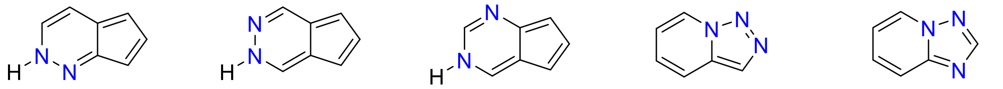

298.5 283.8 235.8 316.0 231.2

36-40

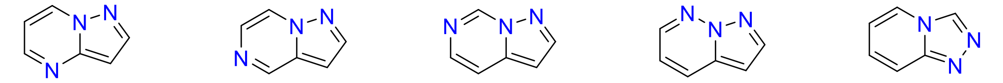

276.1 284.4 267.8 335.7 278.6

41-45

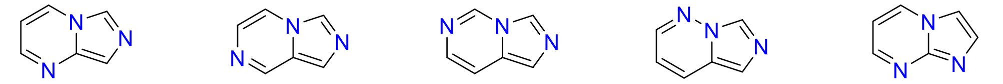

252.9 255.3 242.8 301.3 236.2

46-50

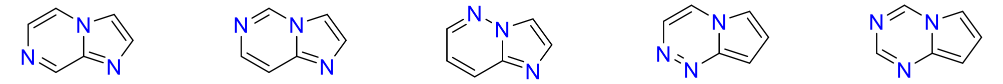

241.6 224.4 284.8 360.8 246.6

51-55

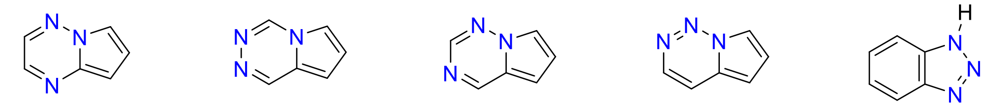

326.3 335.5 308.7 390.0 277.3

56-60

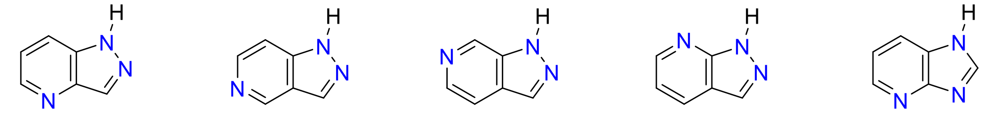

250.1 247.0 253.3 233.1 183.6

61-65

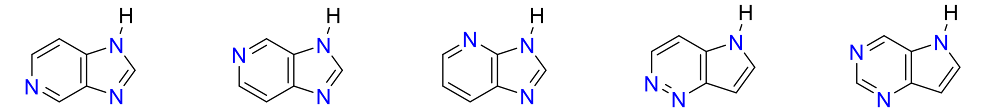

186.2 187.5 168.0 300.0 207.8

66-70

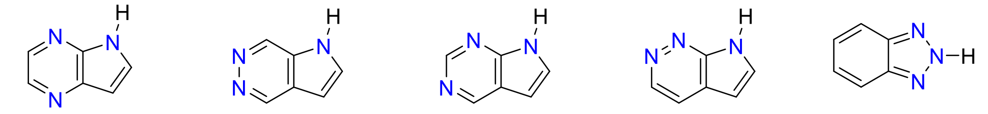

207.1 297.1 187.8 284.9 278.8

71-75

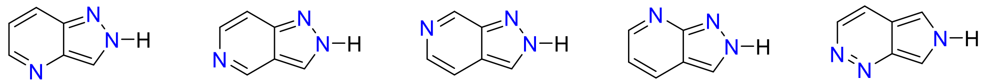

273.5 268.3 272.0 272.2 349.6

76-80

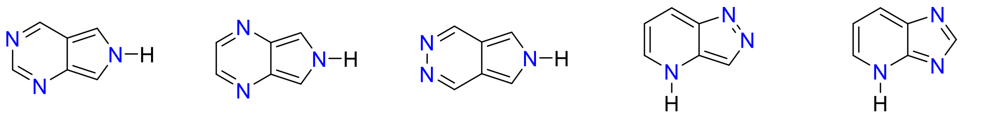

250.5 267.4 338.0 341.2 216.9

81-85

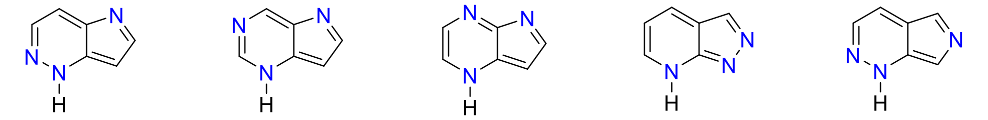

327.9 264.5 290.5 319.1 370.4

86-90

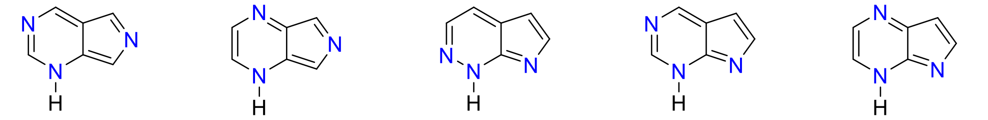

297.1 334.1 315.4 249.1 278.5

91-95

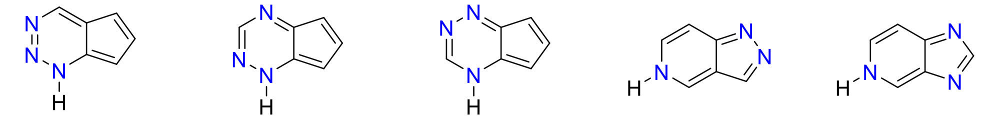

427.6 368.5 390.5 327.0 227.9

96-100

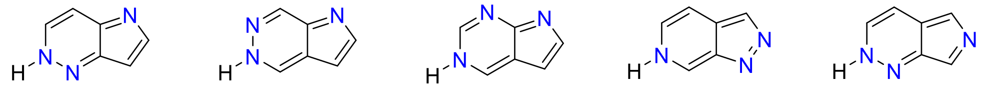

330.5 320.2 266.1 325.5 357.1

101-105

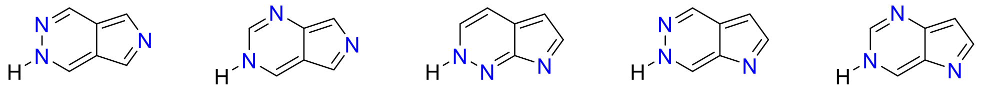

336.6 291.5 319.0 309.4 258.2

106-110

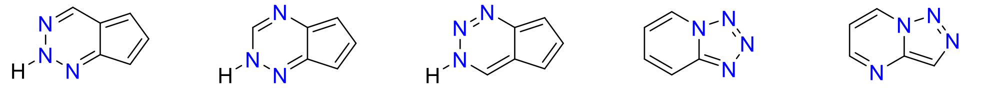

392.9 349.7 412.9 385.4 378.2

111-115

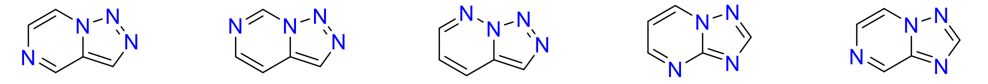

383.8 369.8 435.3 291.9 302.3

116-120

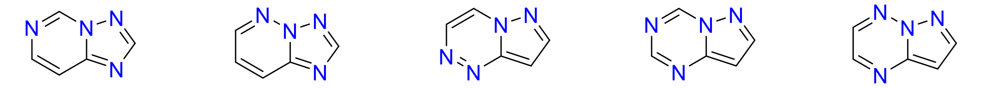

282.7 349.4 432.7 315.0 401.6

121-125

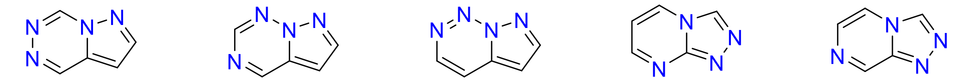

410.0 389.3 468.4 342.8 348.2

126-130

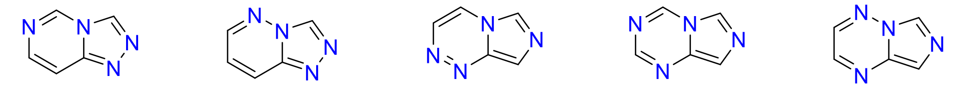

329.8 390.6 406.9 292.3 371.1

131-135

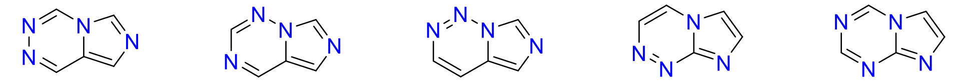

379.7 352.3 435.2 393.4 273.4

136-140

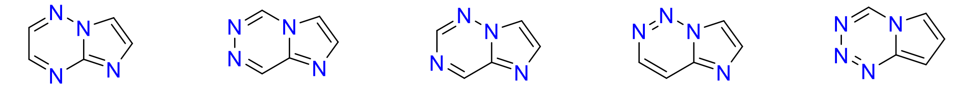

353.5 366.8 339.3 415.6 462.2

141-145

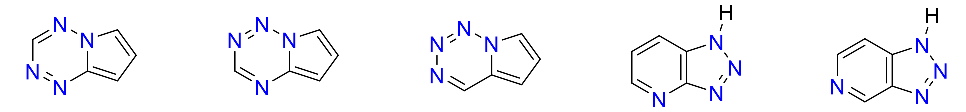

464.0 446.2 506.6 339.4 340.0

146-150

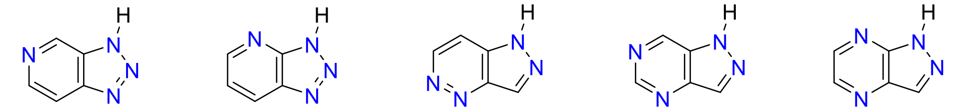

344.0 325.7 394.6 304.5 304.4

151-155

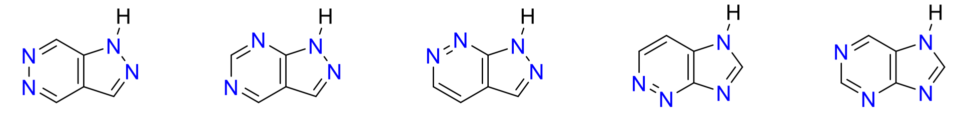

390.9 281.9 384.7 329.1 233.9

156-160

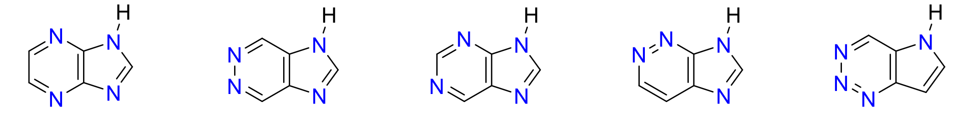

233.7 328.3 218.6 313.7 420.5

161-165

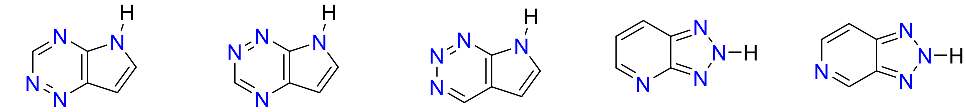

338.1 340.7 403.7 336.4 344.2

166-170

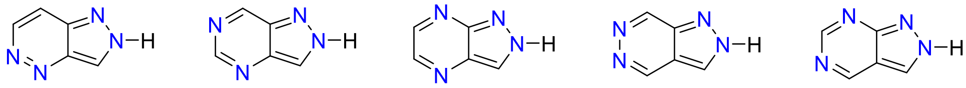

414.2 319.3 330.6 407.6 313.4

171-175

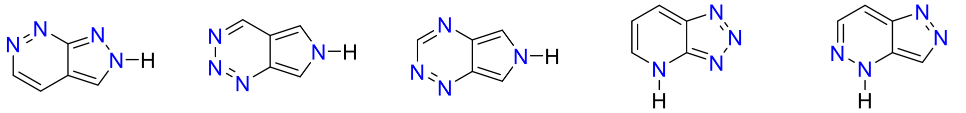

418.2 455.0 396.8 386.3 451.5

176-180

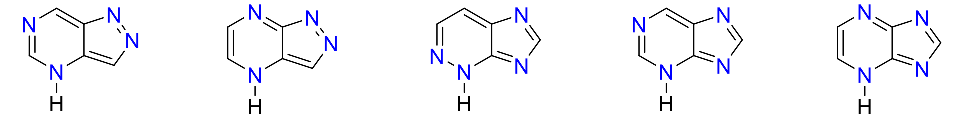

384.7 417.2 324.3 260.7 285.6

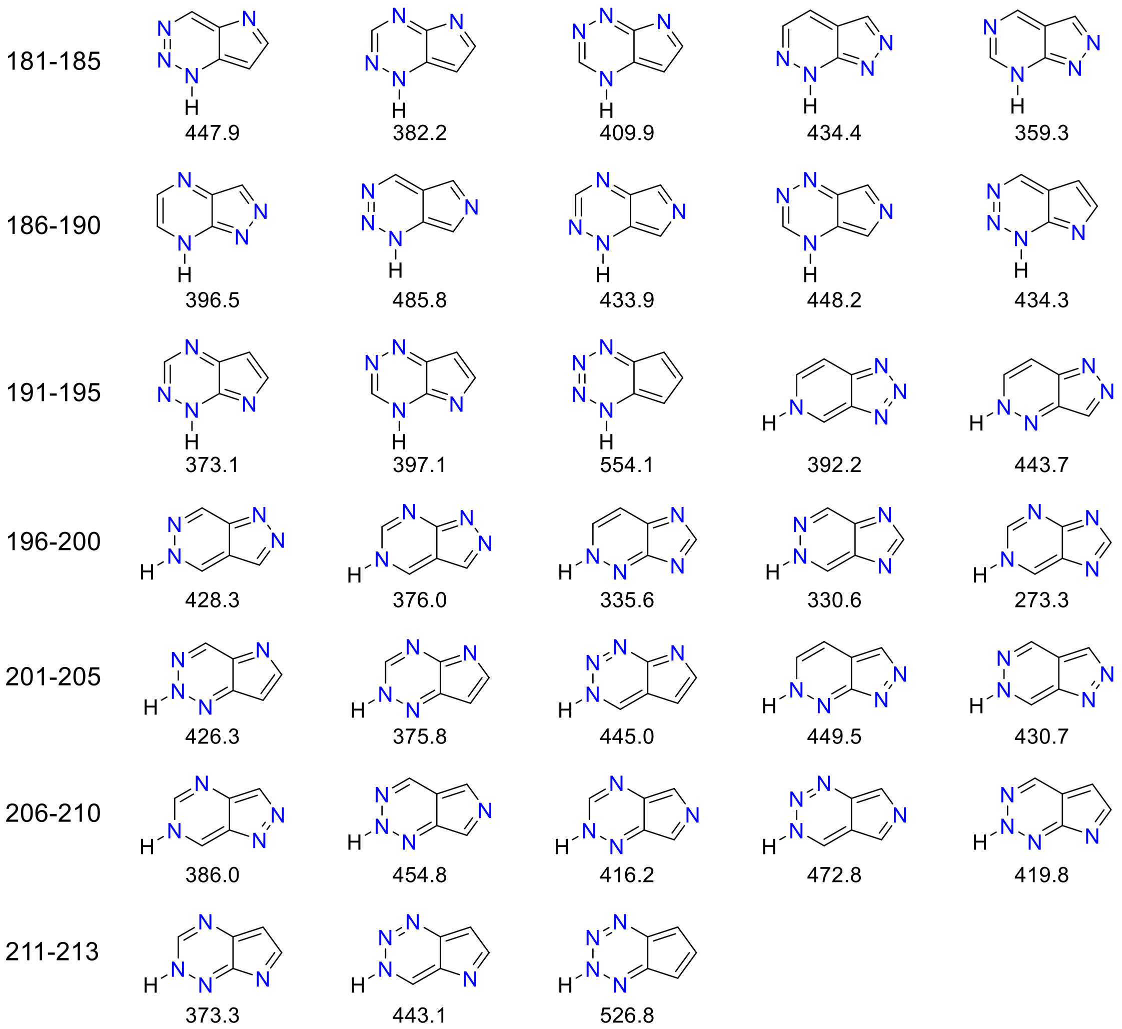

Figure S1 The 2D structures of FR213, along with their enthalpy of formation (in kJ mol^{-1}), are shown below.

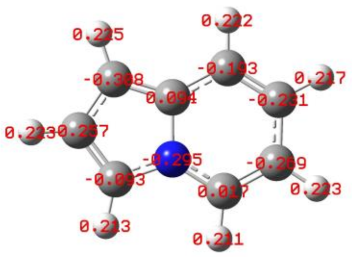

ske1

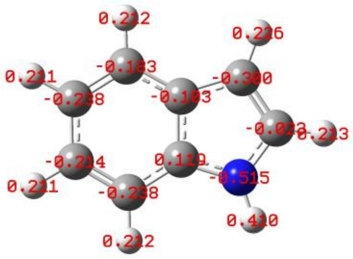

ske2

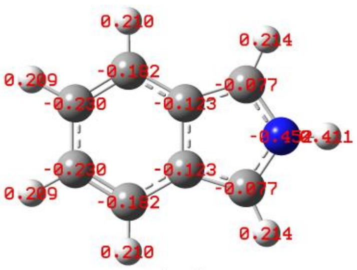

ske3

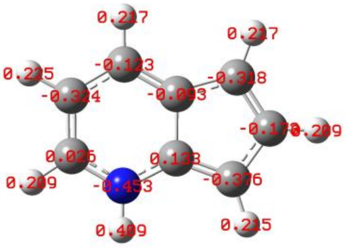

ske4

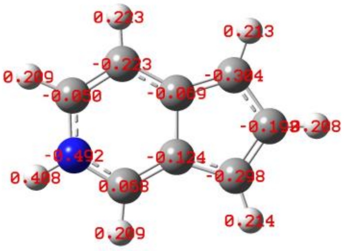

ske5

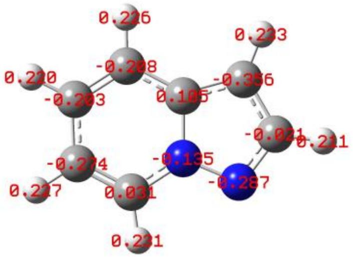

ske6

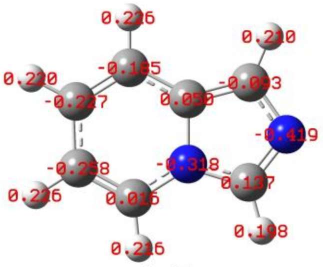

ske7

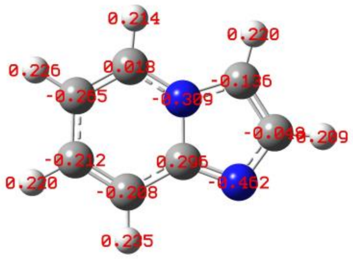

ske8

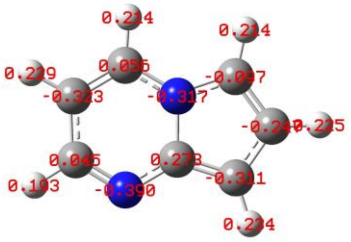

ske9

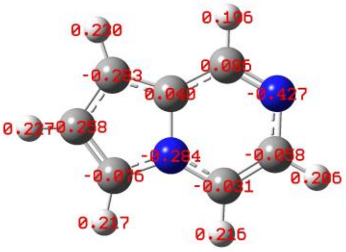

ske10

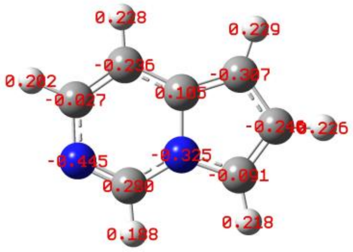

ske11

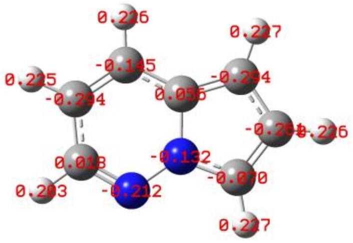

ske12

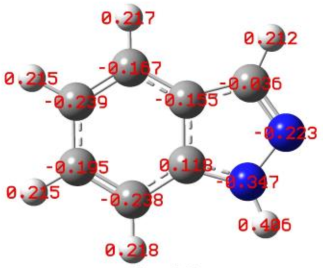

ske13

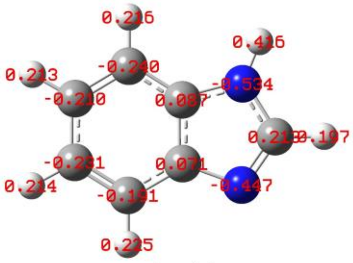

ske14

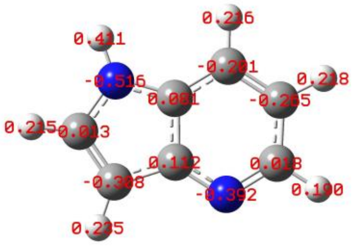

ske15

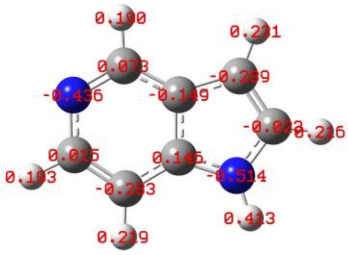

ske16

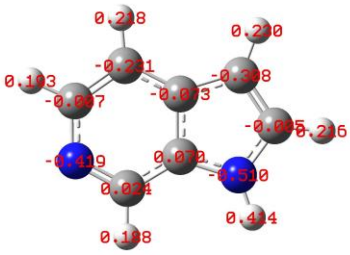

ske17

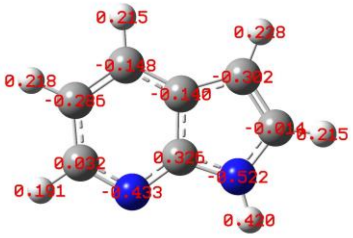

ske18

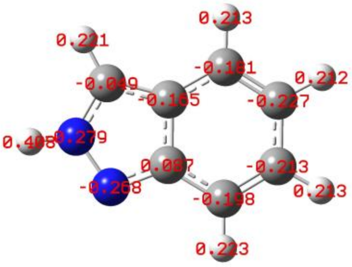

ske19

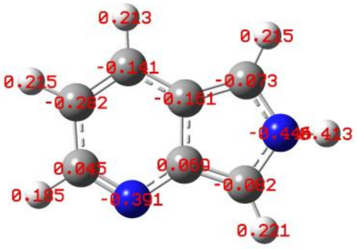

ske20

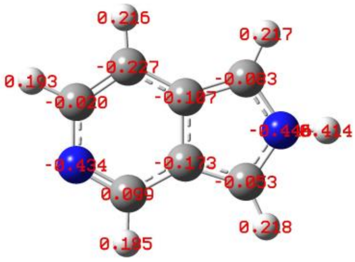

ske21

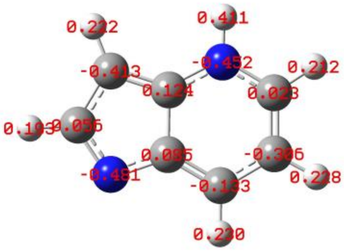

ske22

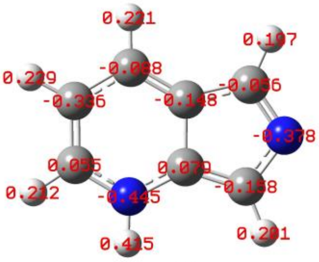

ske23

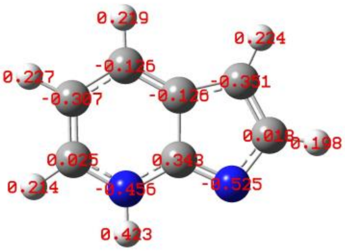

ske24

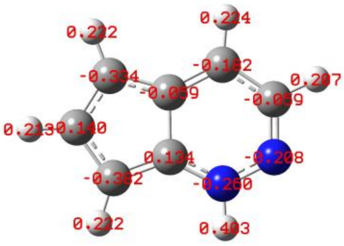

ske25

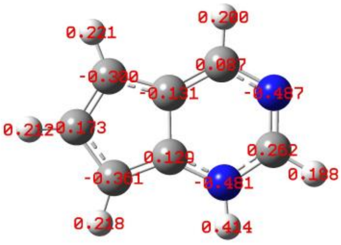

ske26

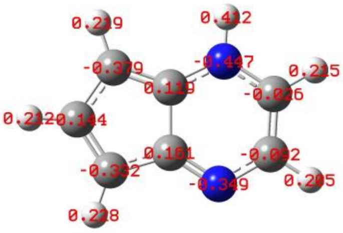

ske27

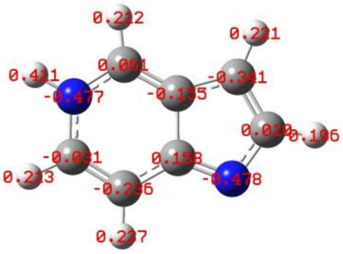

ske28

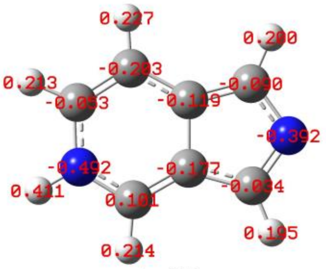

ske29

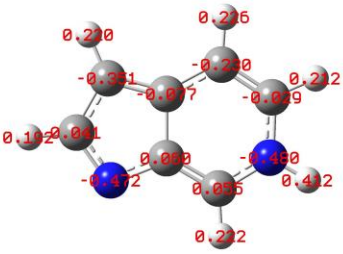

ske30

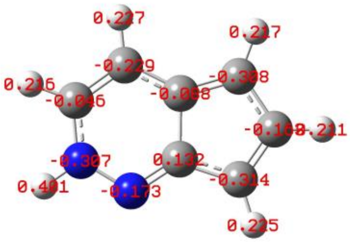
ske31

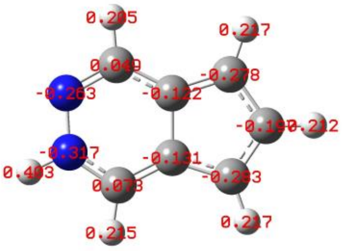
ske32

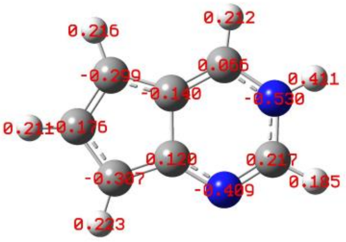
ske33

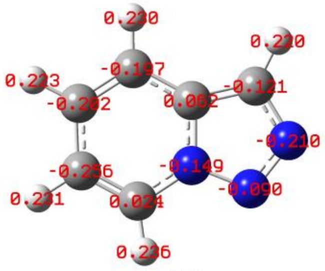
ske34

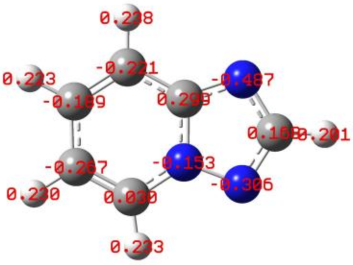
ske35

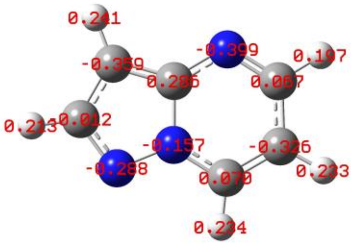
ske36

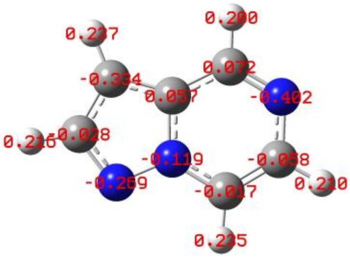
ske37

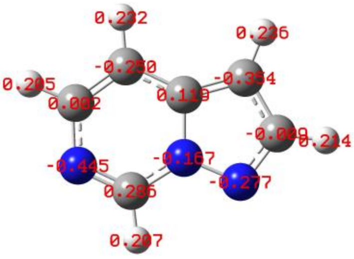
ske38

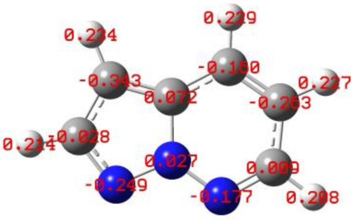
ske39

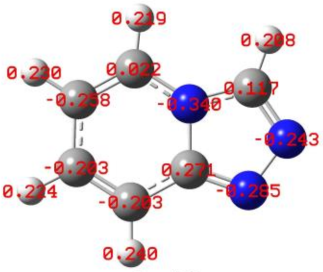
ske40

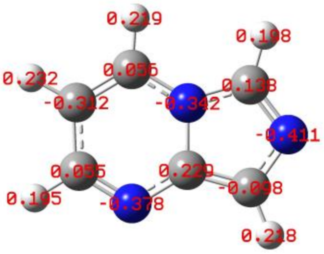
ske41

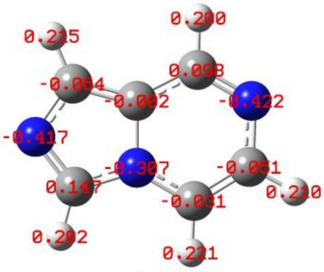
ske42

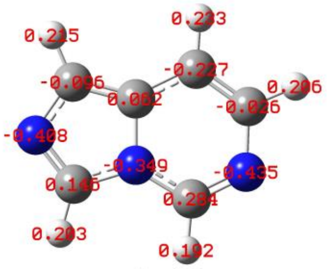
ske43

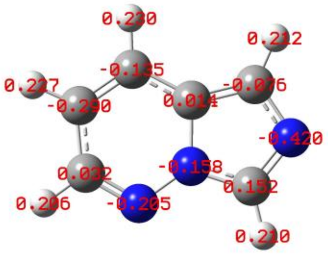
ske44

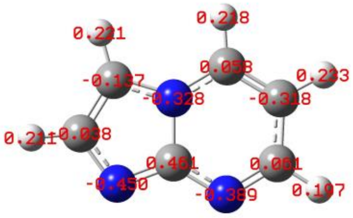
ske45

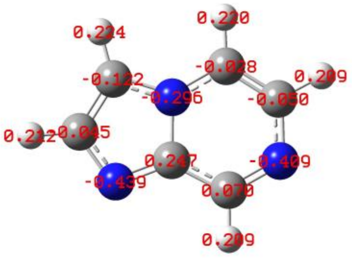

ske46

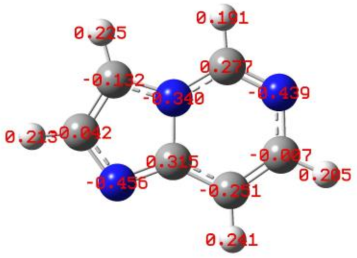

ske47

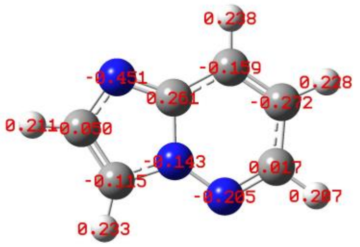

ske48

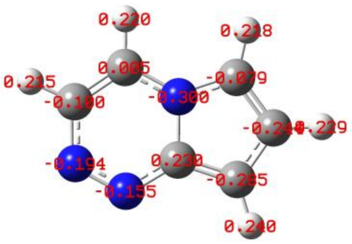

ske49

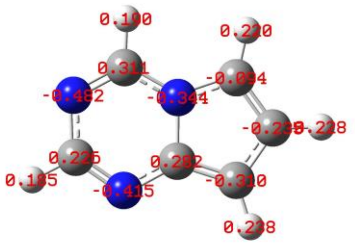

ske50

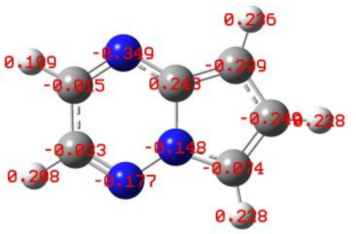

ske51

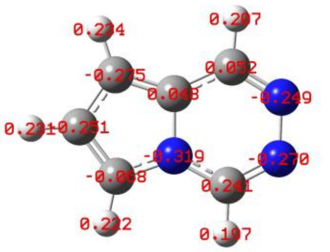

ske52

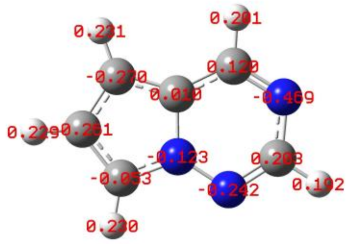

ske53

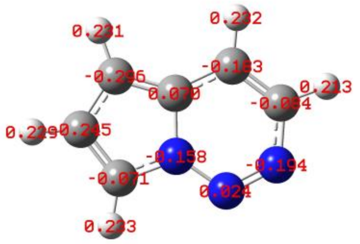

ske54

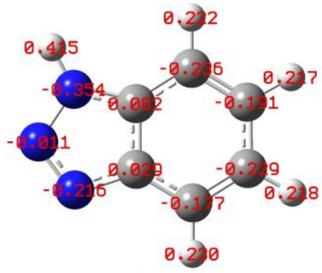

ske55

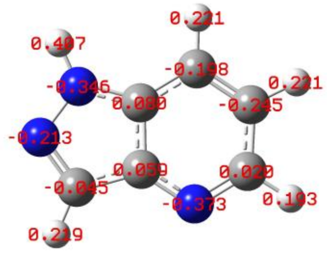

ske56

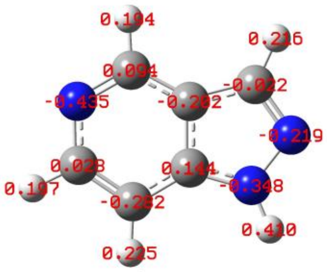

ske57

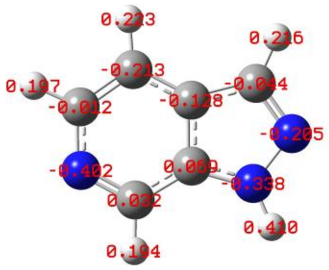

ske58

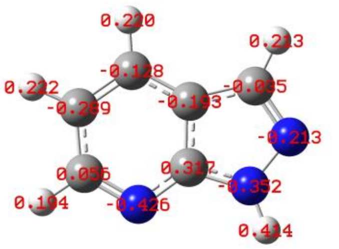

ske59

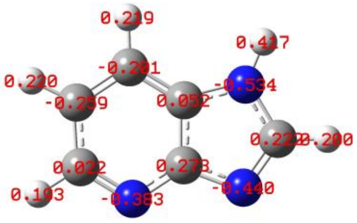

ske60

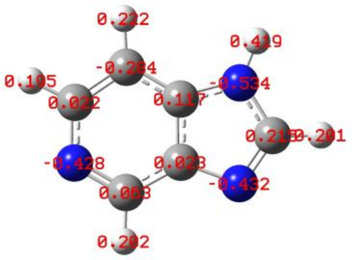
ske61
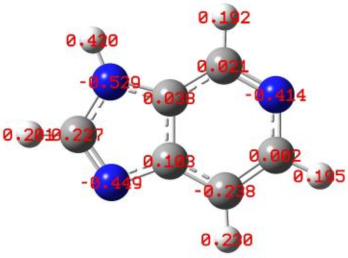
ske62
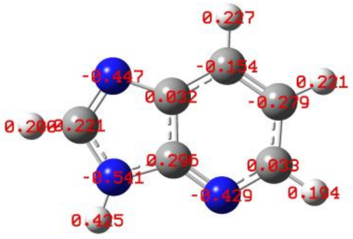
ske63
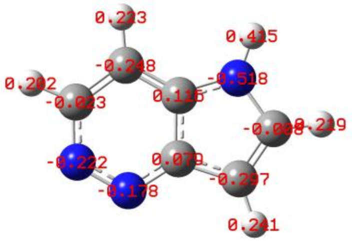
ske64
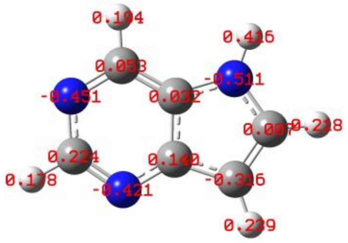
ske65
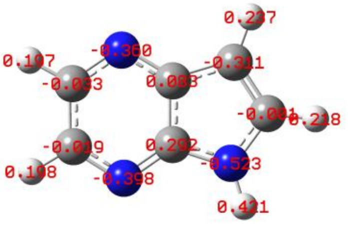
ske66
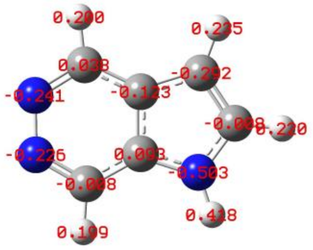
ske67
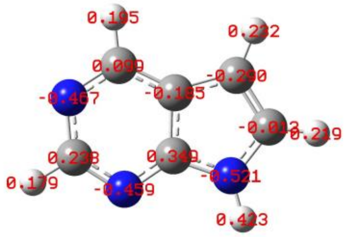
ske68
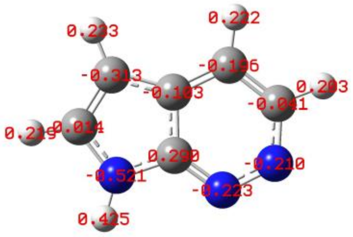
ske69
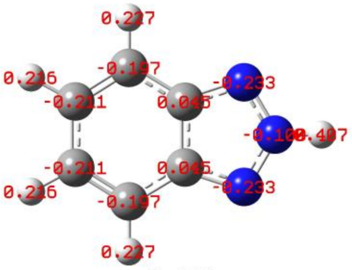
ske70
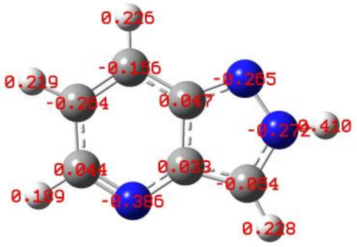
ske71
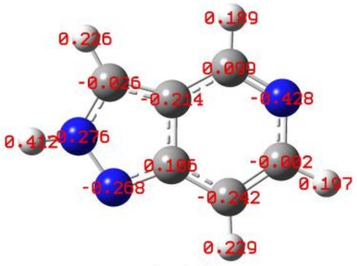
ske72
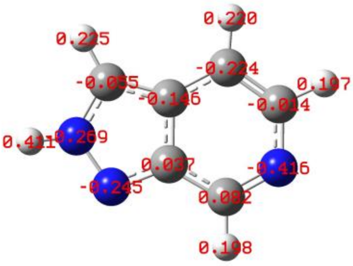
ske73
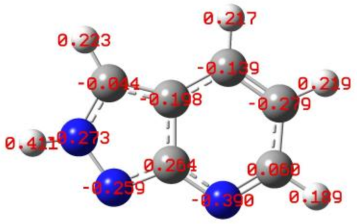
ske74
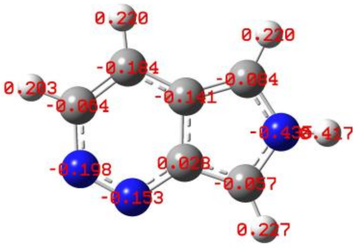
ske75

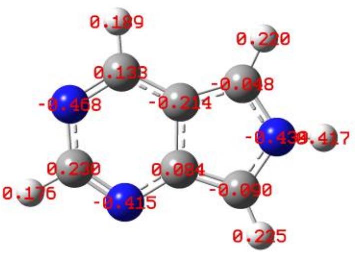

ske76

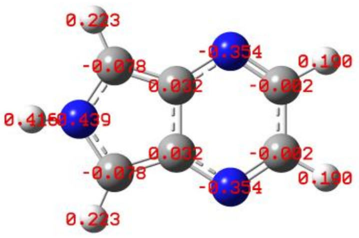

ske77

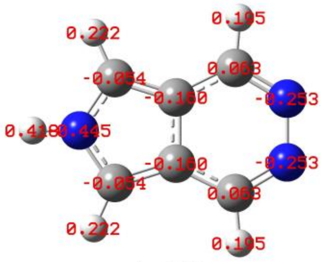

ske78

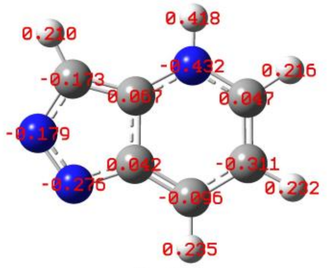

ske79

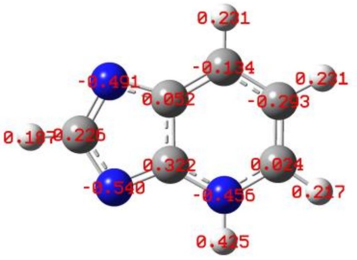

ske80

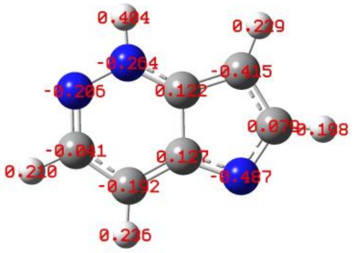

ske81

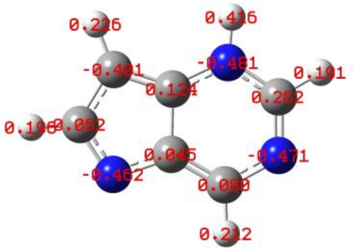

ske82

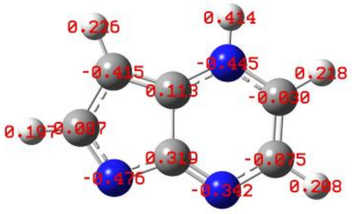

ske83

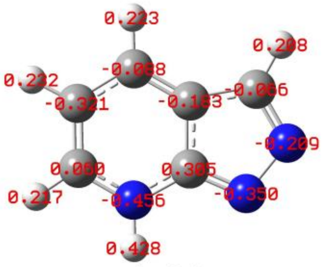

ske84

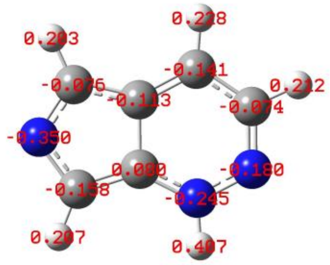

ske85

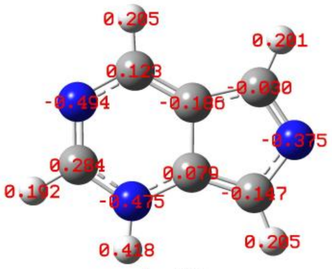

ske86

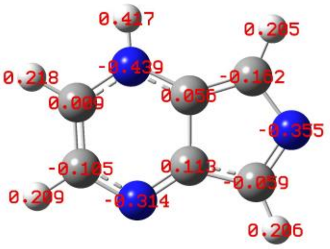

ske87

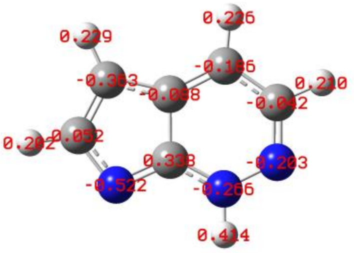

ske88

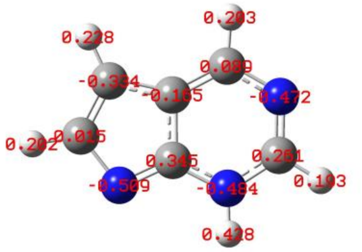

ske89

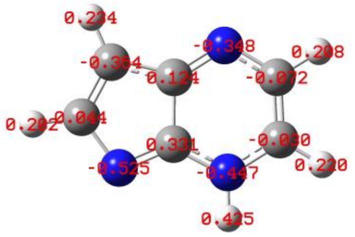

ske90

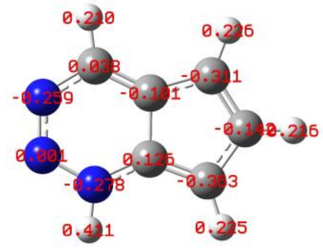

ske91

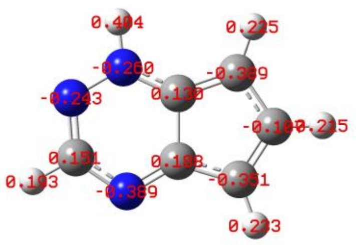

ske92

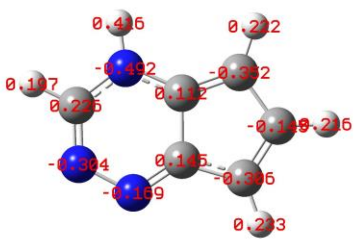

ske93

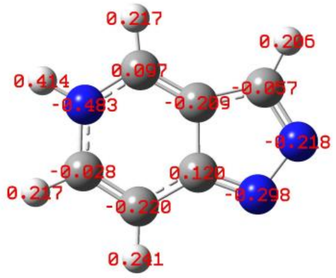

ske94

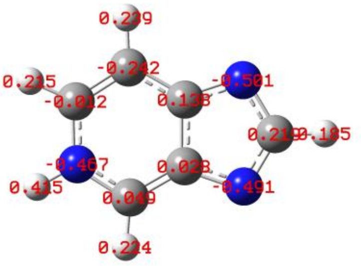

ske95

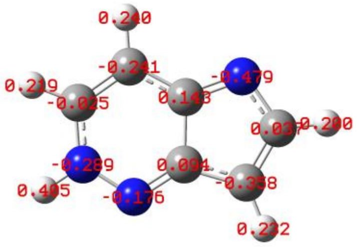

ske96

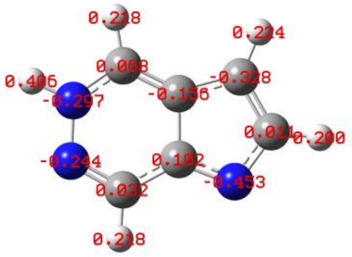

ske97

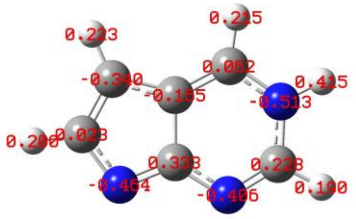

ske98

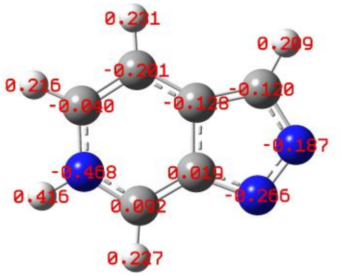

ske99

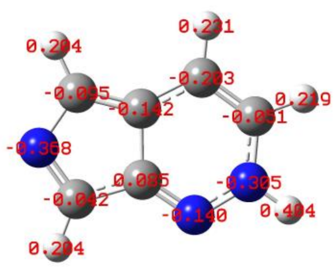

ske100

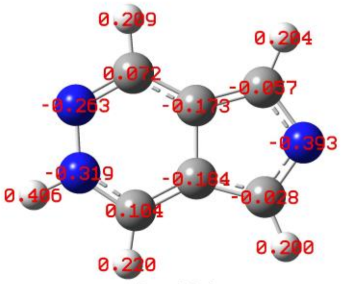

ske101

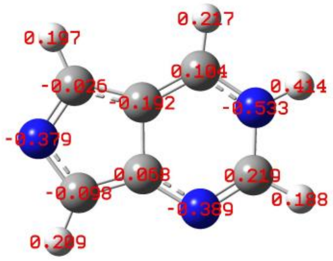

ske102

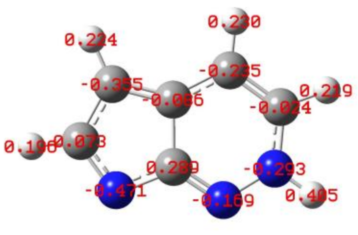

ske103

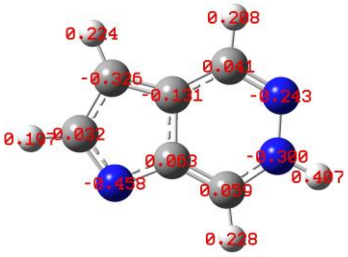

ske104

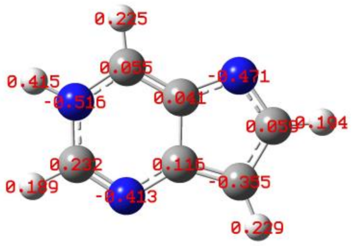

ske105

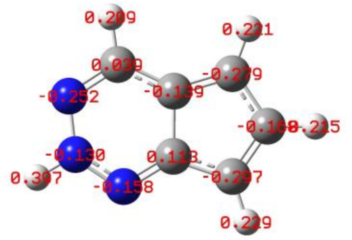

ske106

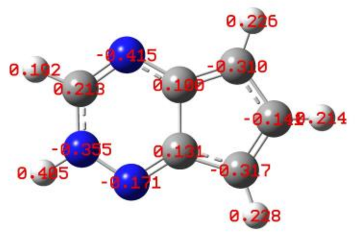

ske107

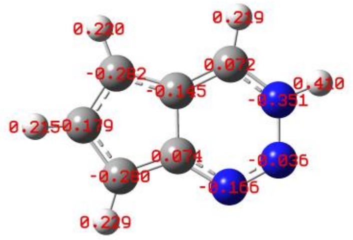

ske108

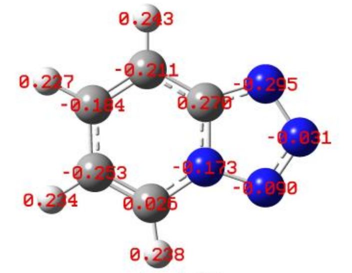

ske109

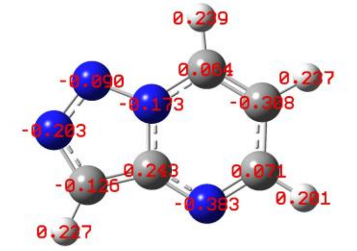

ske110

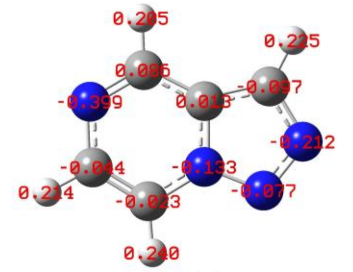

ske111

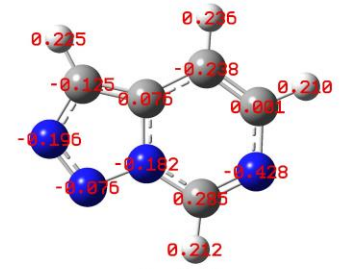

ske112

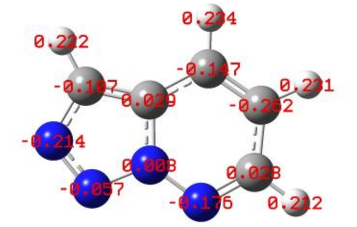

ske113

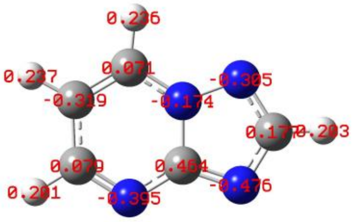

ske114

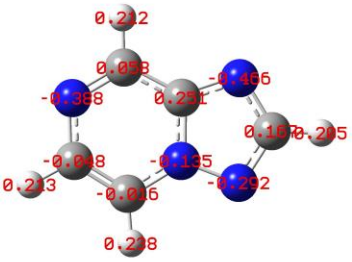

ske115

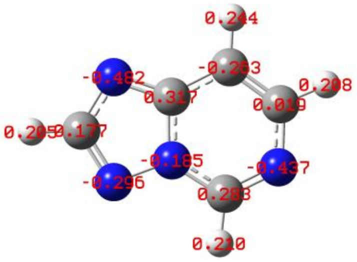

ske116

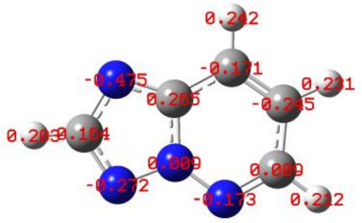

ske117

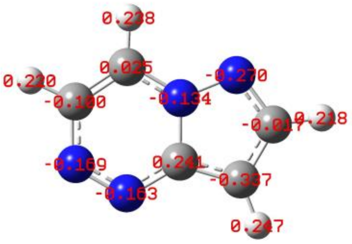

ske118

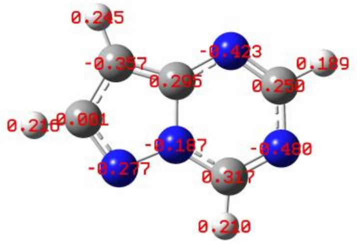

ske119

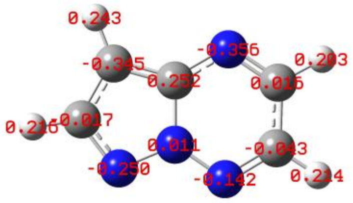

ske120

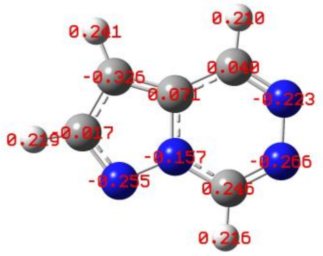

ske121

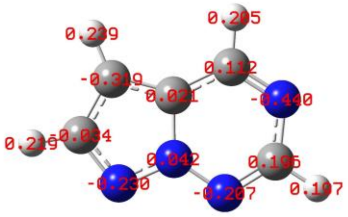

ske122

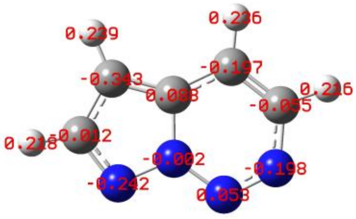

ske123

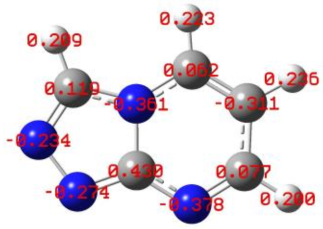

ske124

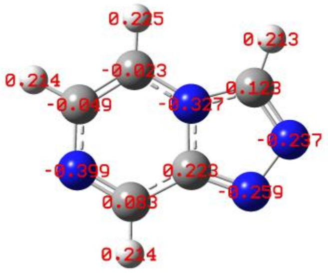

ske125

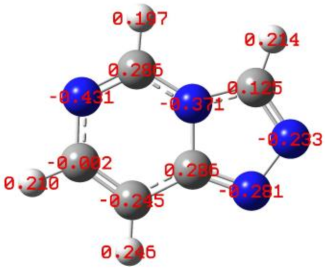

ske126

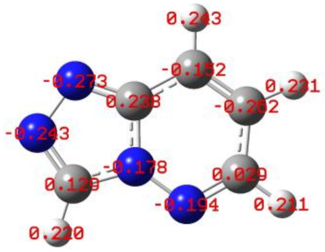

ske127

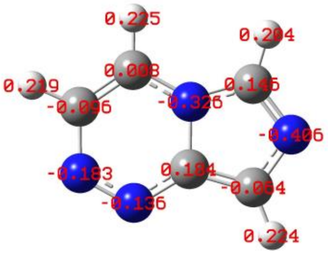

ske128

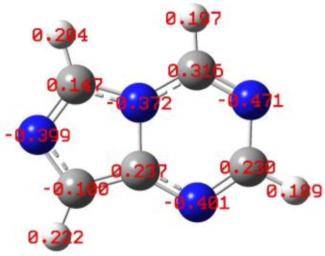

ske129

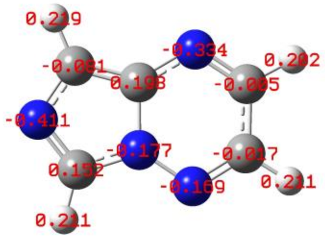

ske130

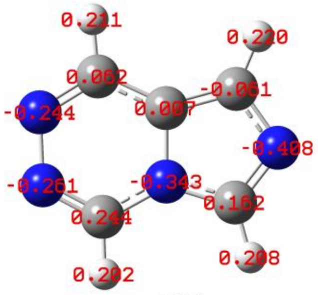

ske131

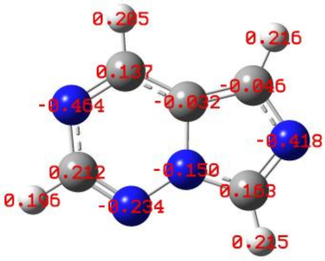

ske132

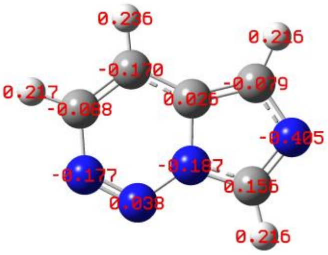

ske133

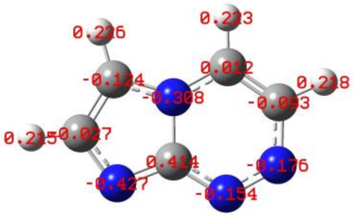

ske134

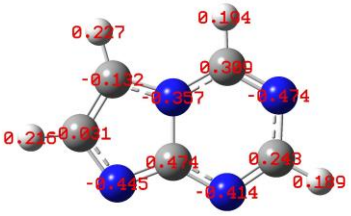

ske135

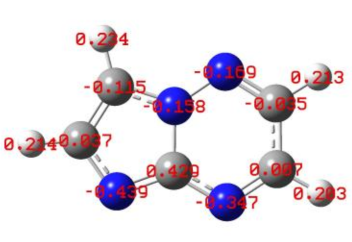

ske136

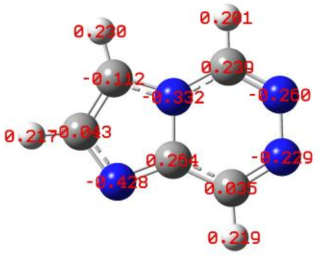

ske137

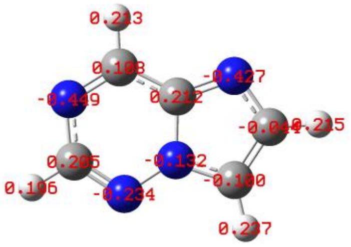

ske138

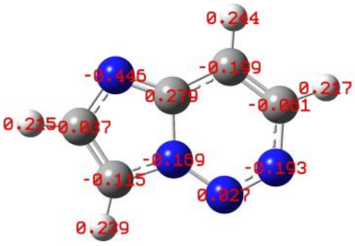

ske139

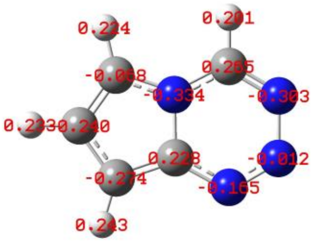

ske140

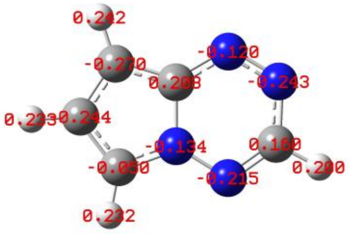

ske141

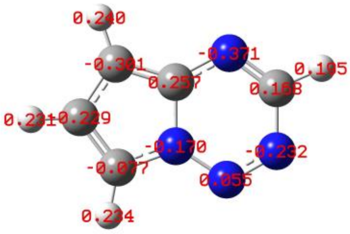

ske142

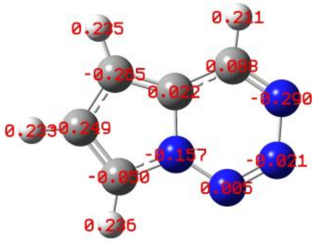

ske143

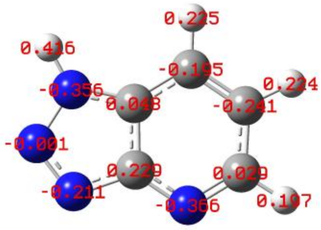

ske144

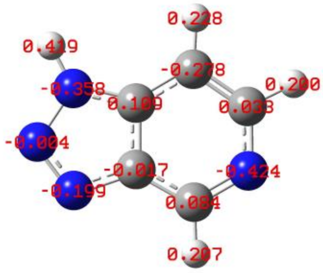

ske145

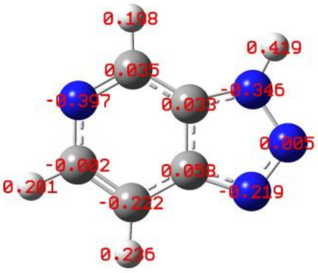

ske146

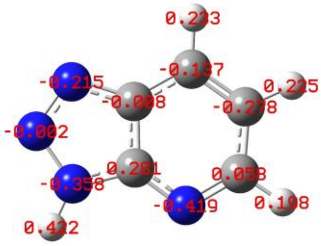

ske147

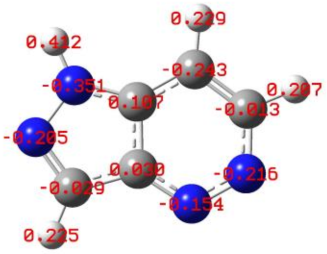

ske148

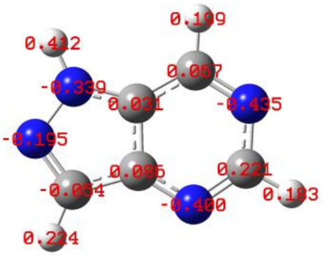

ske149

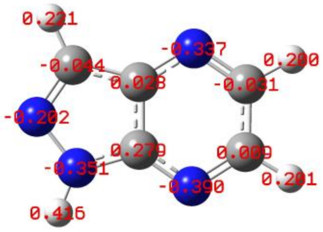

ske150

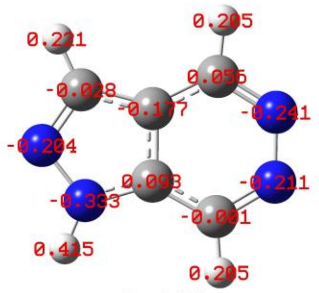

ske151

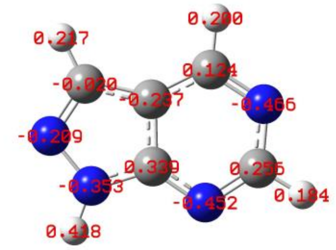

ske152

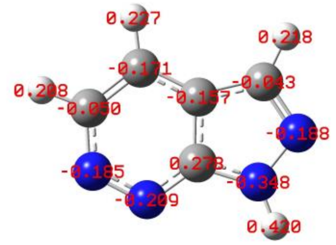

ske153

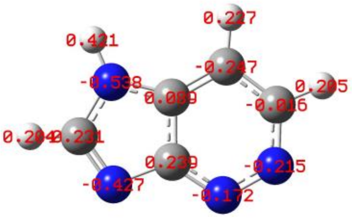

ske154

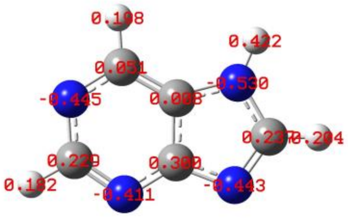

ske155

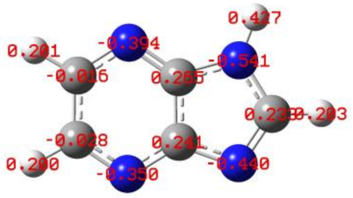

ske156

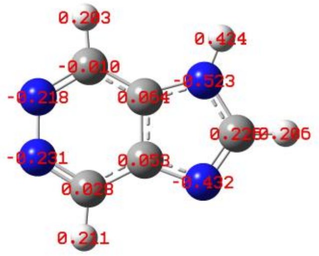

ske157

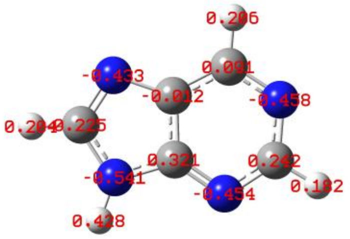

ske158

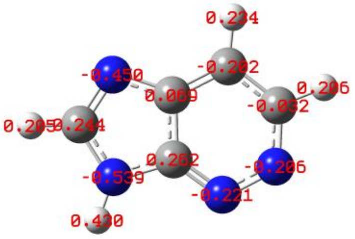

ske159

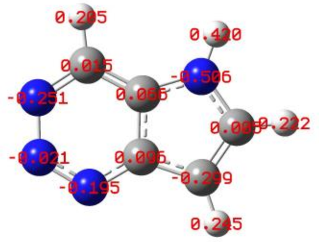

ske160

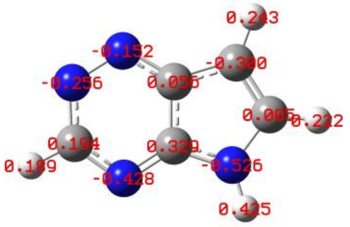

ske161

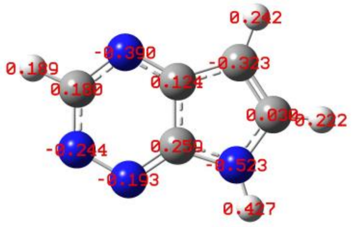

ske162

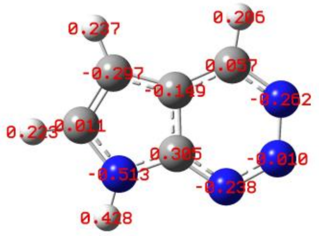

ske163

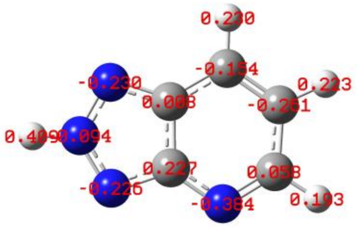

ske164

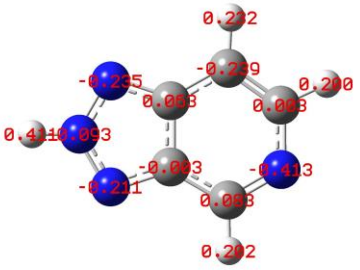

ske165

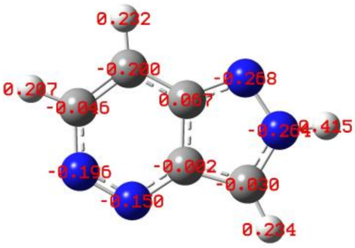
ske166

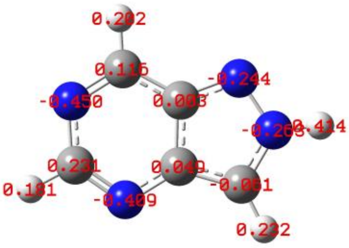
ske167

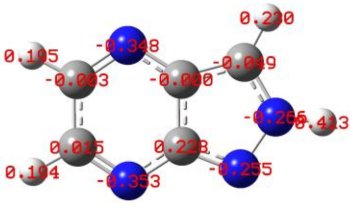
ske168

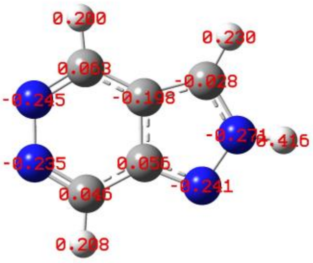
ske169

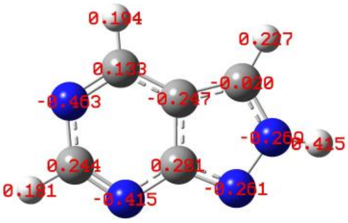
ske170

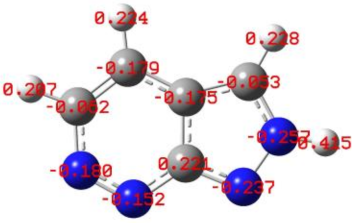
ske171

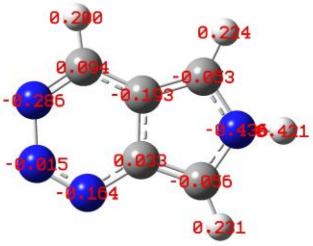
ske172

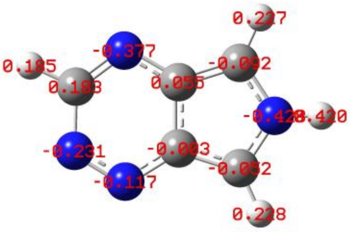
ske173

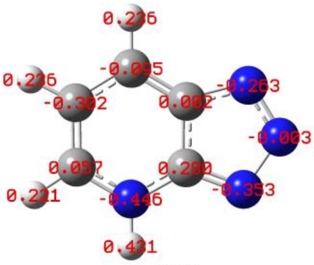
ske174

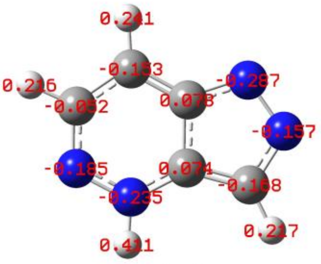
ske175

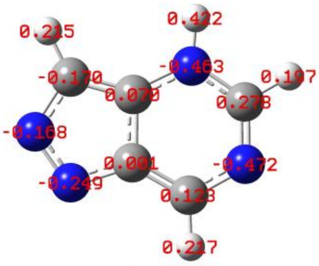
ske176

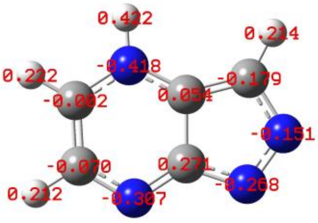
ske177

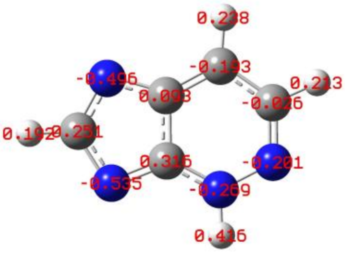
ske178

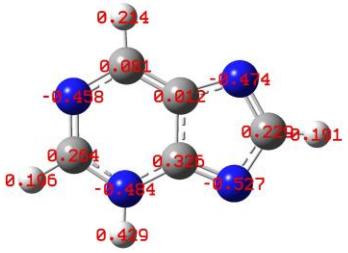
ske179

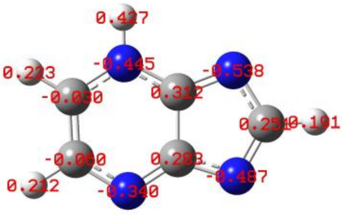
ske180

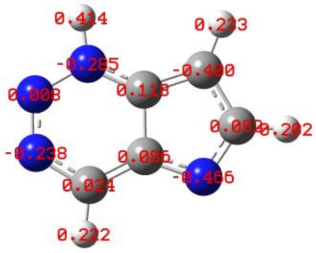

ske181

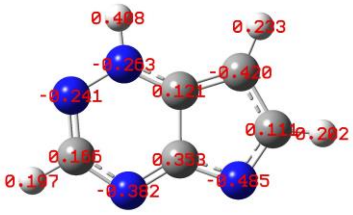

ske182

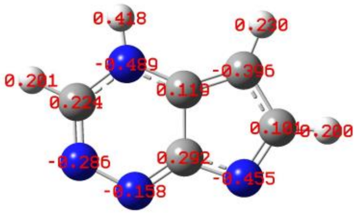

ske183

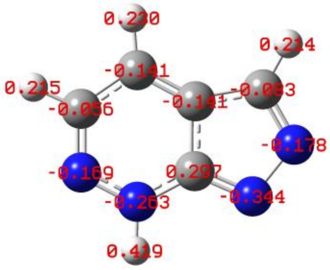

ske184

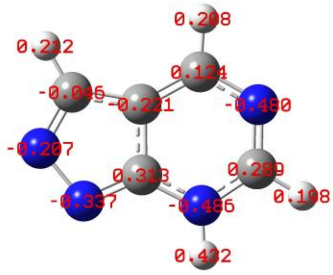

ske185

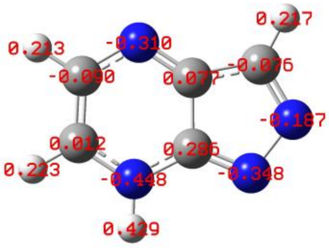

ske186

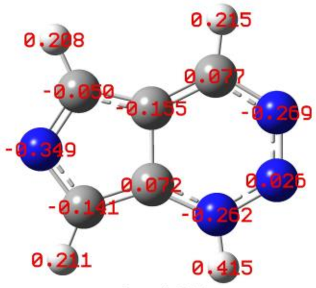

ske187

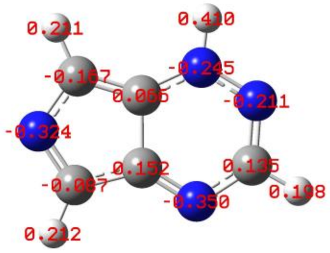

ske188

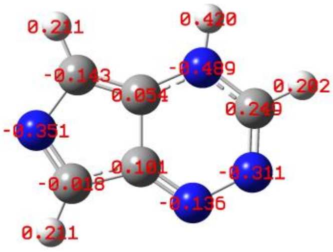

ske189

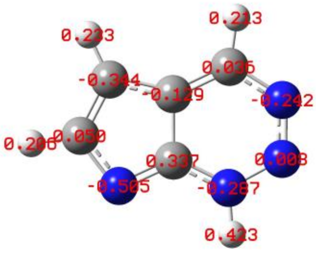

ske190

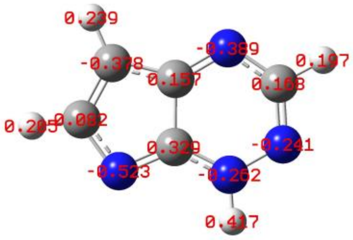

ske191

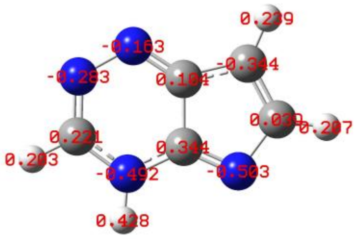

ske192

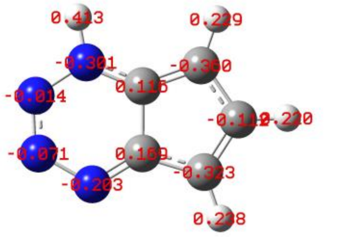

ske193

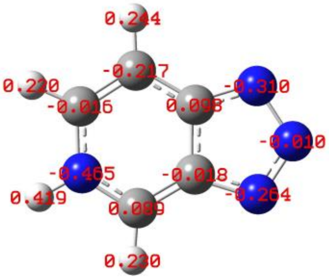

ske194

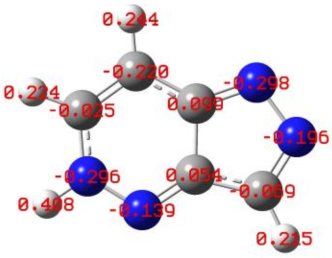

ske195

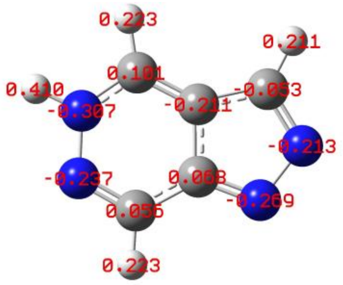
ske196

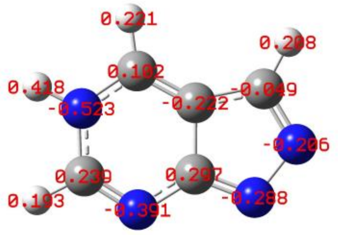
ske197

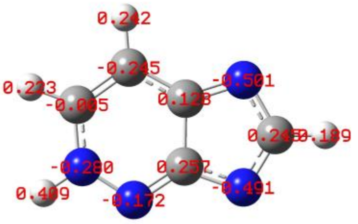
ske198

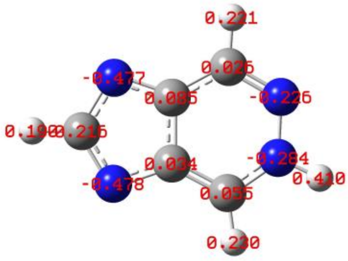
ske199

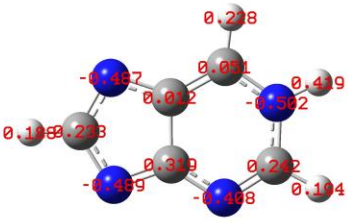
ske200

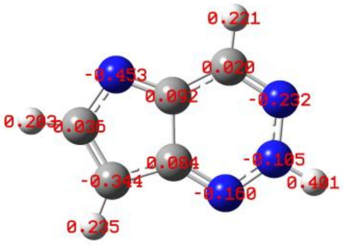
ske201

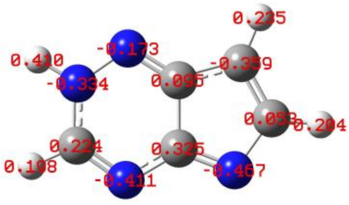
ske202

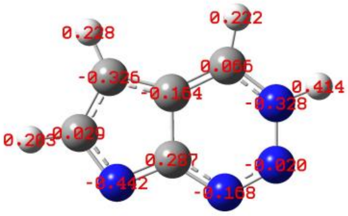
ske203

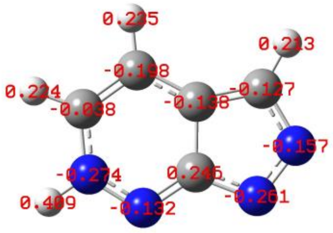
ske204

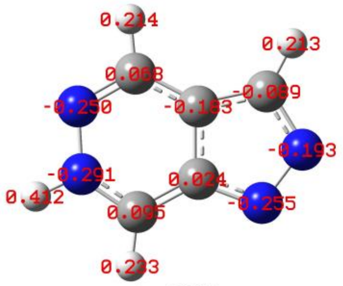
ske205

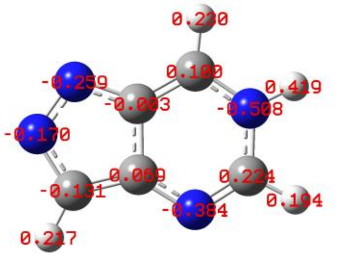
ske206

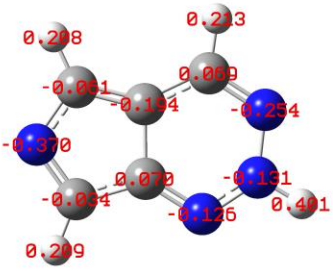
ske207

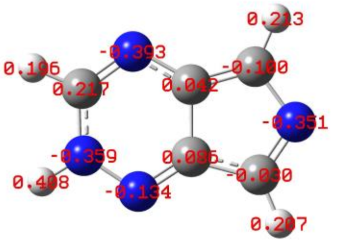
ske208

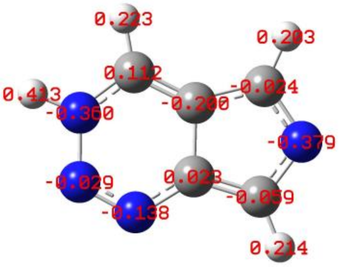
ske209

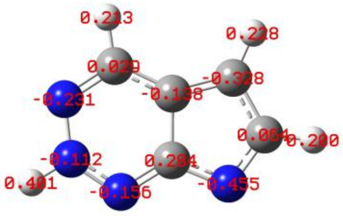
ske210

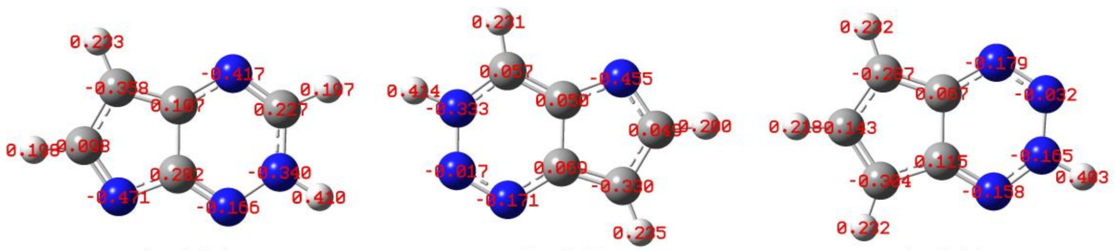

ske211 ske212 ske213

Figure S2 The geometric structure of optimized FR213 with NPA annotation.

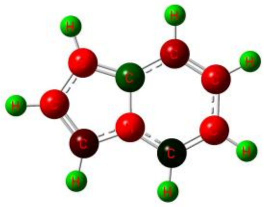
ske1
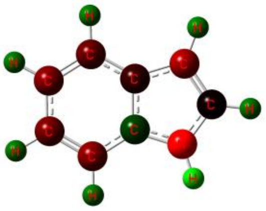
ske2
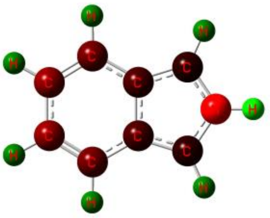
ske3
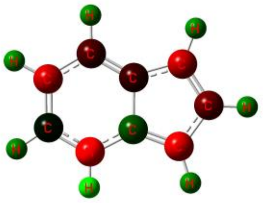
ske4
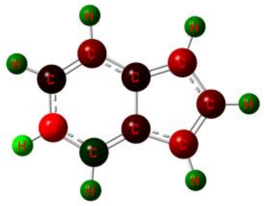
ske5
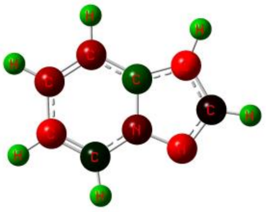
ske6
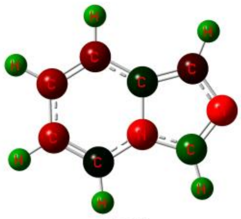
ske7
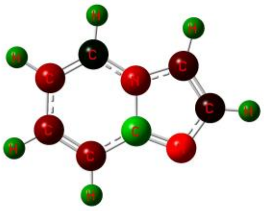
ske8
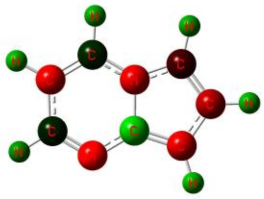
ske9
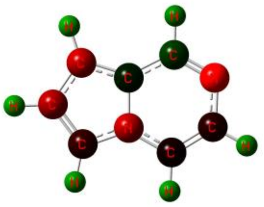
ske10
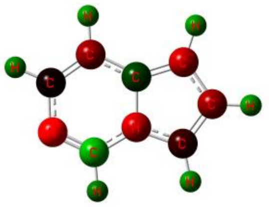
ske11
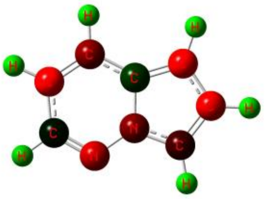
ske12
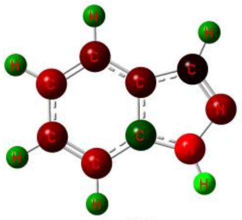
ske13
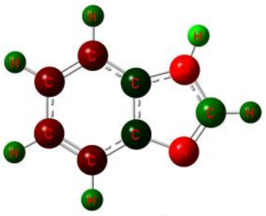
ske14
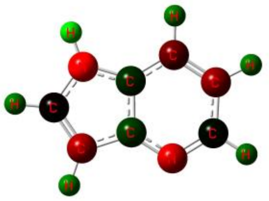
ske15
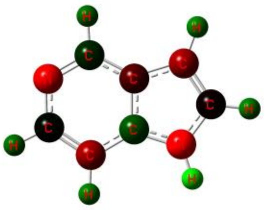
ske16
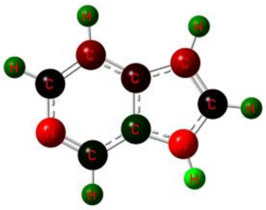
ske17
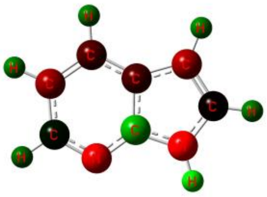
ske18
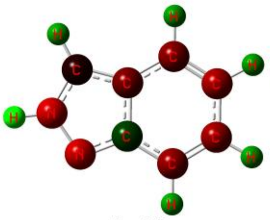
ske19
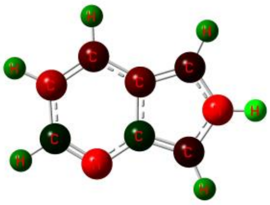
ske20
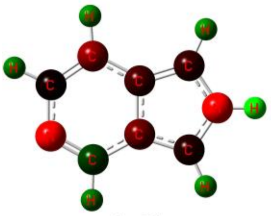
ske21
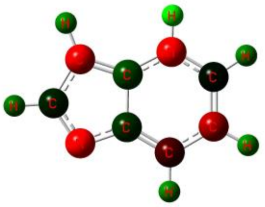
ske22
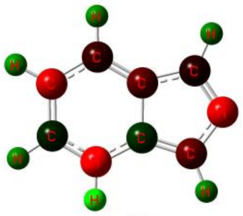
ske23
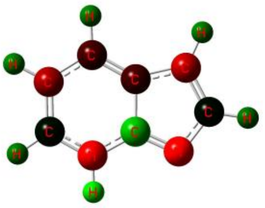
ske24

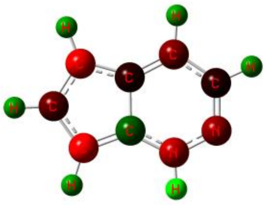

ske25

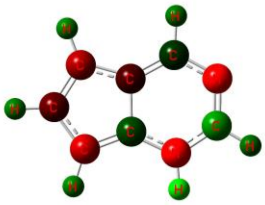

ske26

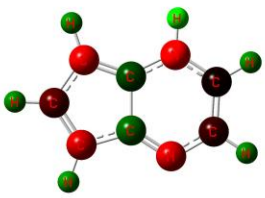

ske27

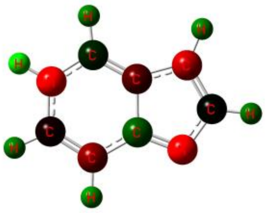

ske28

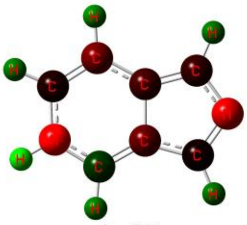

ske29

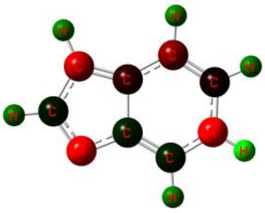

ske30

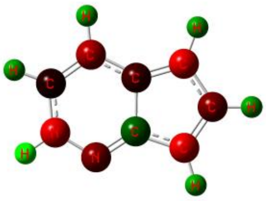

ske31

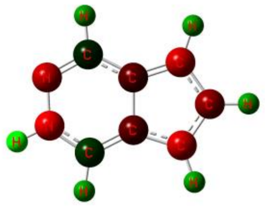

ske32

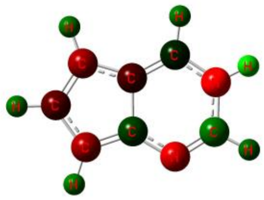

ske33

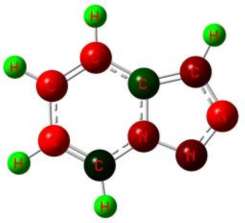

ske34

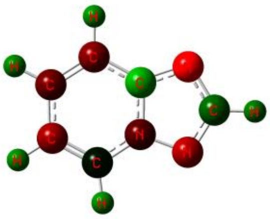

ske35

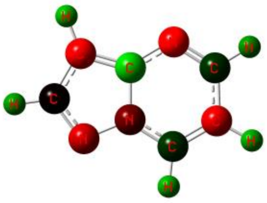

ske36

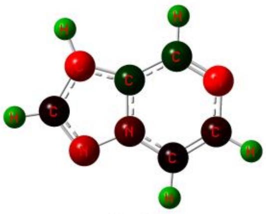

ske37

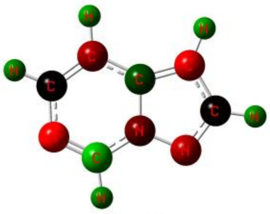

ske38

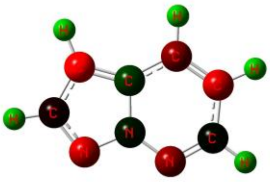

ske39

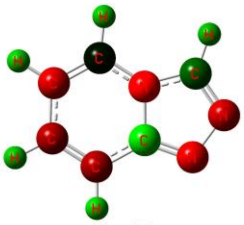

ske40

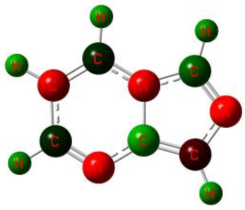

ske41

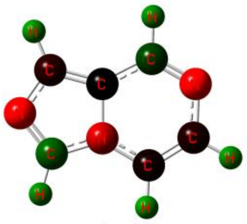

ske42

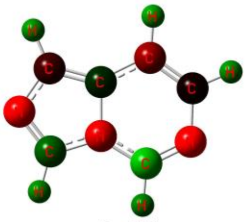

ske43

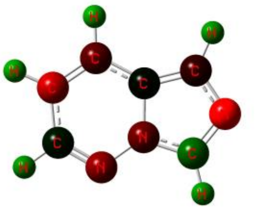

ske44

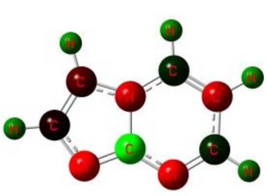

ske45

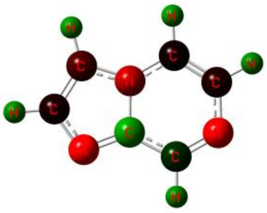

ske46

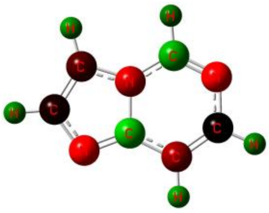

ske47

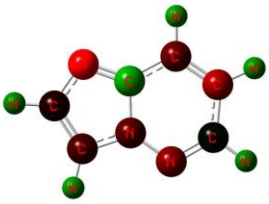

ske48

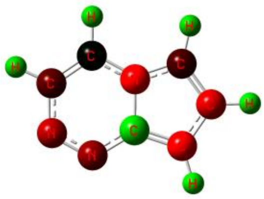
ske49

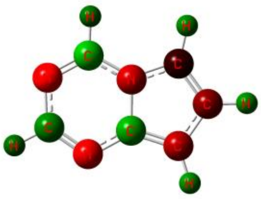
ske50

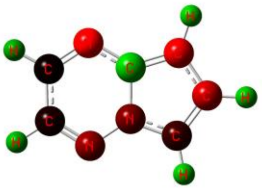
ske51

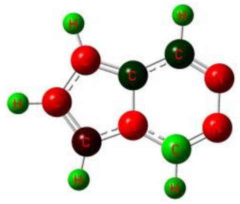
ske52

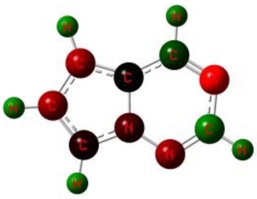
ske53

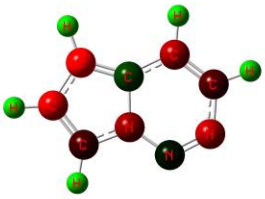
ske54

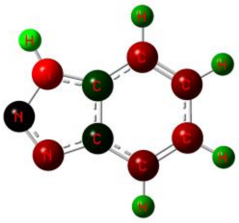
ske55

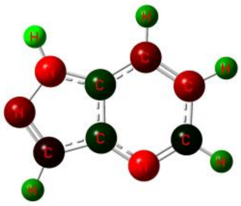
ske56

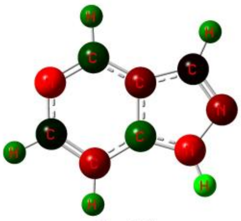
ske57

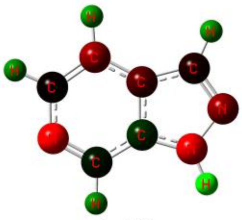
ske58

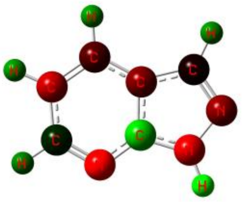
ske59

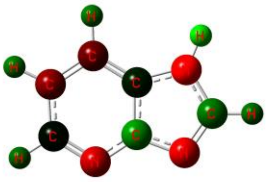
ske60

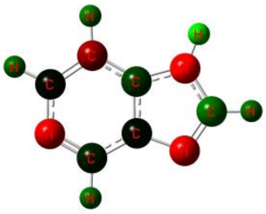
ske61

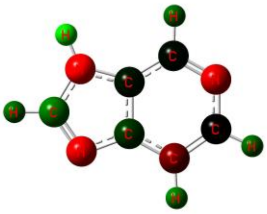
ske62

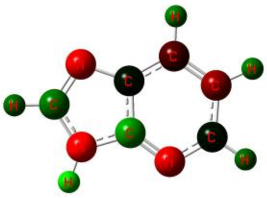
ske63

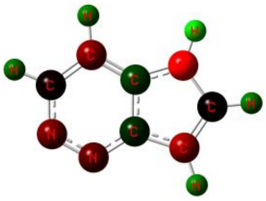
ske64

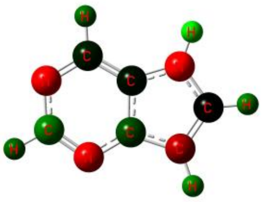
ske65

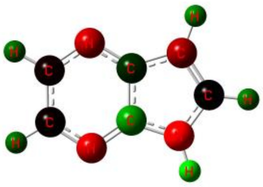
ske66

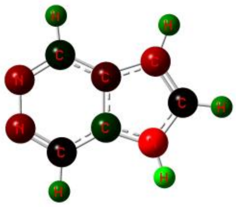
ske67

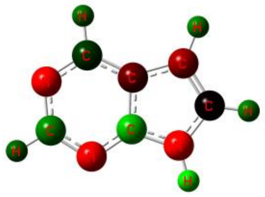
ske68

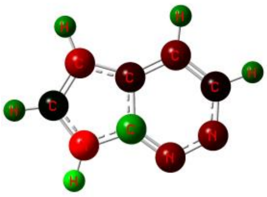
ske69

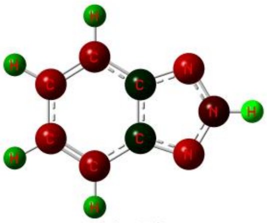
ske70

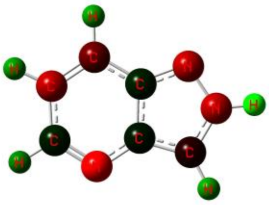
ske71

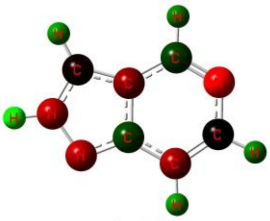
ske72

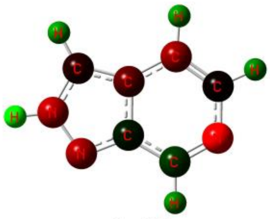

ske73

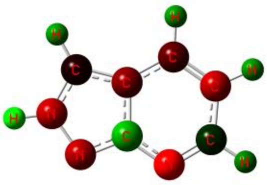

ske74

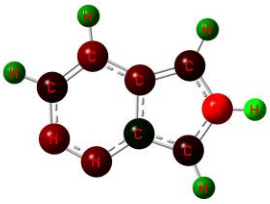

ske75

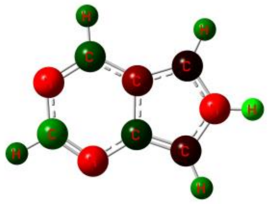

ske76

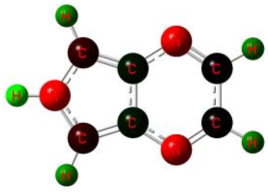

ske77

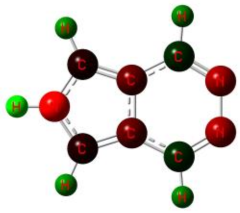

ske78

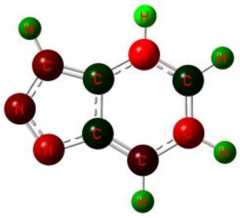

ske79

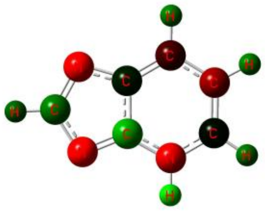

ske80

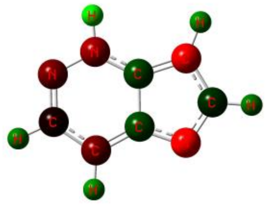

ske81

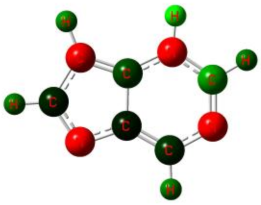

ske82

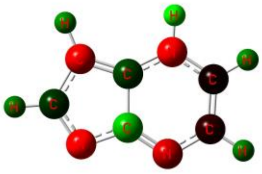

ske83

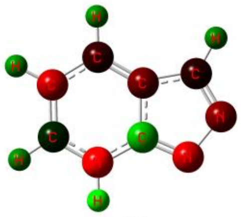

ske84

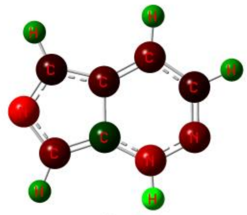

ske85

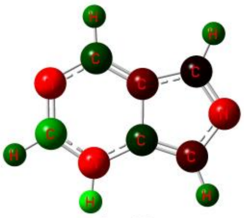

ske86

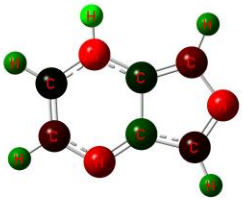

ske87

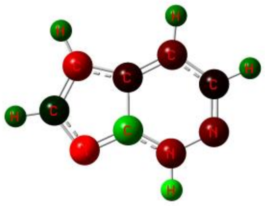

ske88

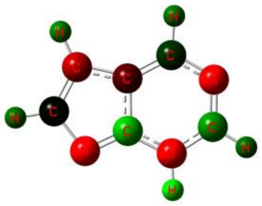

ske89

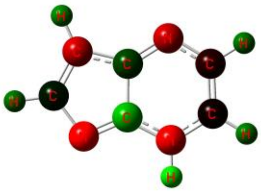

ske90

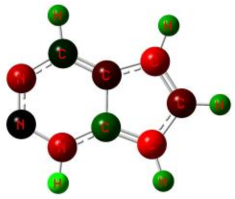

ske91

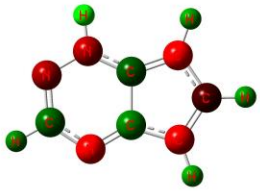

ske92

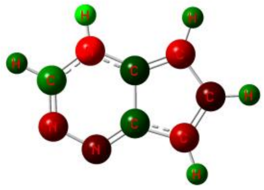

ske93

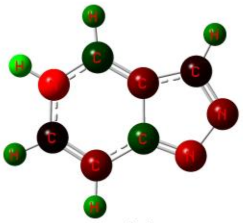

ske94

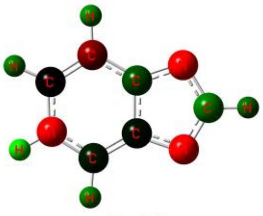

ske95

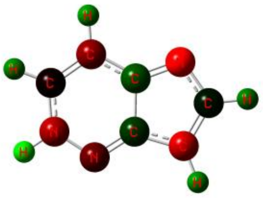

ske96

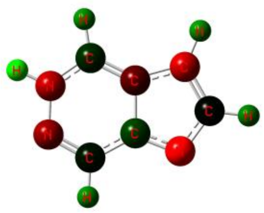 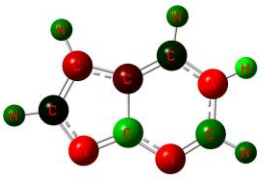 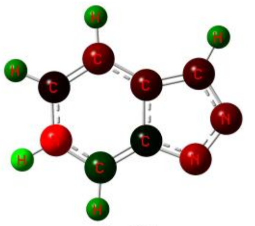 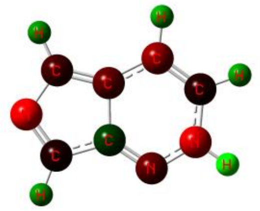

ske97 ske98 ske99 ske100

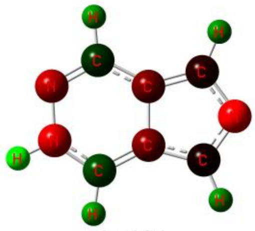 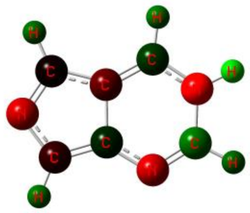 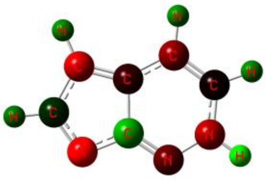 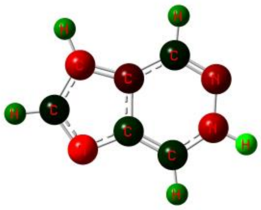

ske101 ske102 ske103 ske104

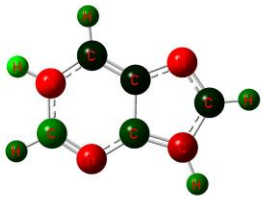 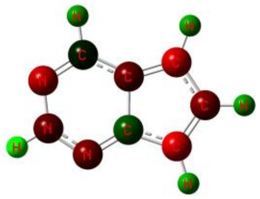 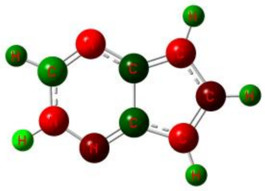 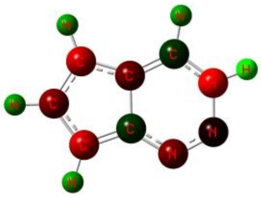

ske105 ske106 ske107 ske108

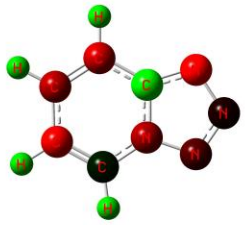 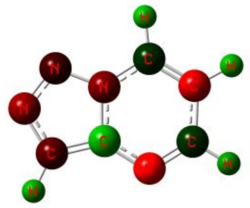 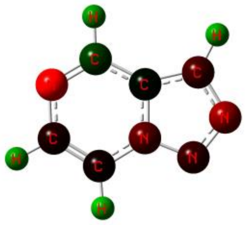 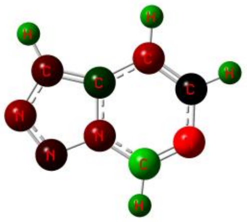

ske109 ske110 ske111 ske112

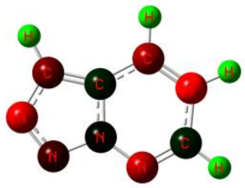 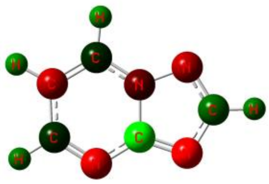 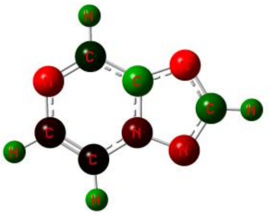 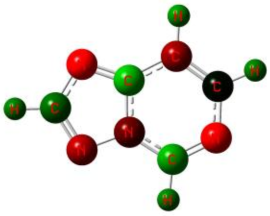

ske113 ske114 ske115 ske116

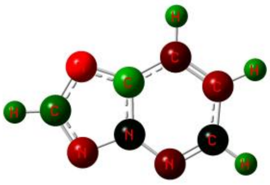 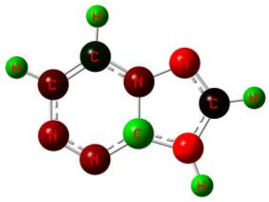 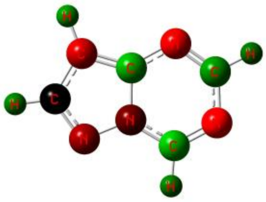 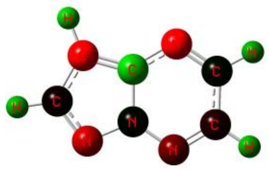

ske117 ske118 ske119 ske120

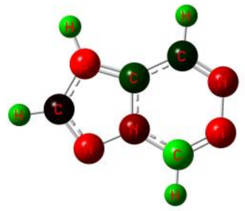
ske121

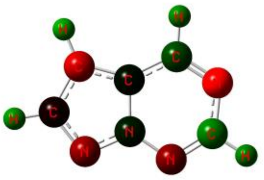
ske122

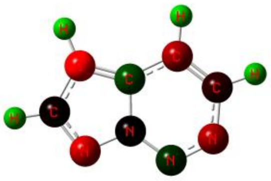
ske123

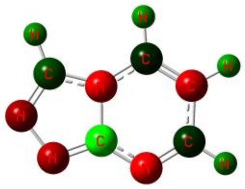
ske124

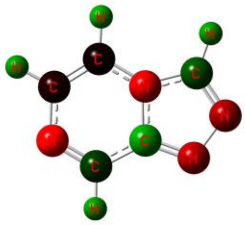
ske125

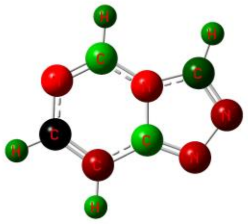
ske126

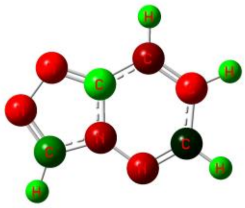
ske127

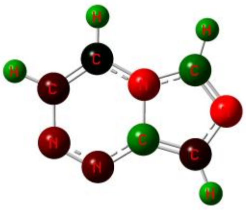
ske128

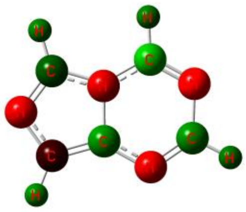
ske129

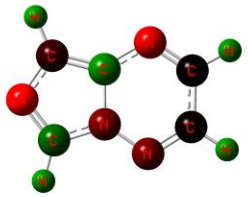
ske130

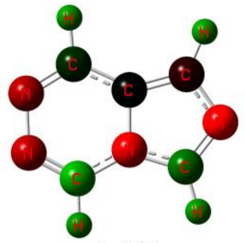
ske131

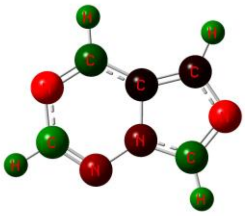
ske132

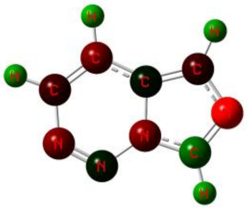
ske133

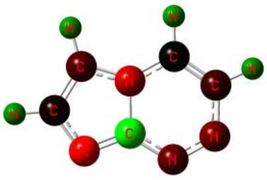
ske134

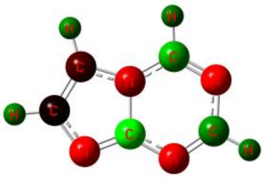
ske135

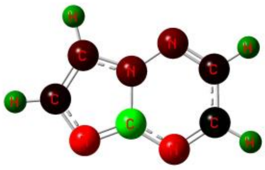
ske136

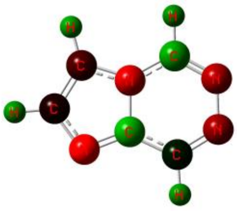
ske137

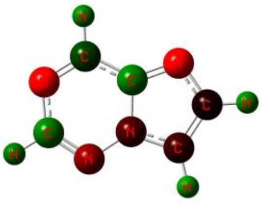
ske138

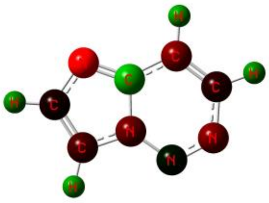
ske139

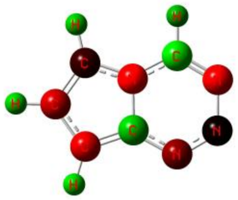
ske140

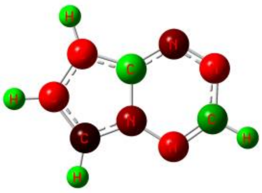
ske141

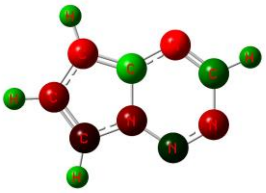
ske142

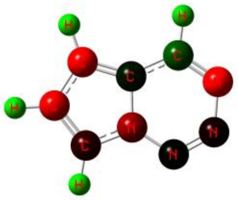
ske143

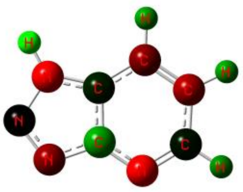
ske144

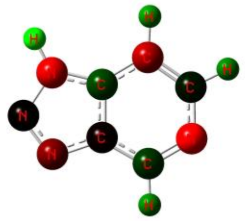
ske145

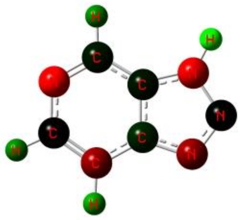
ske146

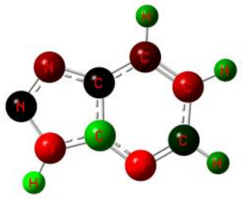
ske147

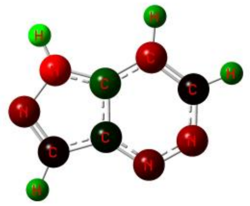
ske148

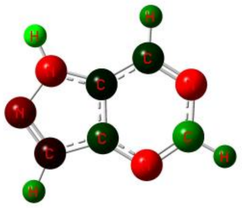
ske149

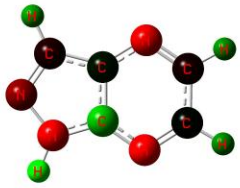
ske150

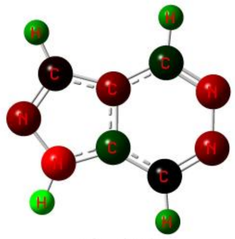
ske151

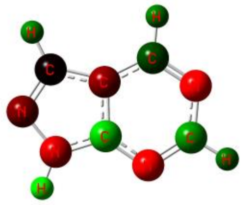
ske152

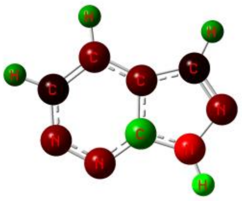
ske153

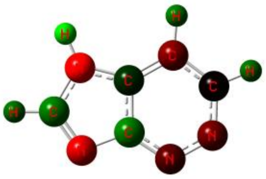
ske154

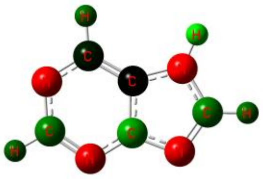
ske155

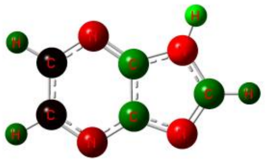
ske156

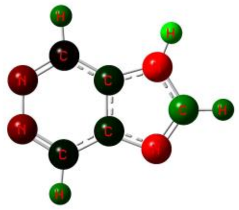
ske157

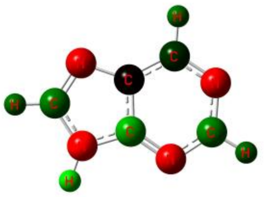
ske158

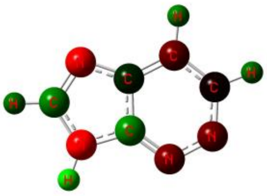
ske159

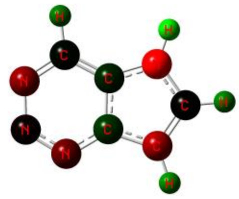
ske160

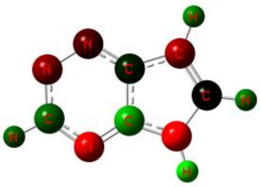
ske161

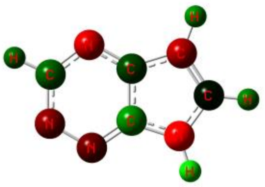
ske162

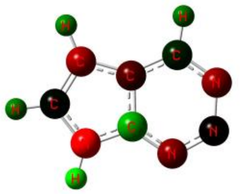
ske163

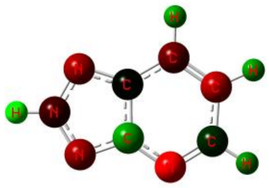
ske164

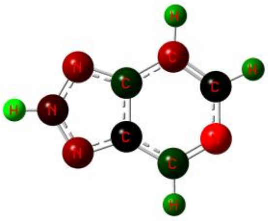
ske165

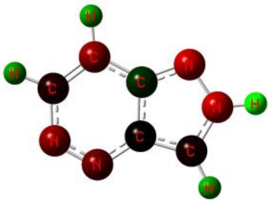
ske166

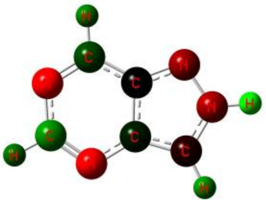
ske167

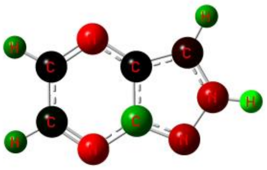
ske168

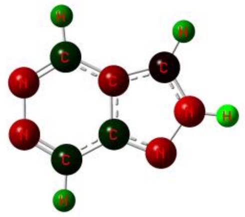
ske169

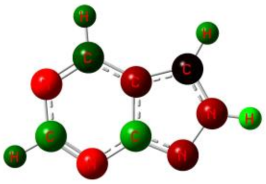
ske170

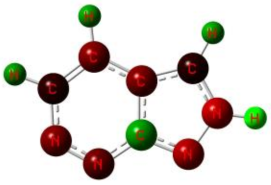
ske171

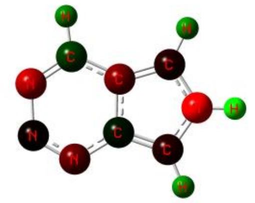
ske172

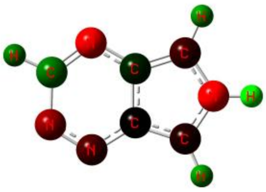
ske173

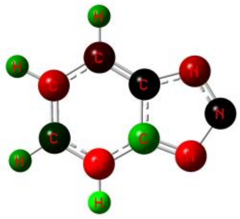
ske174

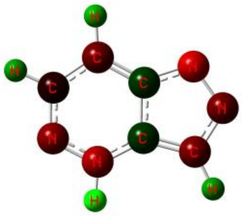
ske175

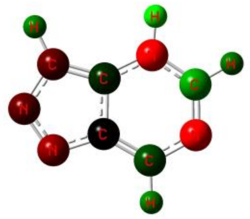
ske176

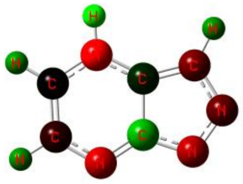
ske177

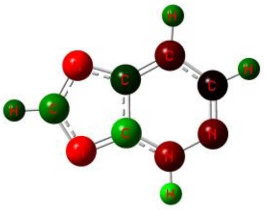
ske178

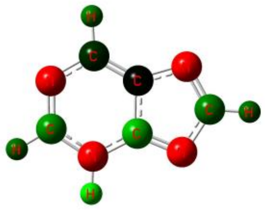
ske179

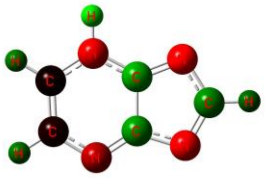
ske180

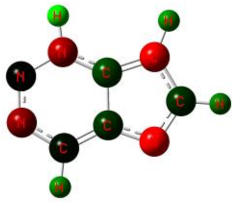
ske181

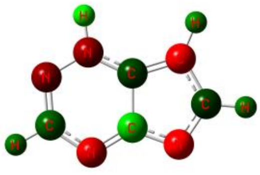
ske182

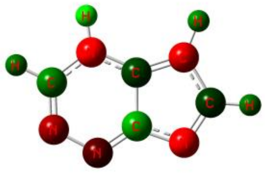
ske183

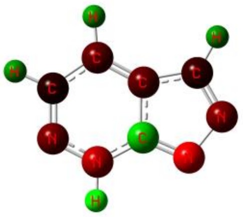
ske184

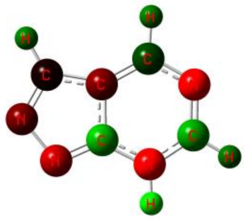
ske185

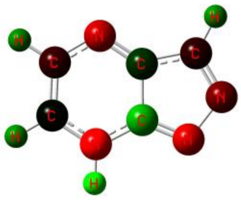
ske186

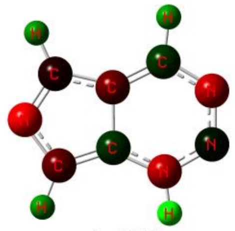
ske187

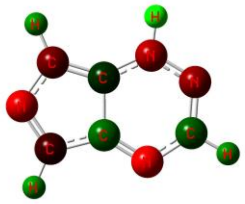
ske188

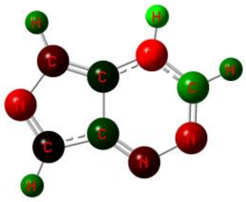
ske189

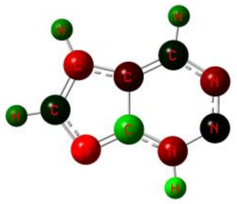
ske190

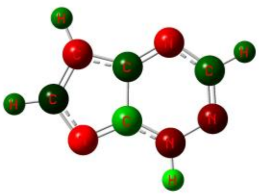
ske191

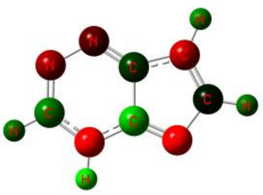
ske192

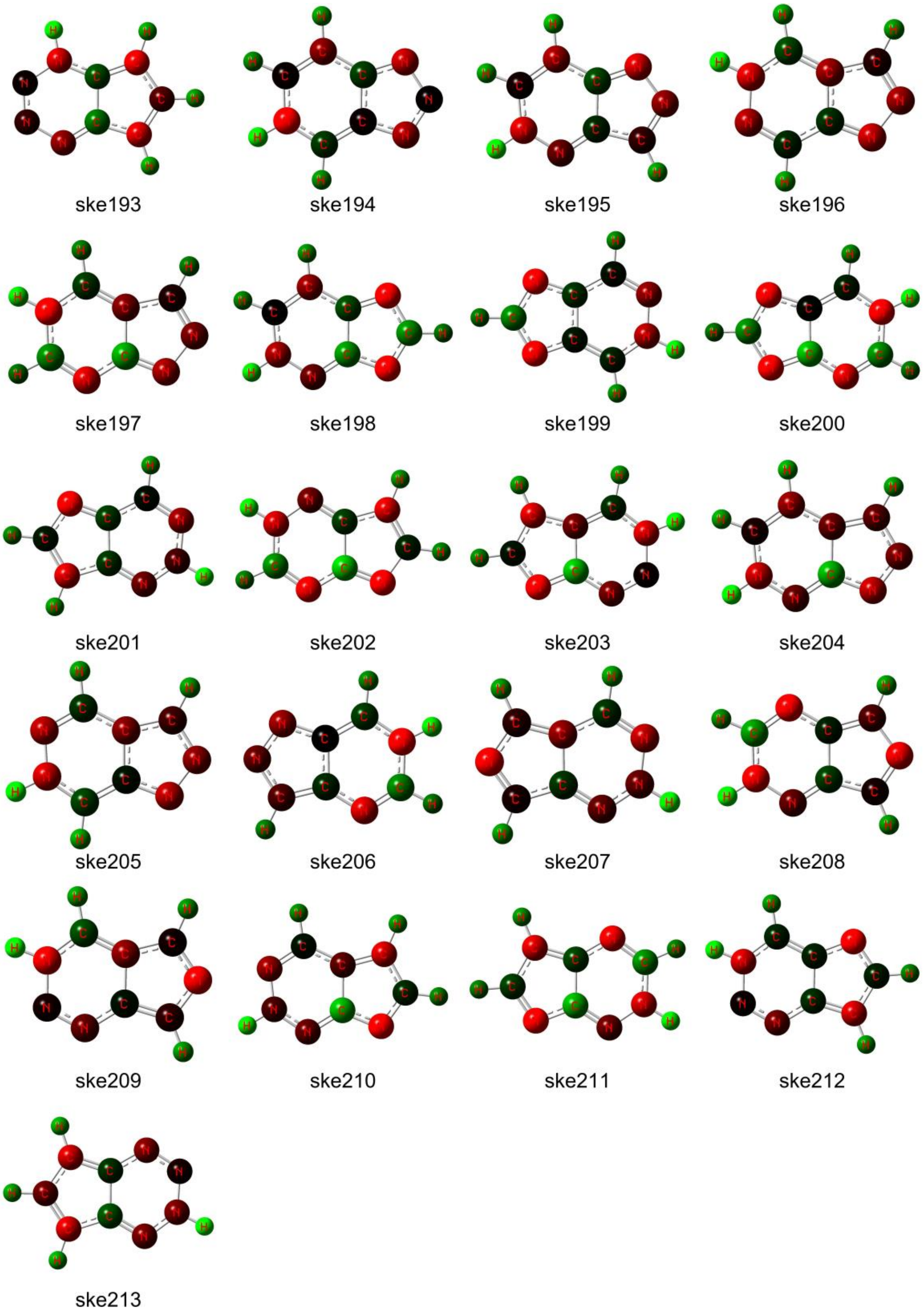

Figure S3 NPA charge color mapping. Red represents negative values, and green represents positive values.

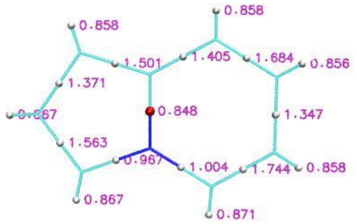

ske1

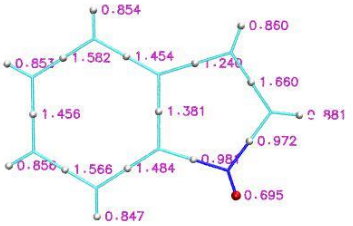

ske2

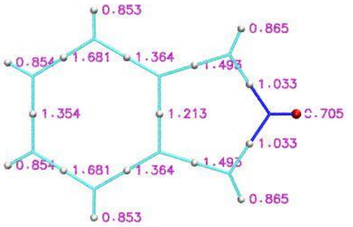

ske3

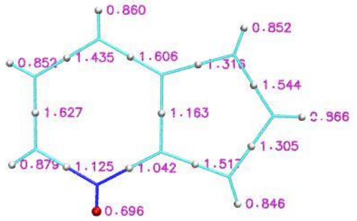

ske4

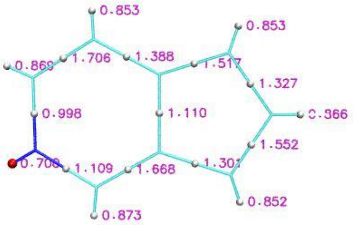

ske5

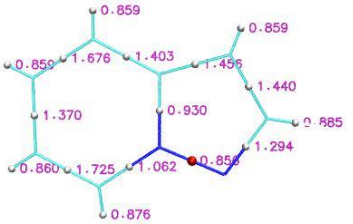

ske6

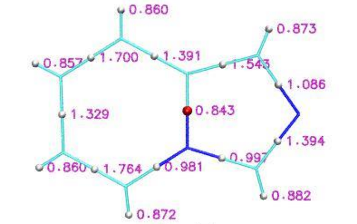

ske7

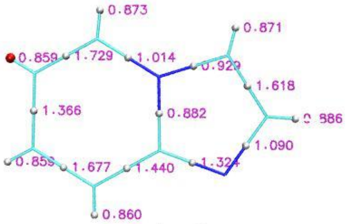

ske8

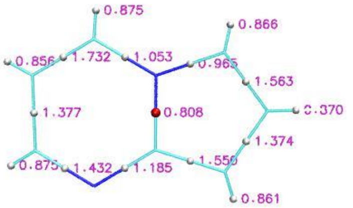

ske9

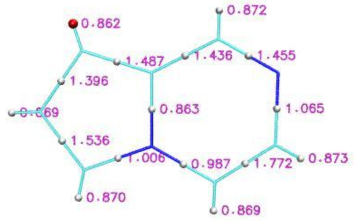

ske10

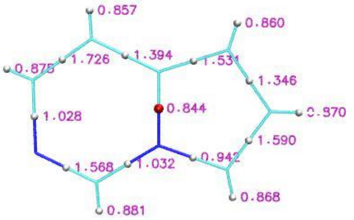

ske11

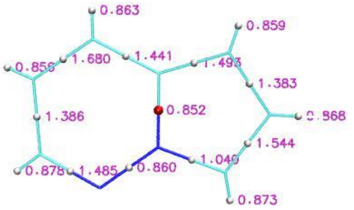

ske12

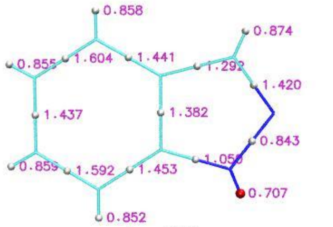

ske13

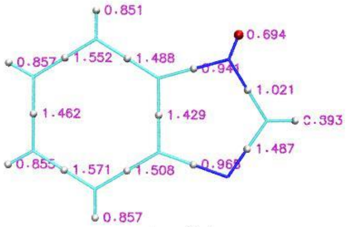

ske14

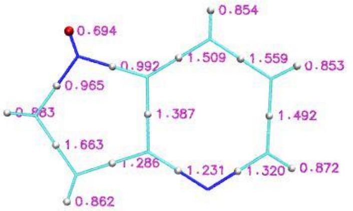

ske15

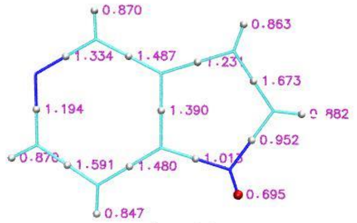

ske16

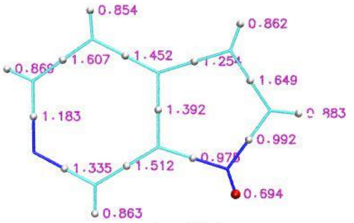

ske17

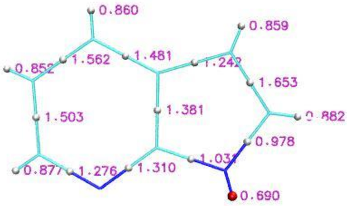

ske18

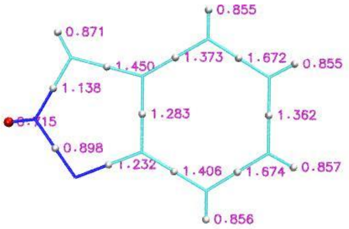

ske19

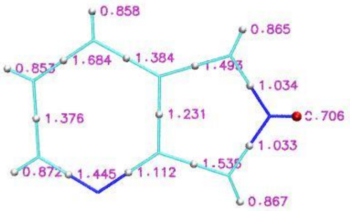

ske20

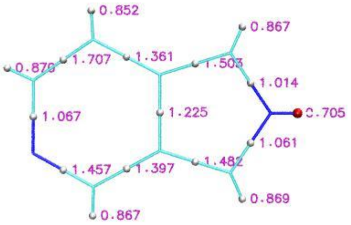

ske21

ske22

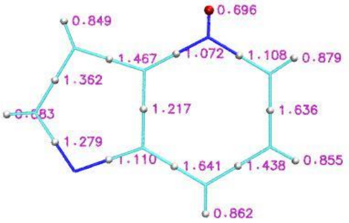

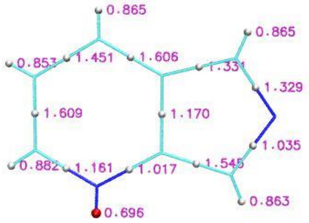

ske23

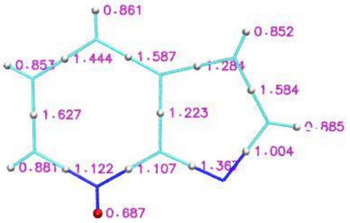

ske24

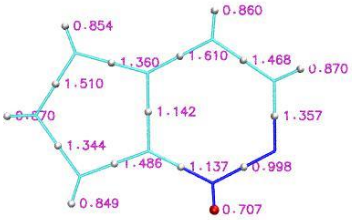

ske25

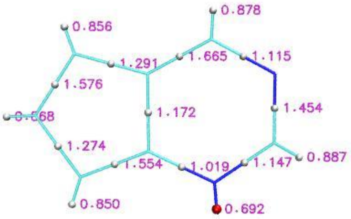

ske26

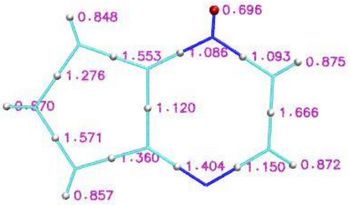

ske27

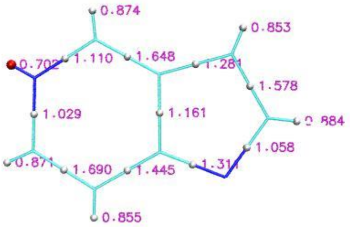

ske28

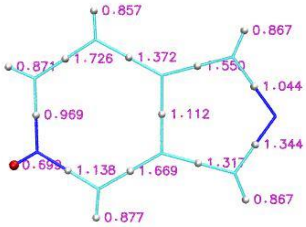

ske29

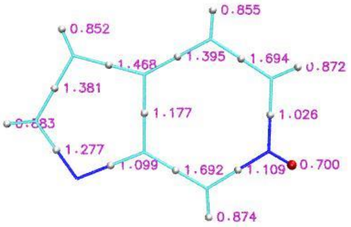

ske30

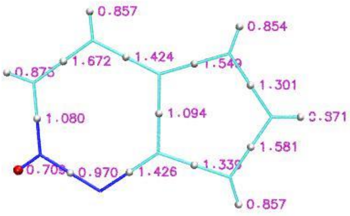

ske31

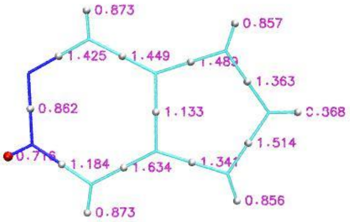

ske32

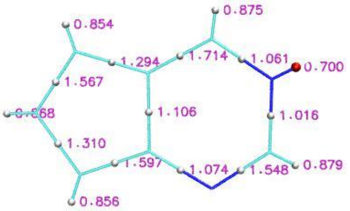

ske33

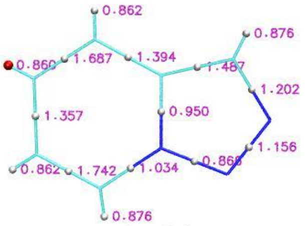

ske34

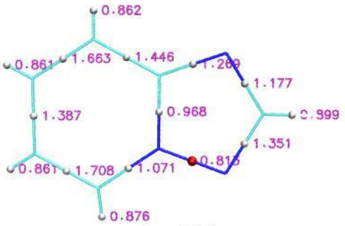

ske35

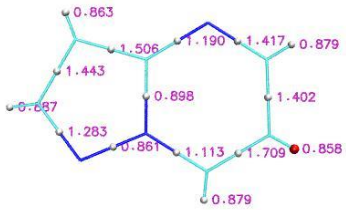

ske36

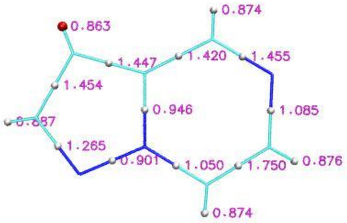

ske37

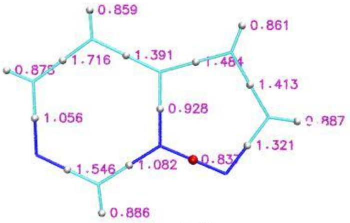

ske38

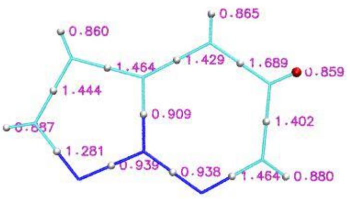

ske39

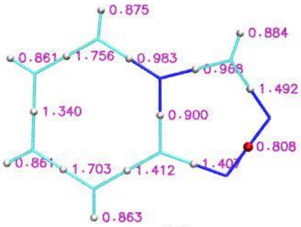

ske40

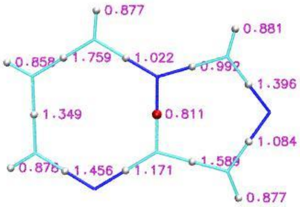

ske41

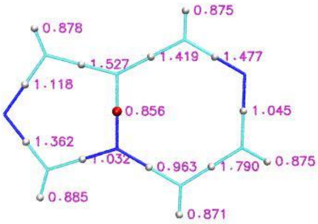

ske42

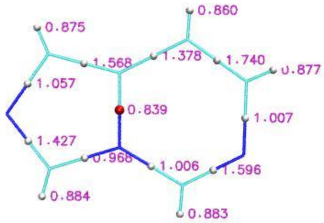

ske43

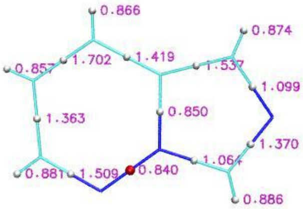

ske44

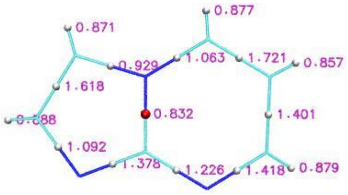

ske45

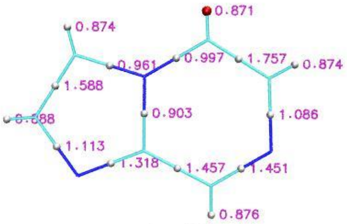

ske46

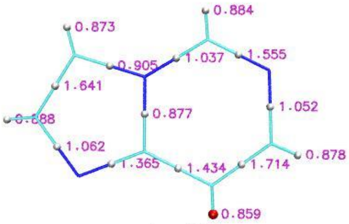

ske47

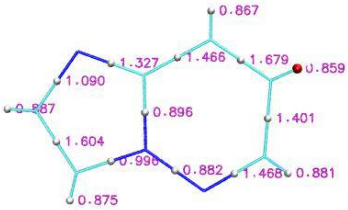

ske48

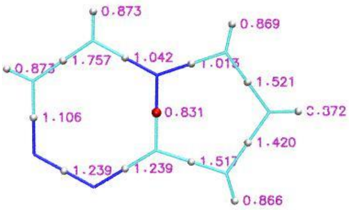

ske49

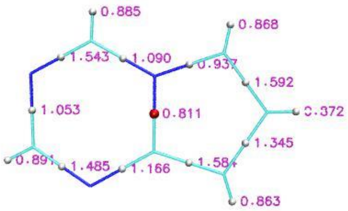

ske50

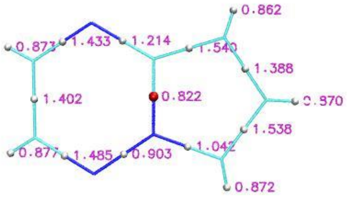

ske51

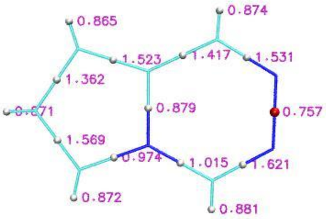

ske52

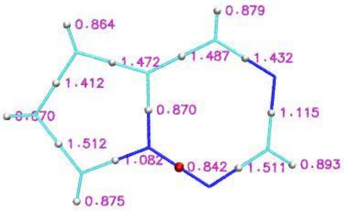

ske53

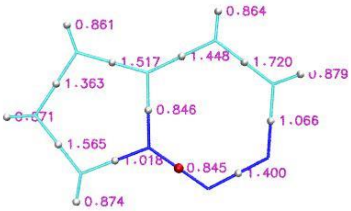

ske54

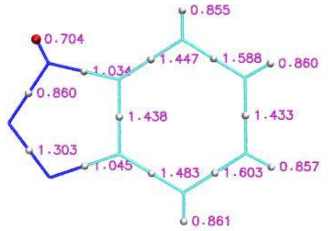

ske55

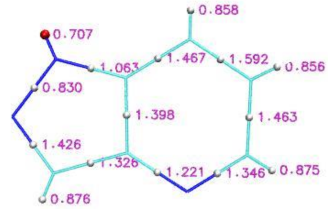

ske56

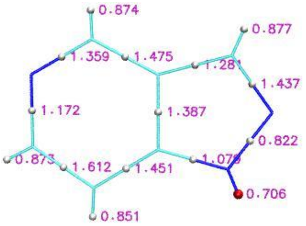

ske57

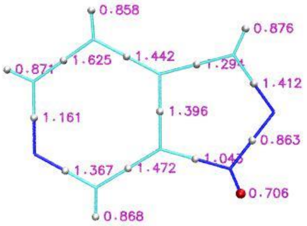

ske58

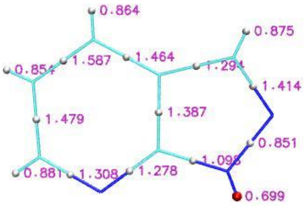

ske59

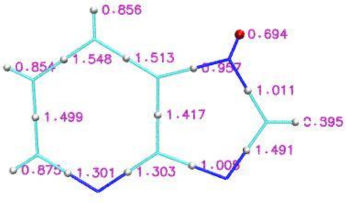

ske60

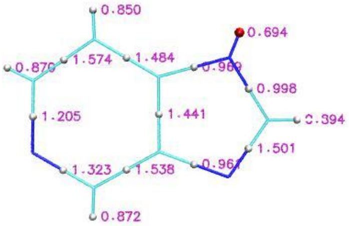

ske61

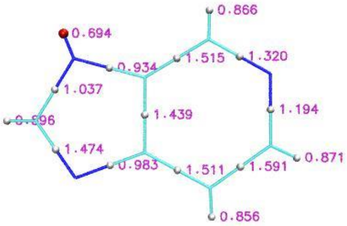

ske62

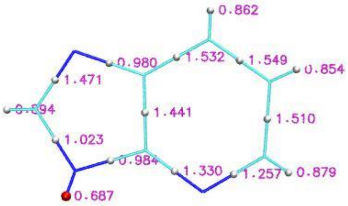

ske63

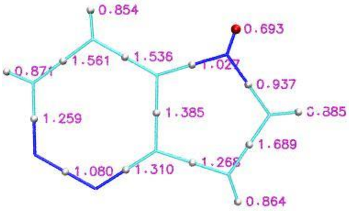

ske64

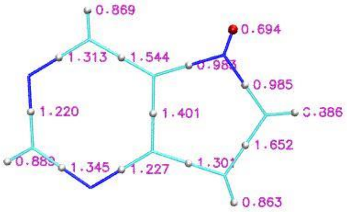

ske65

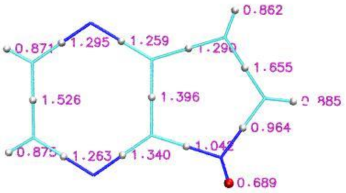

ske66

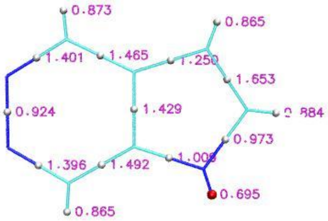

ske67

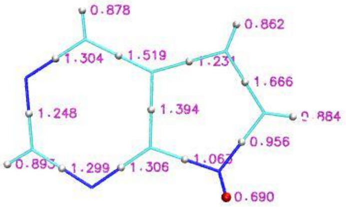

ske68

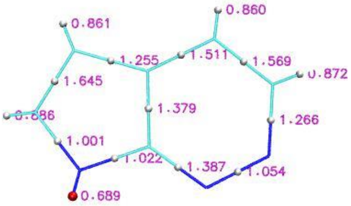

ske69

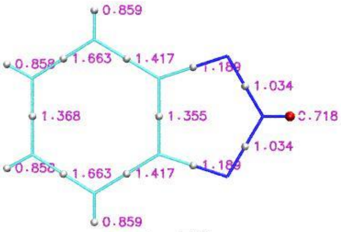

ske70

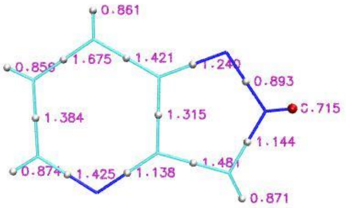

ske71

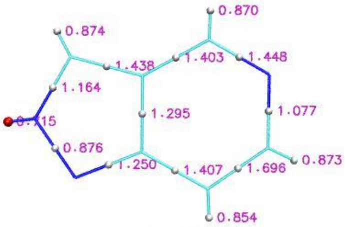

ske72

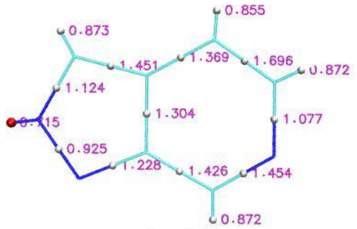

ske73

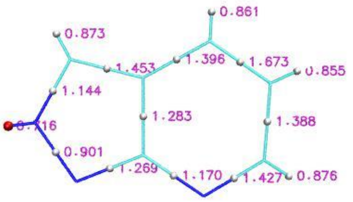

ske74

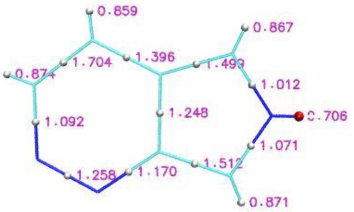

ske75

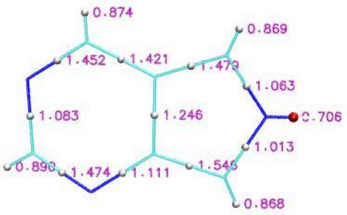

ske76

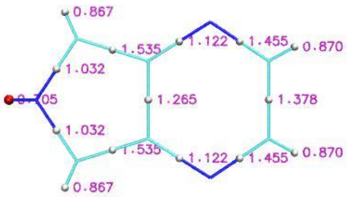

ske77

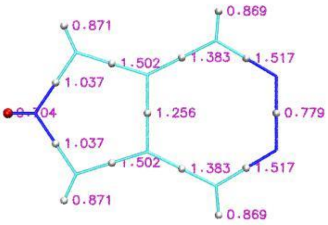

ske78

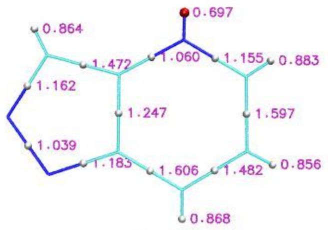

ske79

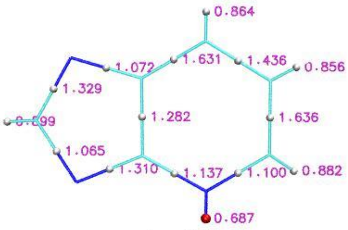

ske80

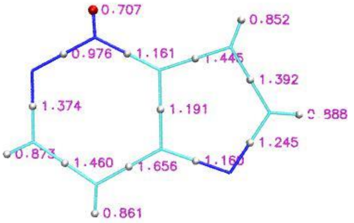

ske81

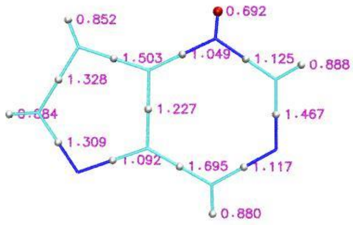

ske82

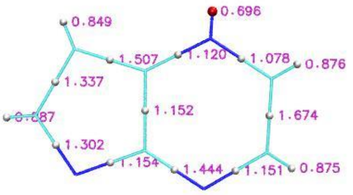

ske83

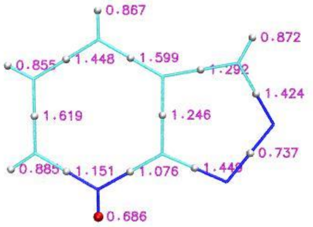

ske84

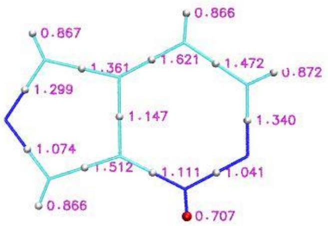

ske85

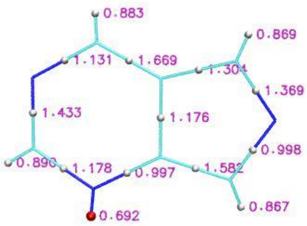

ske86

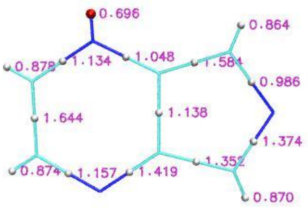

ske87

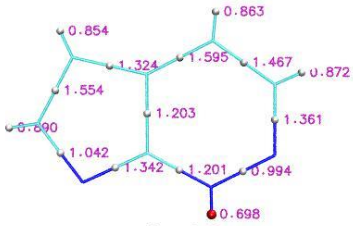

ske88

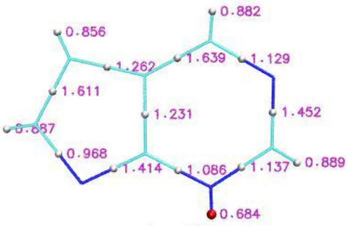

ske89

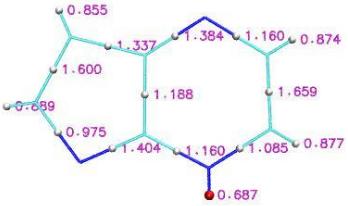

ske90

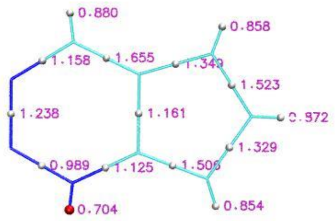

ske91

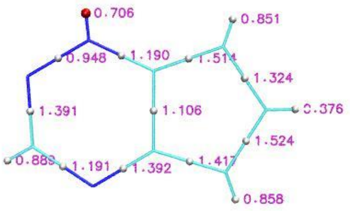

ske92

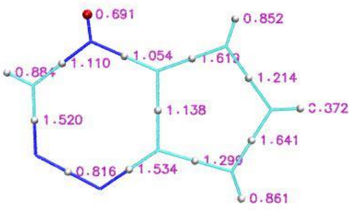

ske93

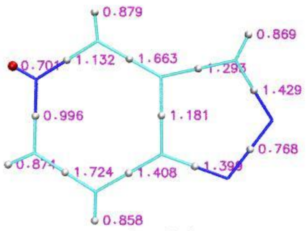

ske94

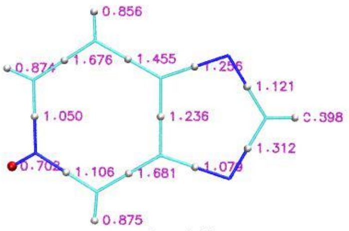

ske95

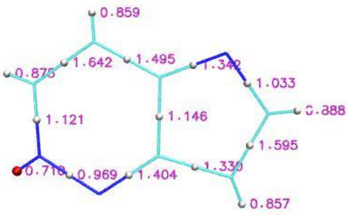

ske96

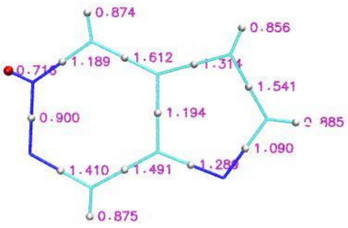

ske97

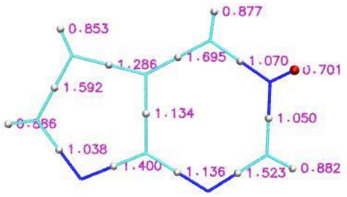

ske98

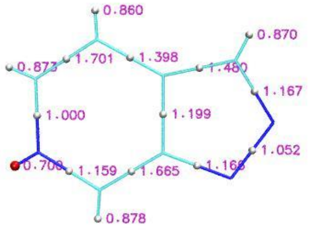

ske99

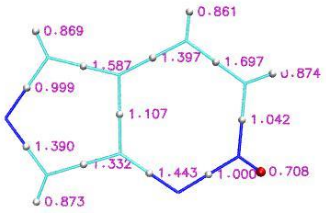

ske100

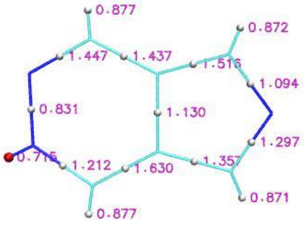

ske101

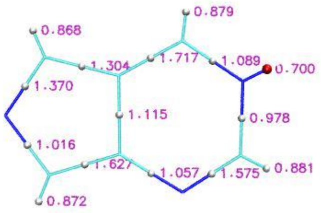

ske102

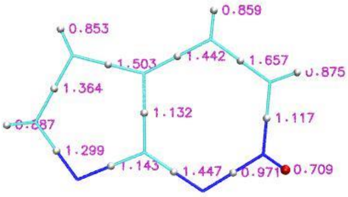

ske103

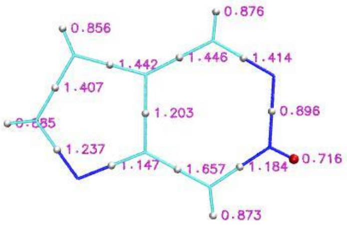

ske104

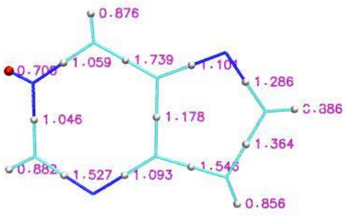

ske105

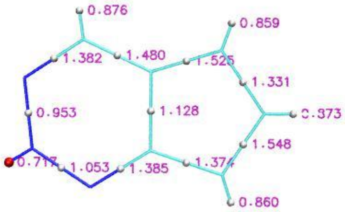

ske106

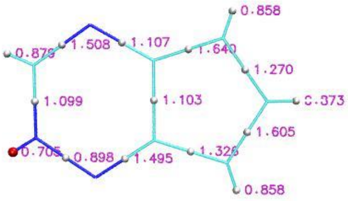

ske107

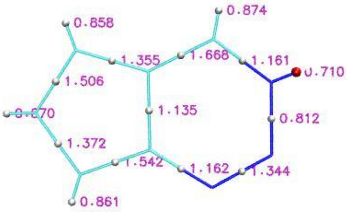

ske108

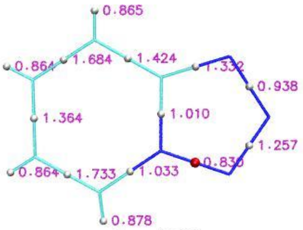

ske109

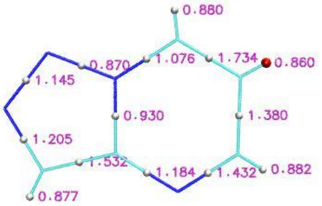

ske110

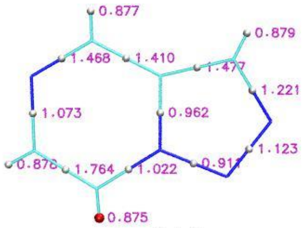

ske111

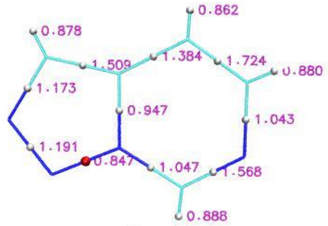

ske112

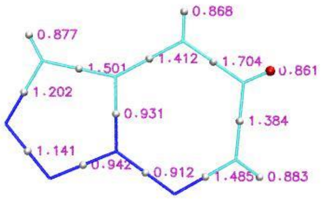

ske113

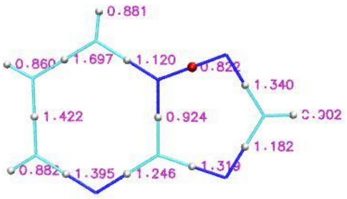

ske114

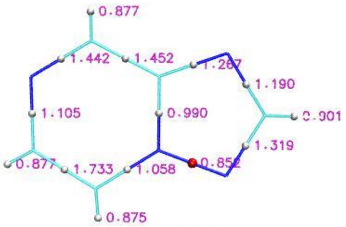

ske115

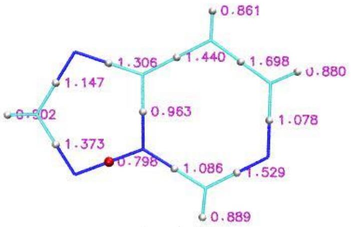

ske116

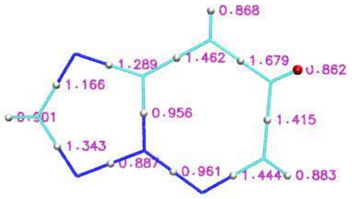

ske117

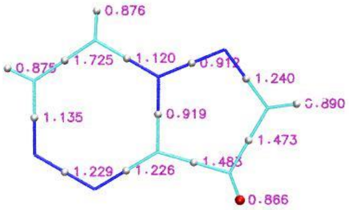

ske118

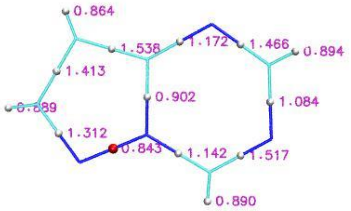

ske119

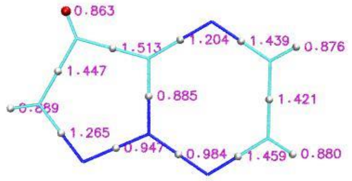

ske120

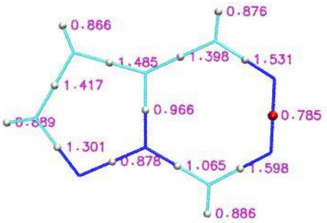

ske121

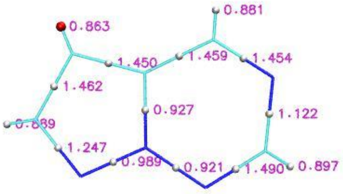

ske122

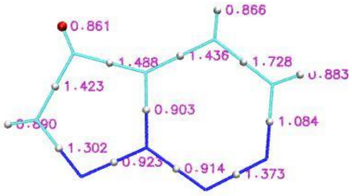

ske123

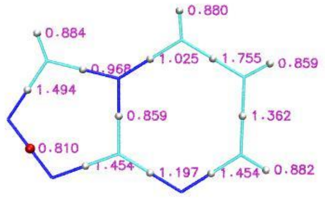

ske124

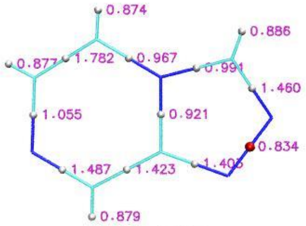

ske125

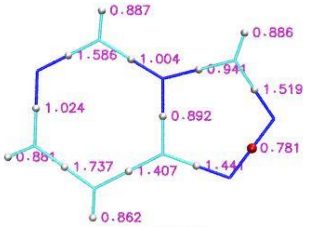

ske126

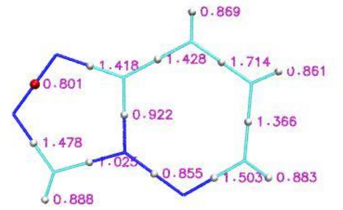

ske127

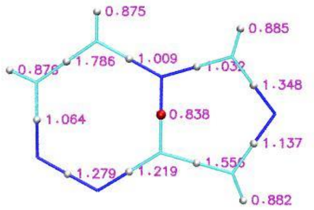

ske128

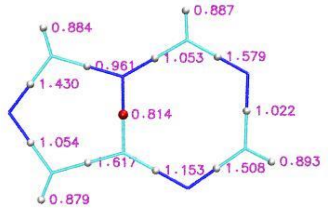

ske129

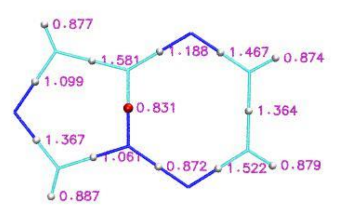

ske130

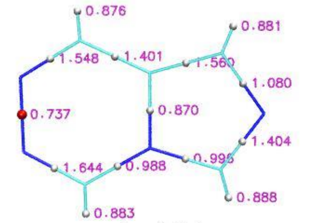

ske131

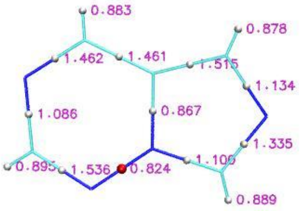

ske132

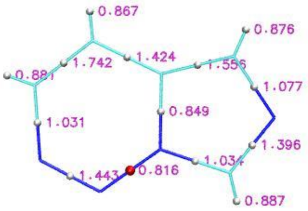

ske133

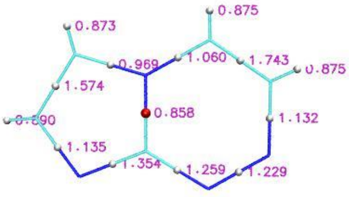

ske134

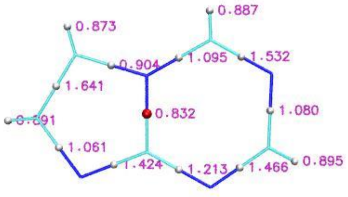

ske135

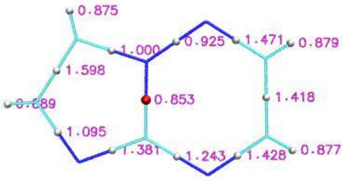

ske136

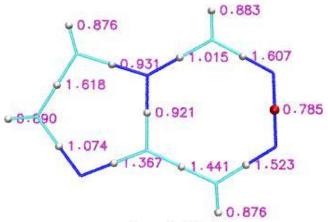

ske137

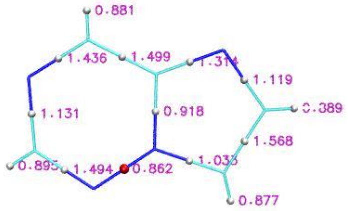

ske138

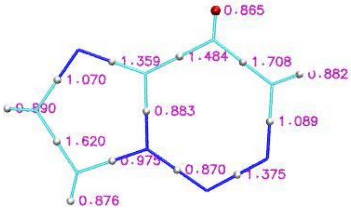

ske139

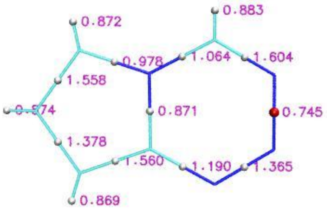

ske140

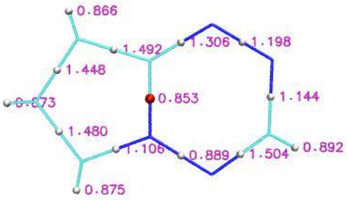

ske141

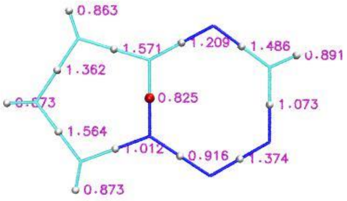

ske142

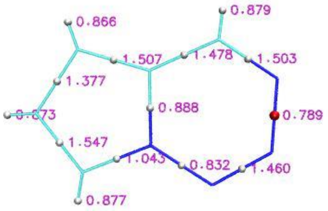

ske143

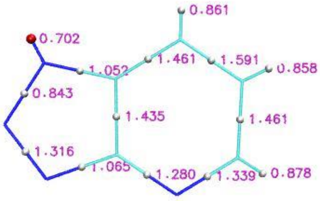

ske144

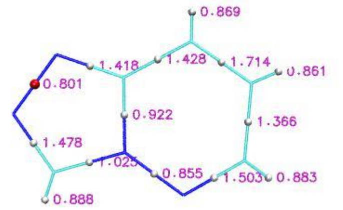

ske127

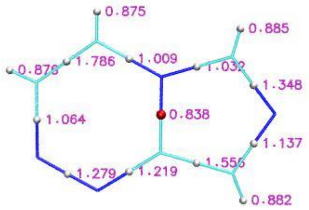

ske128

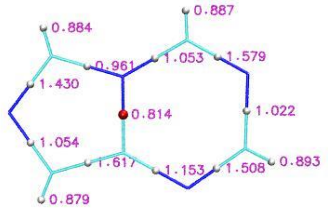

ske129

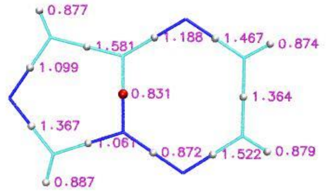

ske130

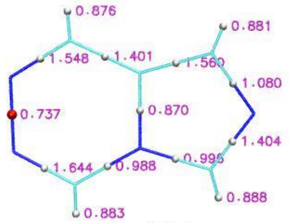

ske131

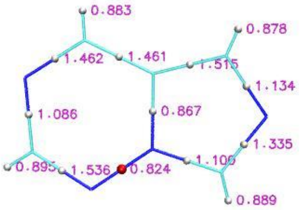

ske132

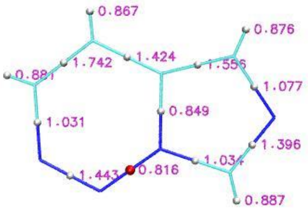

ske133

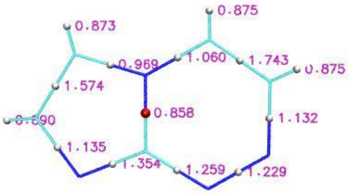

ske134

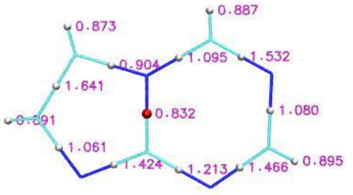

ske135

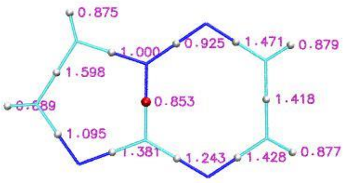

ske136

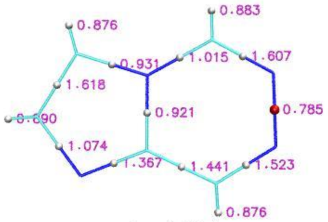

ske137

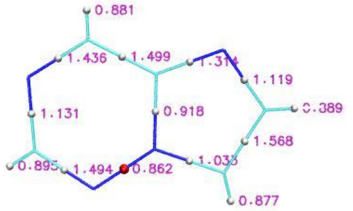

ske138

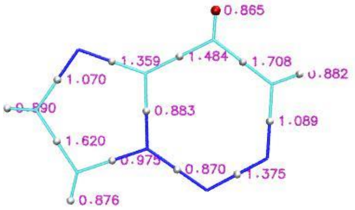

ske139

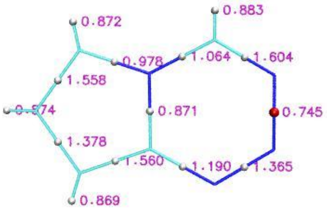

ske140

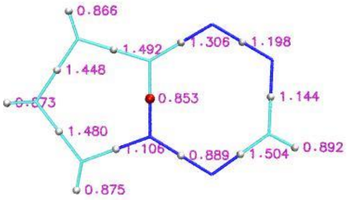

ske141

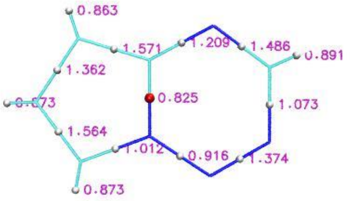

ske142

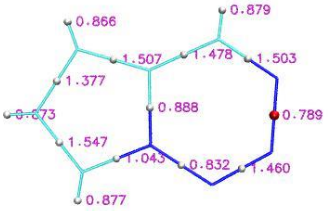

ske143

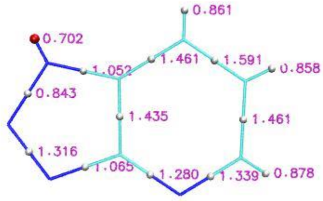

ske144

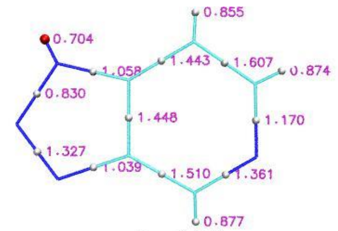

ske145

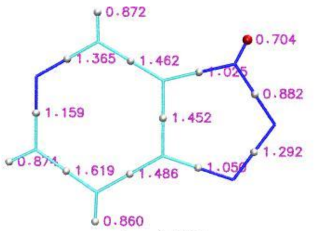

ske146

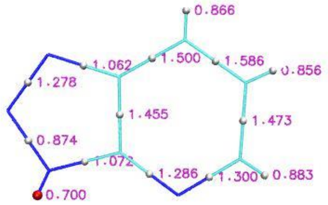

ske147

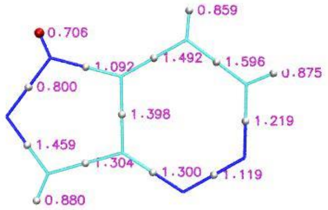

ske148

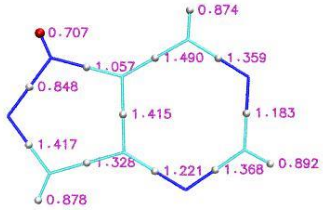

ske149

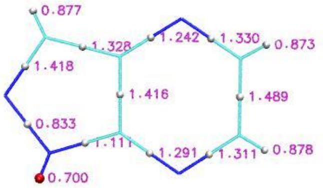

ske150

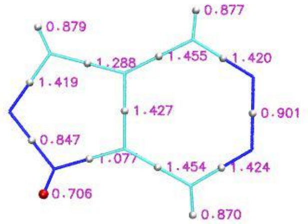

ske151

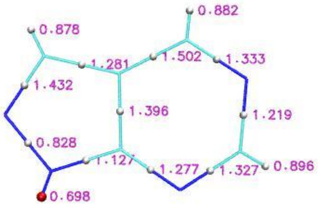

ske152

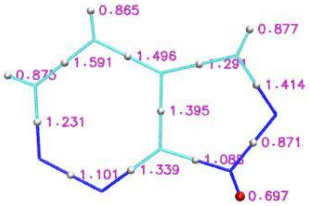

ske153

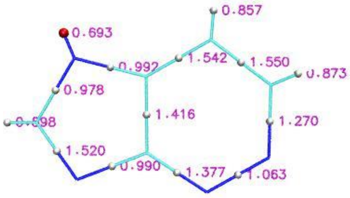

ske154

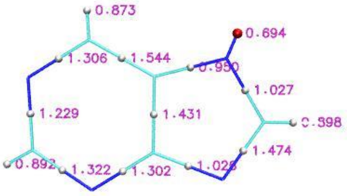

ske155

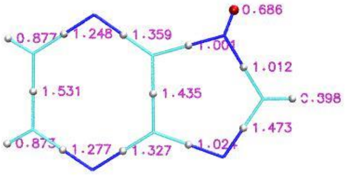

ske156

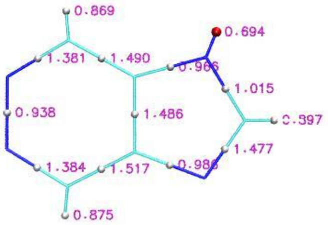

ske157

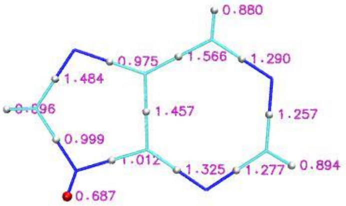

ske158

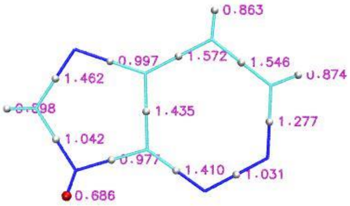

ske159

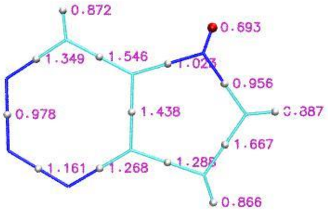

ske160

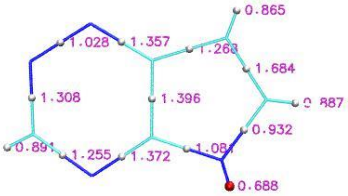

ske161

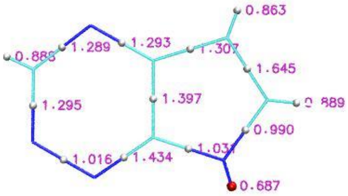

ske162

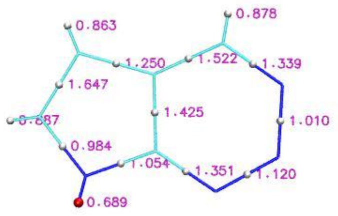

ske163

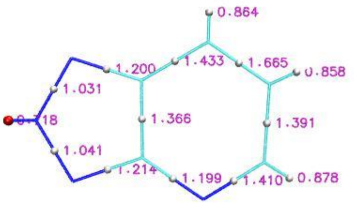

ske164

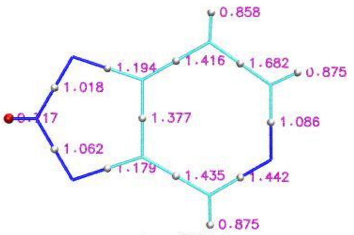

ske165

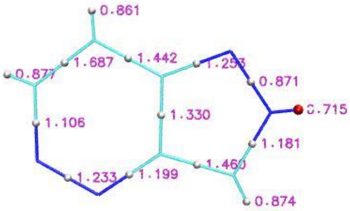

ske166

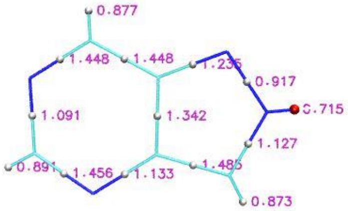

ske167

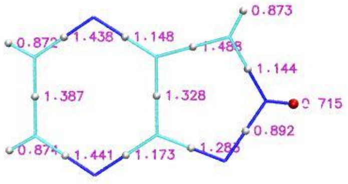

ske168

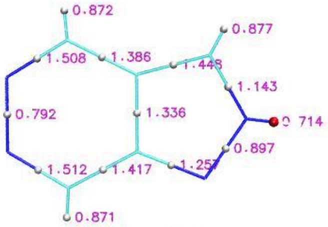

ske169

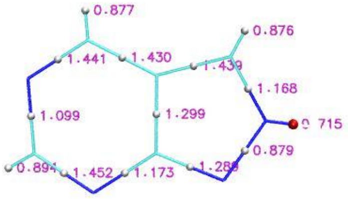

ske170

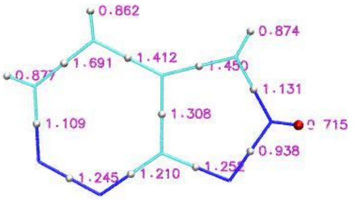

ske171

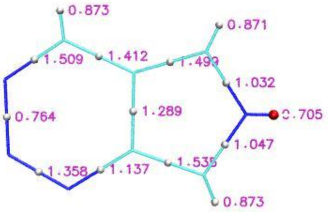

ske172

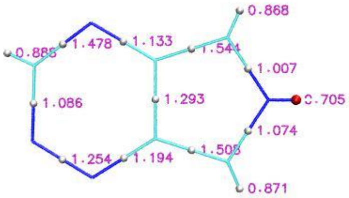

ske173

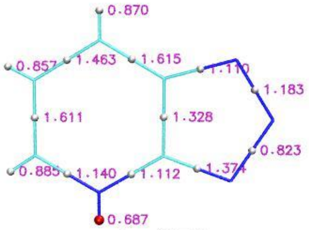

ske174

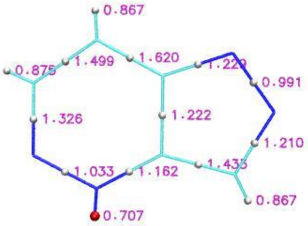

ske175

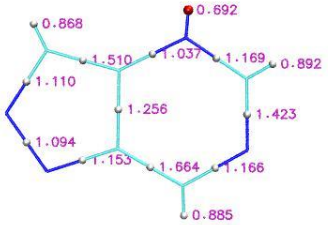

ske176

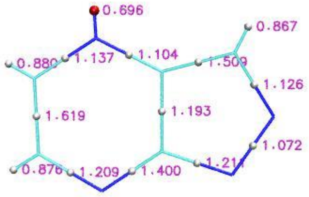

ske177

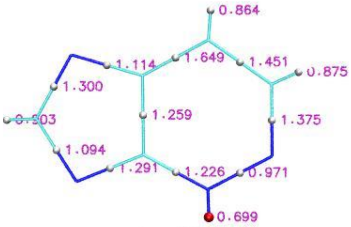

ske178

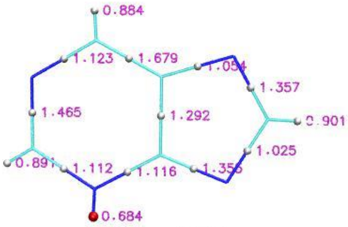

ske179

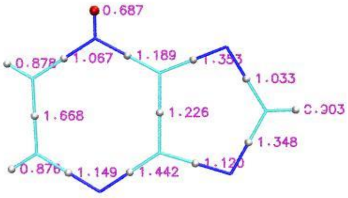

ske180

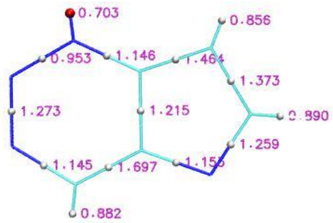

ske181

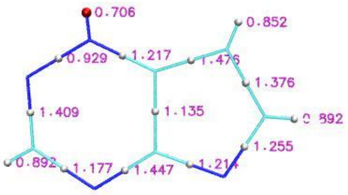

ske182

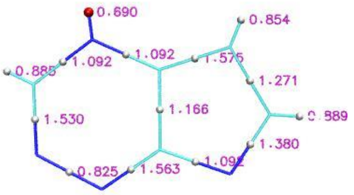

ske183

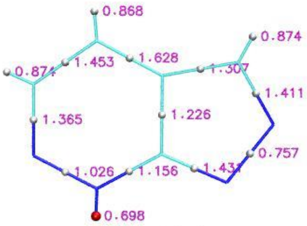

ske184

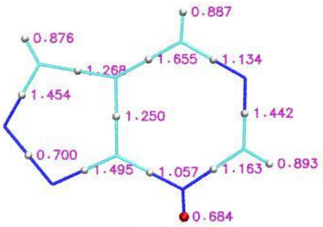

ske185

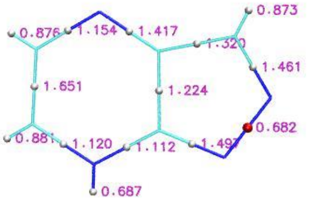

ske186

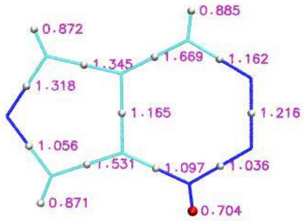

ske187

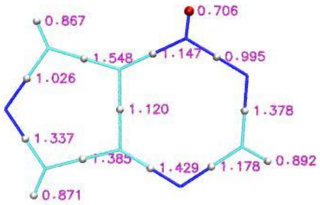

ske188

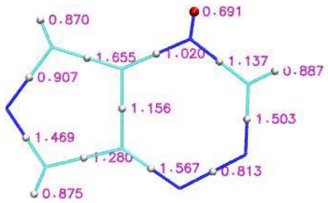

ske189

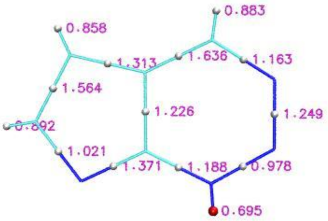

ske190

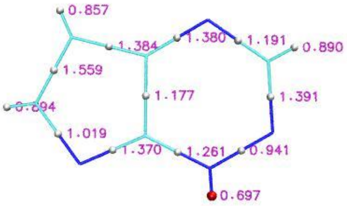

ske191

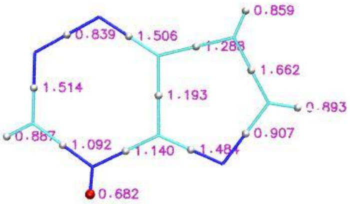

ske192

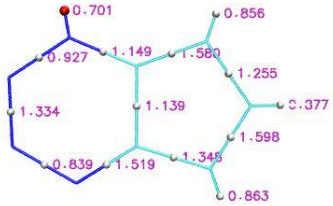

ske193

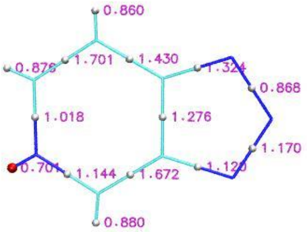

ske194

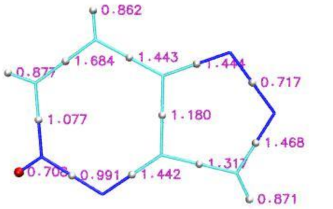

ske195

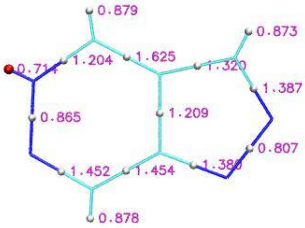

ske196

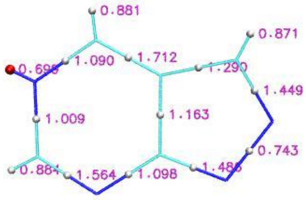

ske197

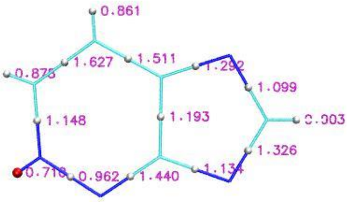

ske198

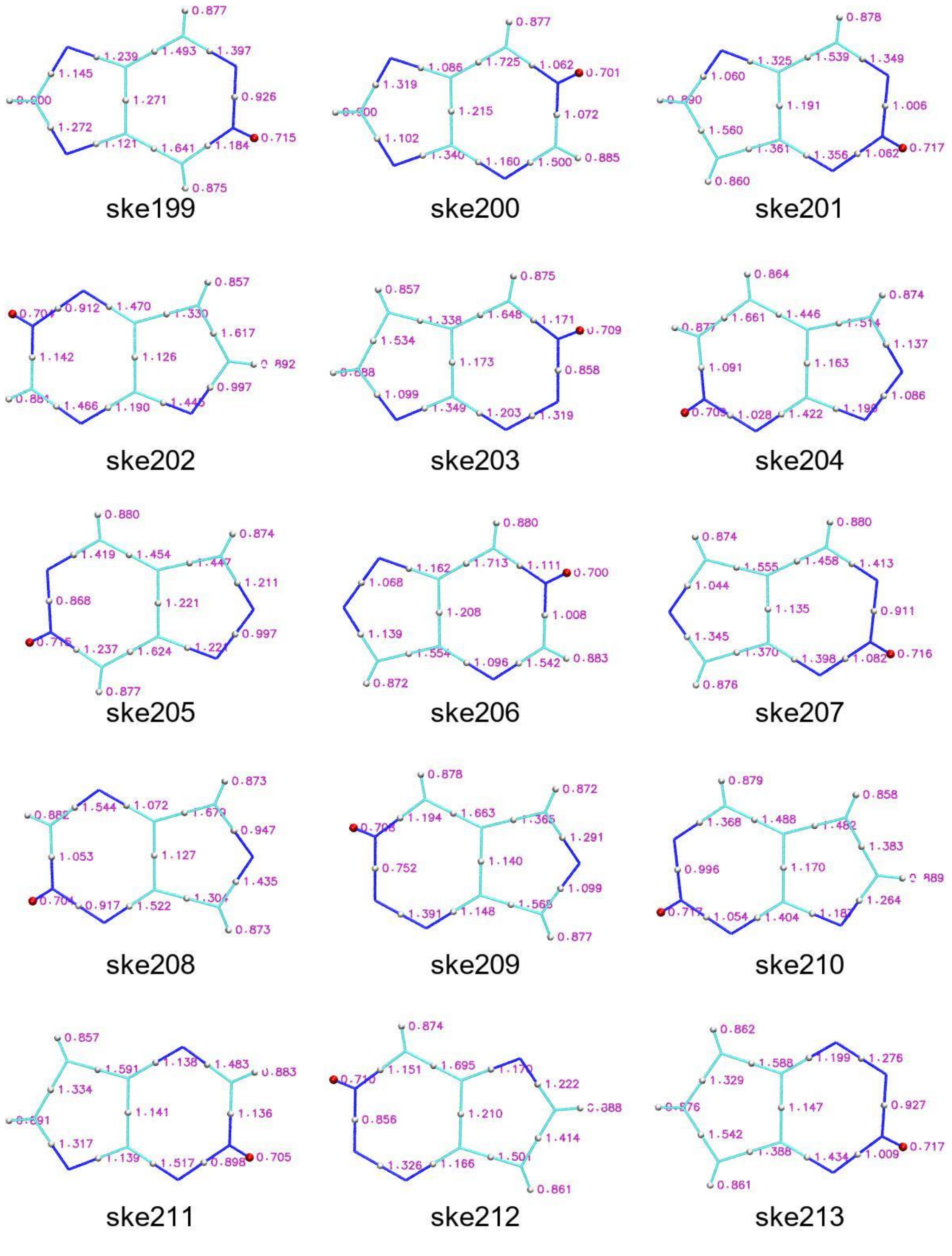

Figure S4 The LBO visualization of FR213, where red dots represent the bonds with LBO_{min}, and yellow dots represent the bonds with the $LBO_{min\text{-}noH}$.

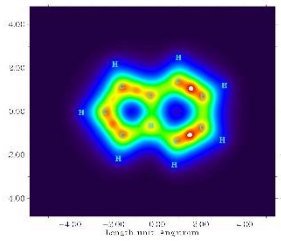
ske1
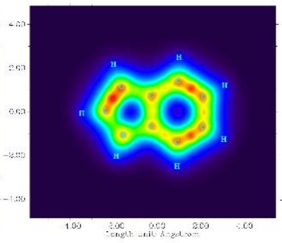
ske2
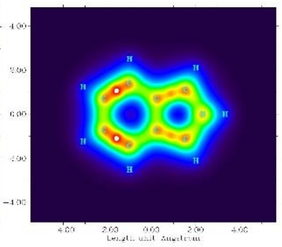
ske3
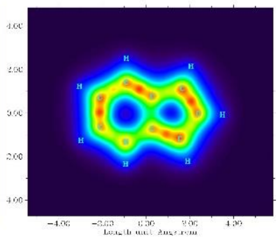
ske4
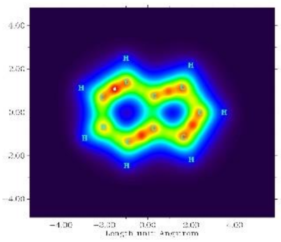
ske5
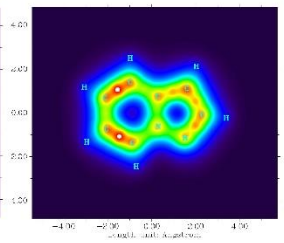
ske6
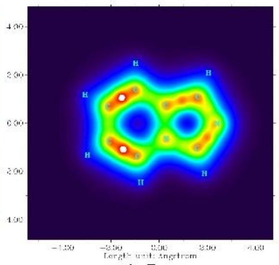
ske7
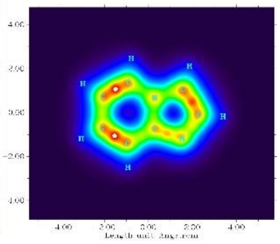
ske8
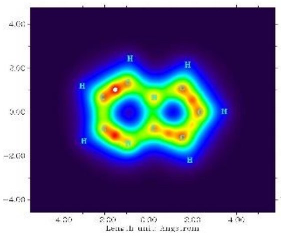
ske9
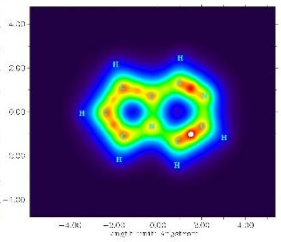
ske10
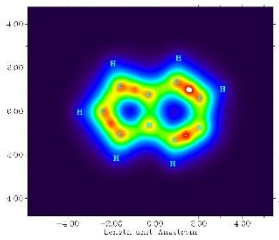
ske11
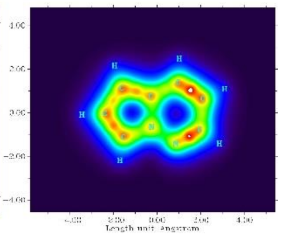
ske12
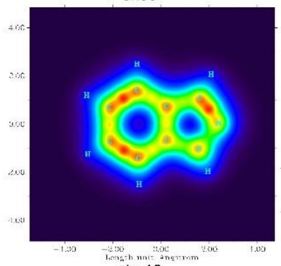
ske13
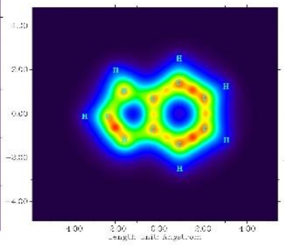
ske14
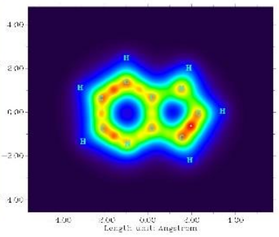
ske15
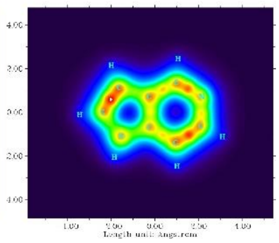
ske16
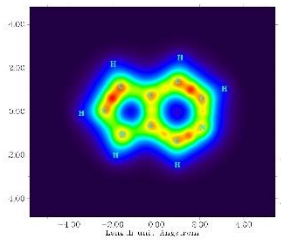
ske17
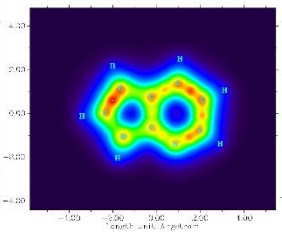
ske18
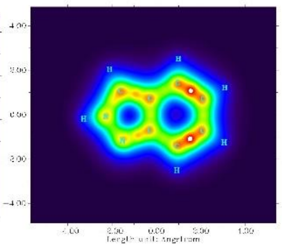
ske19
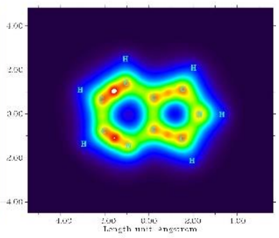
ske20
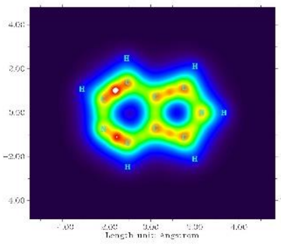
ske21
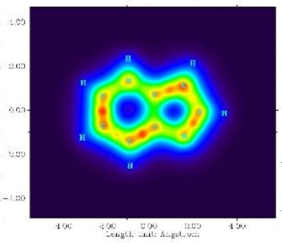
ske22
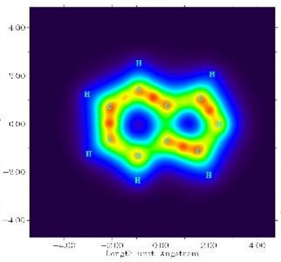
ske23
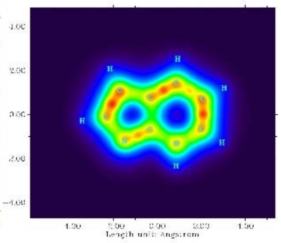
ske24

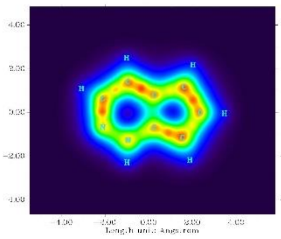

ske25

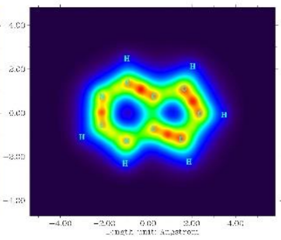

ske26

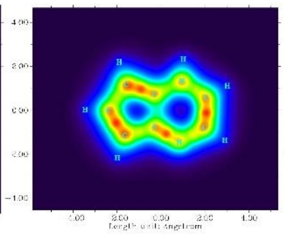

ske27

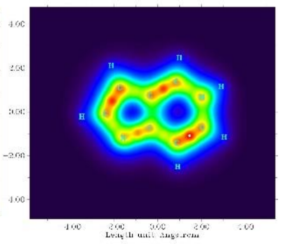

ske28

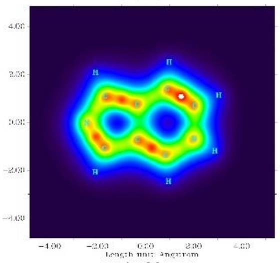

ske29

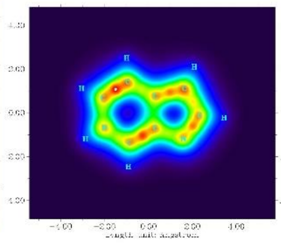

ske30

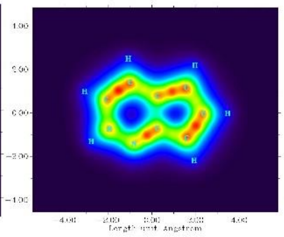

ske31

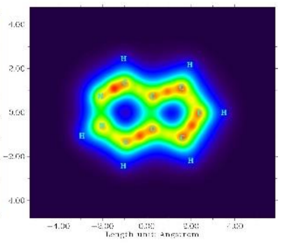

ske32

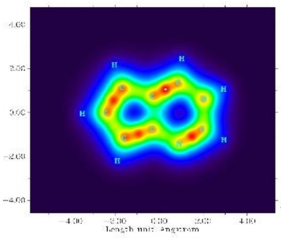

ske33

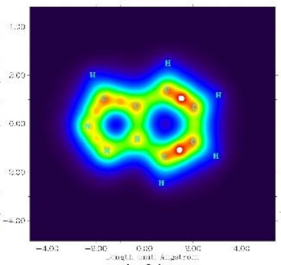

ske34

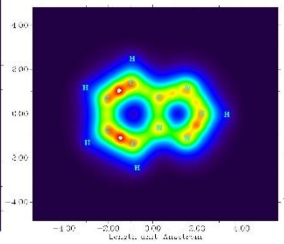

ske35

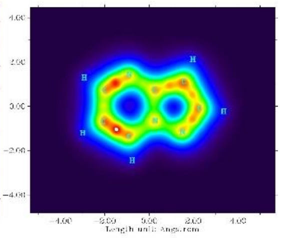

ske36

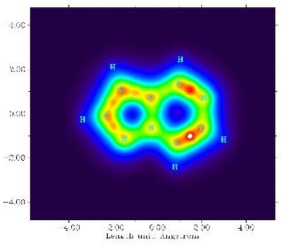

ske37

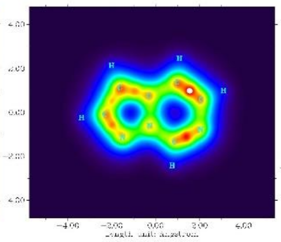

ske38

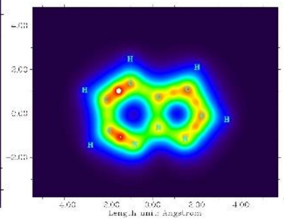

ske39

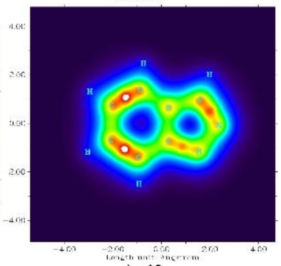

ske40

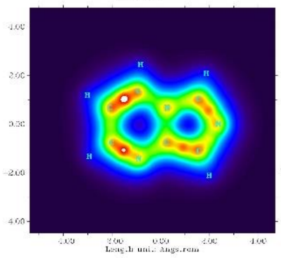

ske41

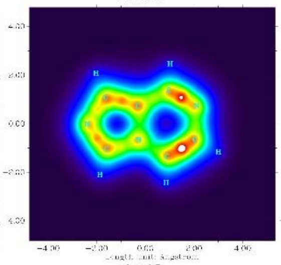

ske42

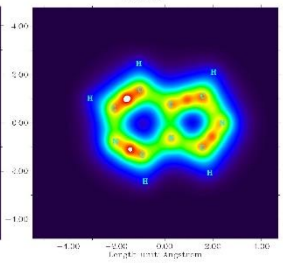

ske43

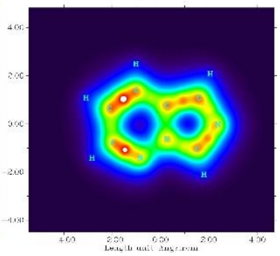

ske44

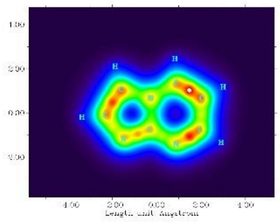

ske45

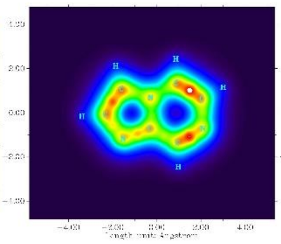

ske46

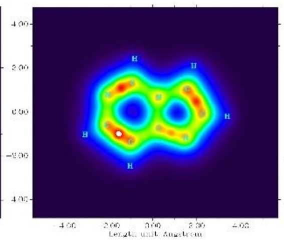

ske47

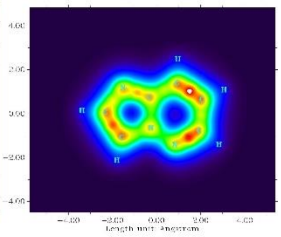

ske48

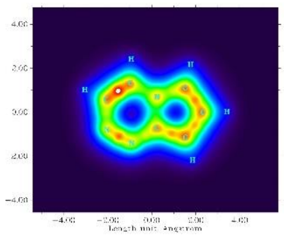

ske49

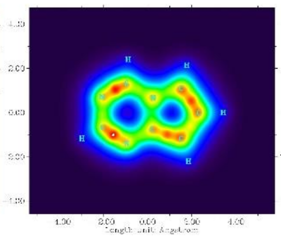

ske50

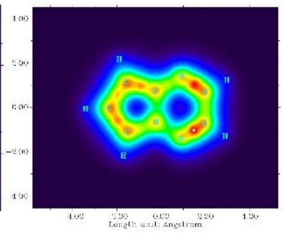

ske51

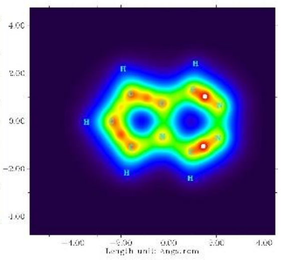

ske52

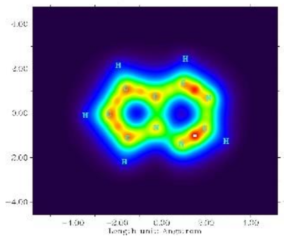

ske53

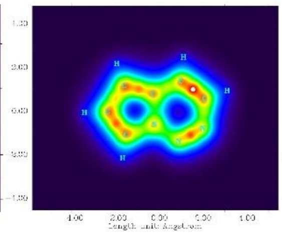

ske54

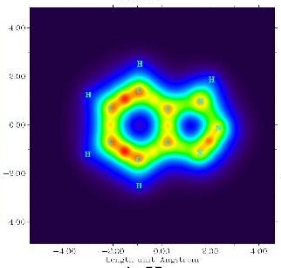

ske55

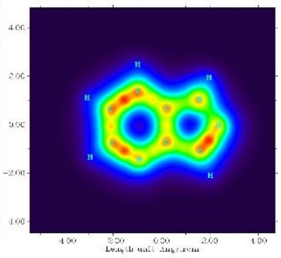

ske56

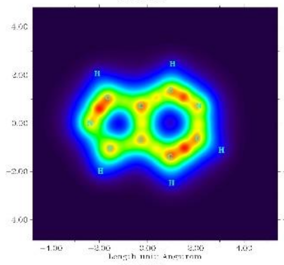

ske57

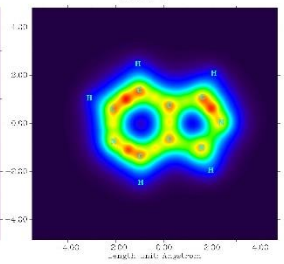

ske58

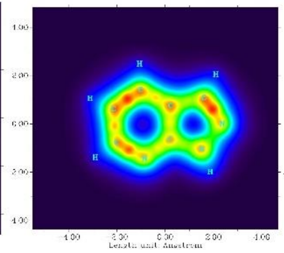

ske59

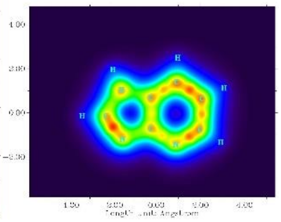

ske60

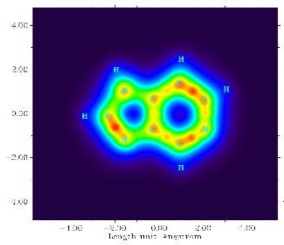

ske61

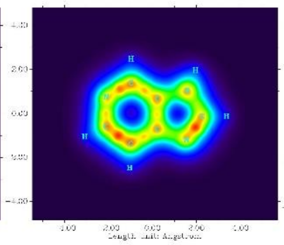

ske62

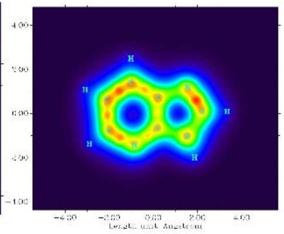

ske63

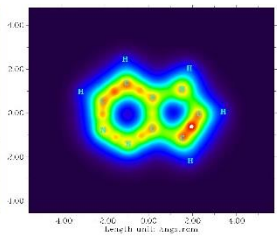

ske64

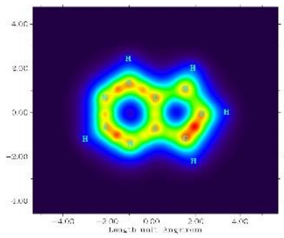

ske65

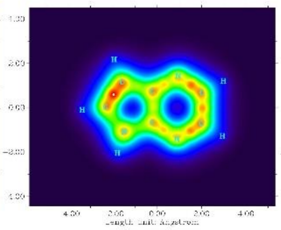

ske66

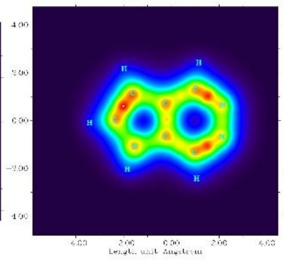

ske67

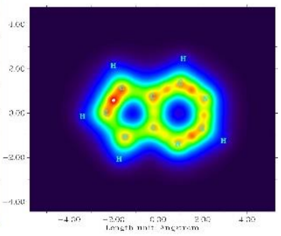

ske68

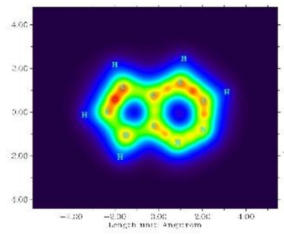

ske69

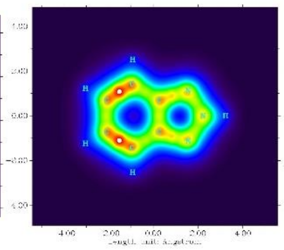

ske70

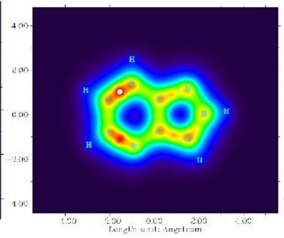

ske71

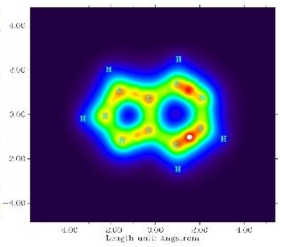

ske72

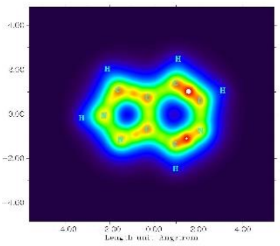
ske73
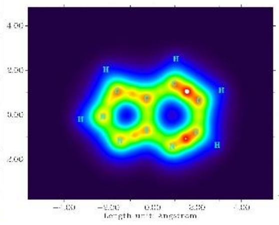
ske74
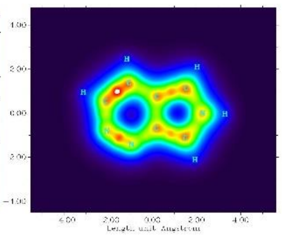
ske75
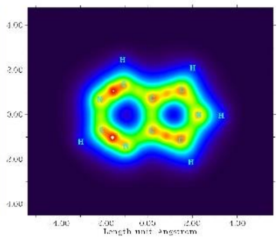
ske76
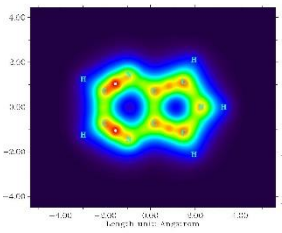
ske77
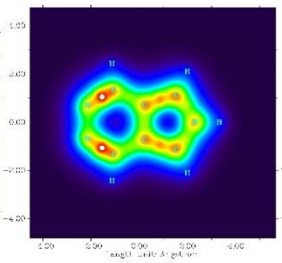
ske78
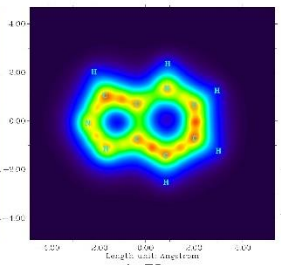
ske79
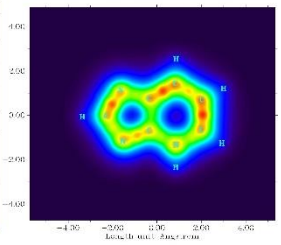
ske80
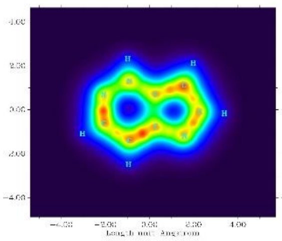
ske81
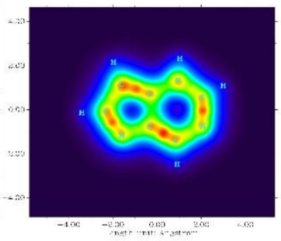
ske82
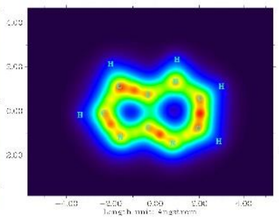
ske83
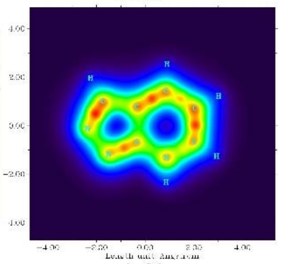
ske84
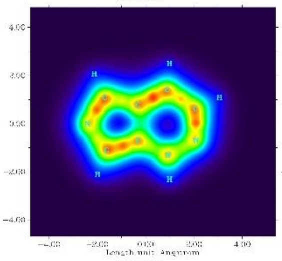
ske85
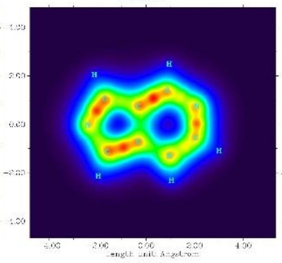
ske86
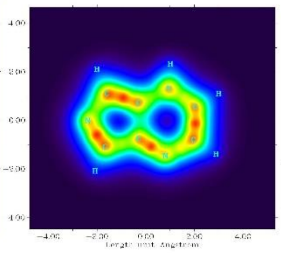
ske87
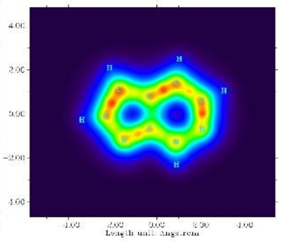
ske88
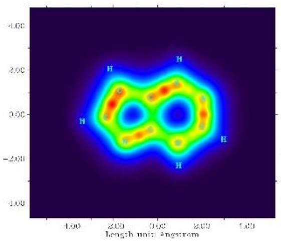
ske89
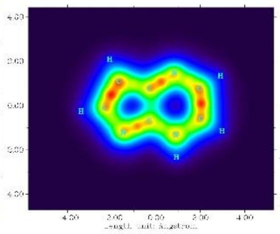
ske90
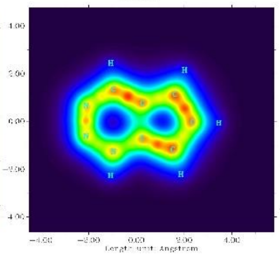
ske91
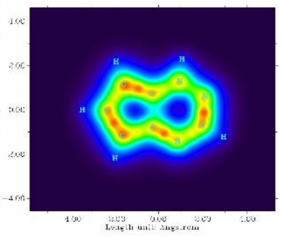
ske92
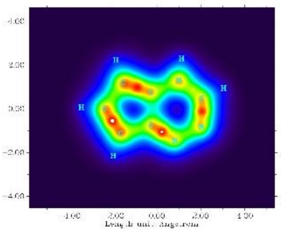
ske93
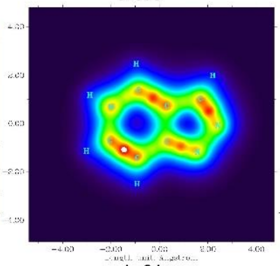
ske94
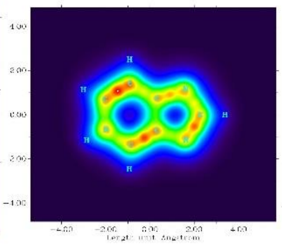
ske95
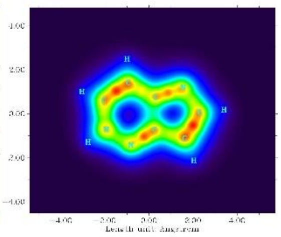
ske96

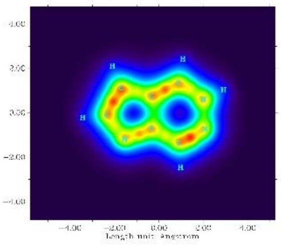
ske97
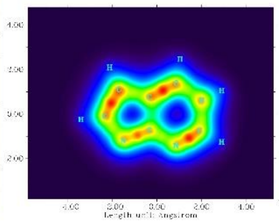
ske98
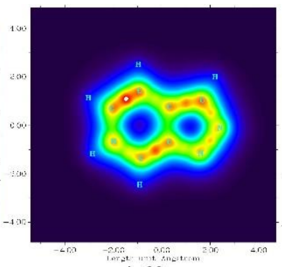
ske99
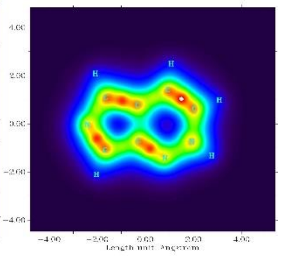
ske100
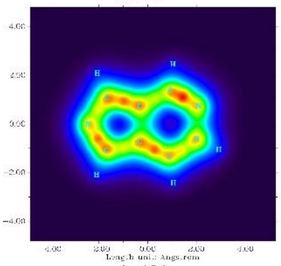
ske101
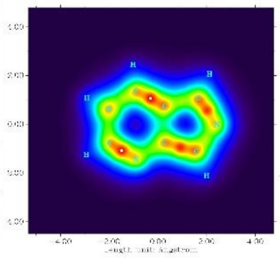
ske102
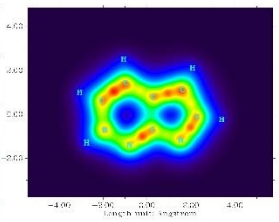
ske103
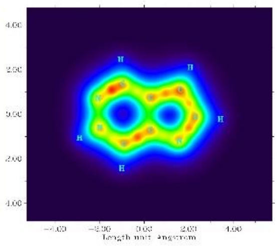
ske104
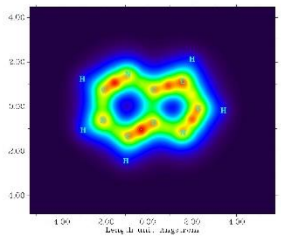
ske105
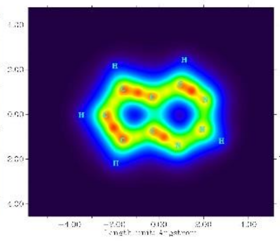
ske106
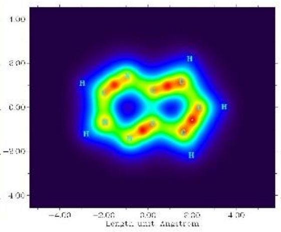
ske107
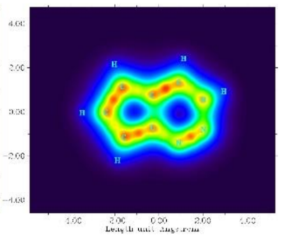
ske108
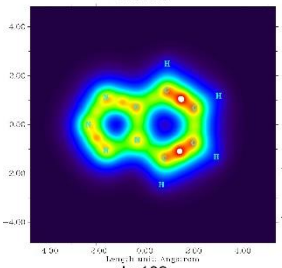
ske109
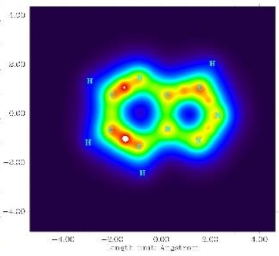
ske110
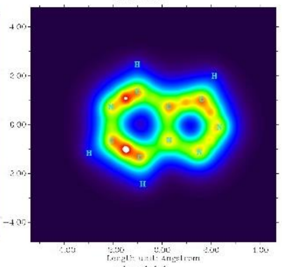
ske111
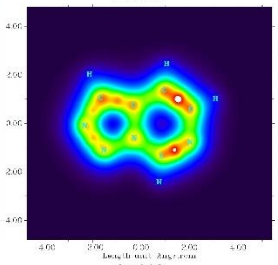
ske112
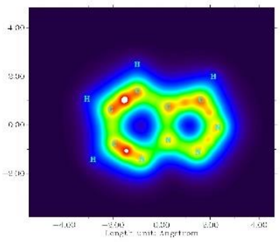
ske113
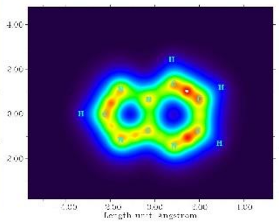
ske114
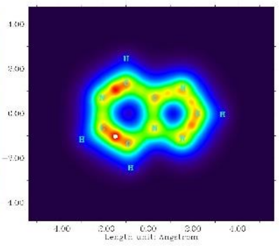
ske115
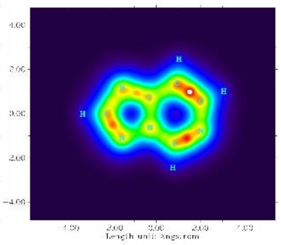
ske116
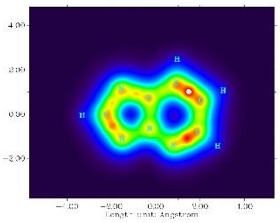
ske117
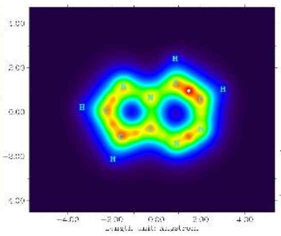
ske118
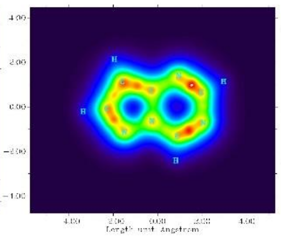
ske119
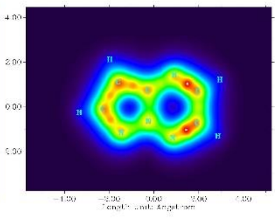
ske120

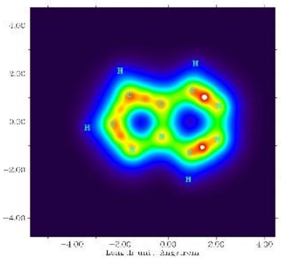

ske121

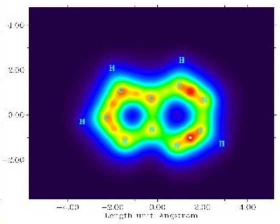

ske122

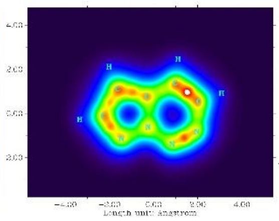

ske123

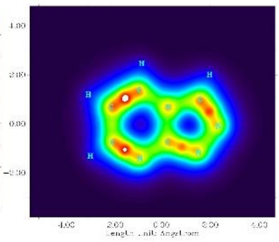

ske124

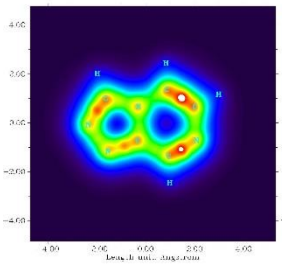

ske125

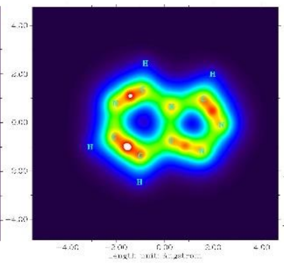

ske126

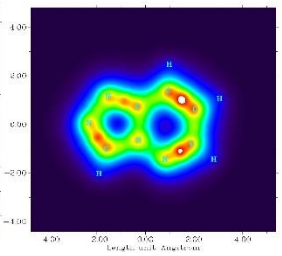

ske127

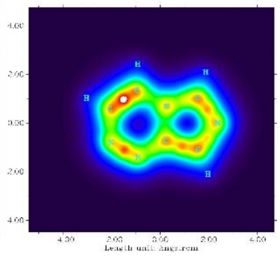

ske128

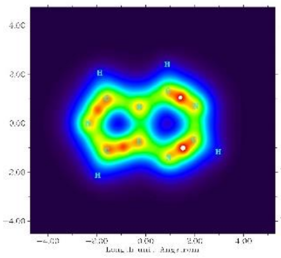

ske129

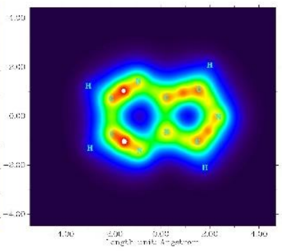

ske130

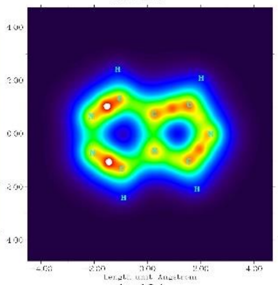

ske131

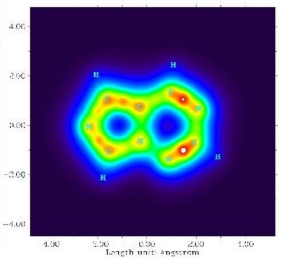

ske132

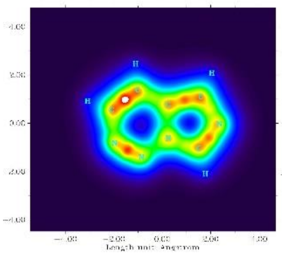

ske133

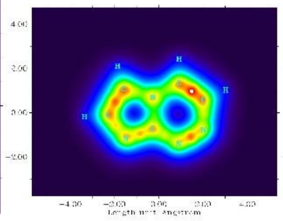

ske134

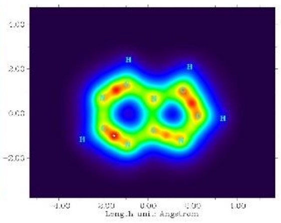

ske135

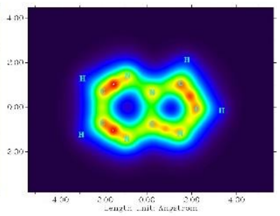

ske136

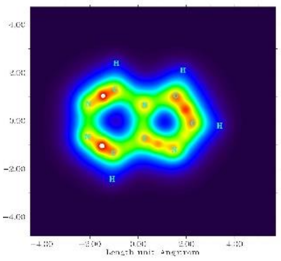

ske137

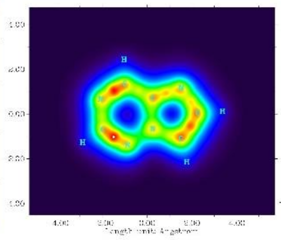

ske138

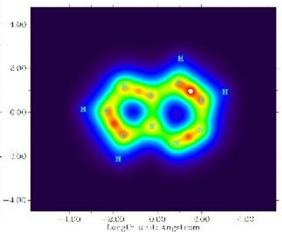

ske139

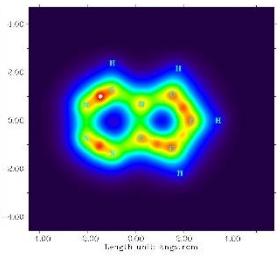

ske140

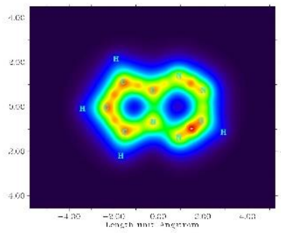

ske141

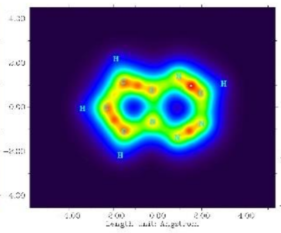

ske142

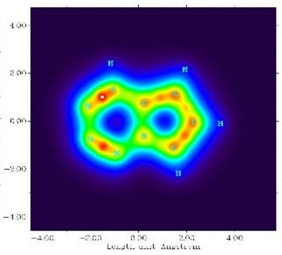

ske143

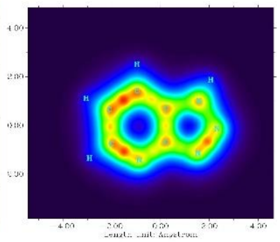

ske144

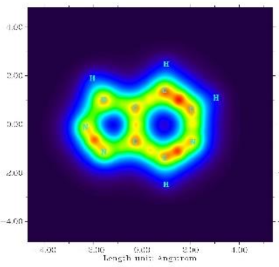

ske145

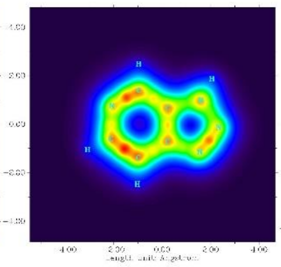

ske146

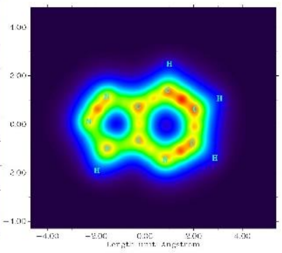

ske147

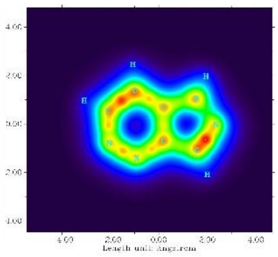

ske148

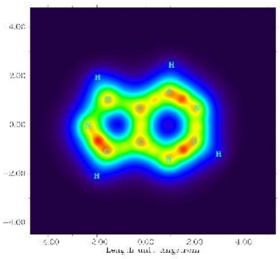

ske149

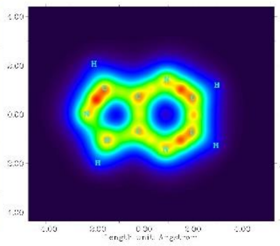

ske150

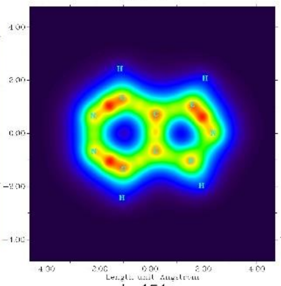

ske151

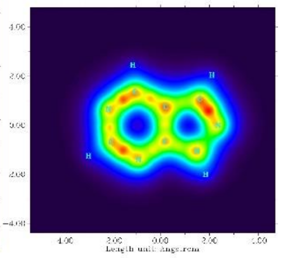

ske152

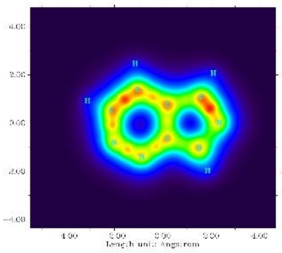

ske153

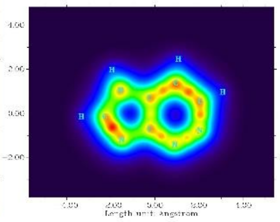

ske154

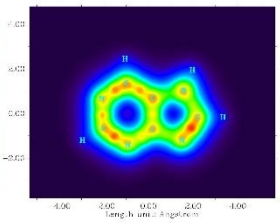

ske155

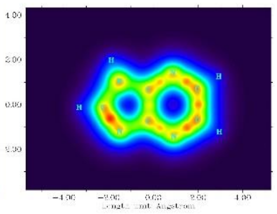

ske156

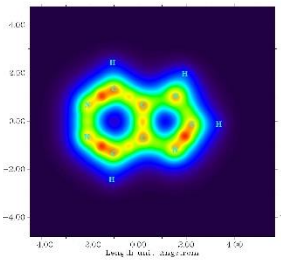

ske157

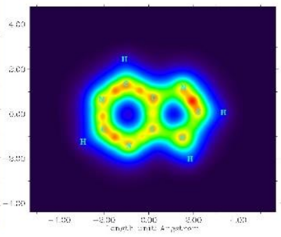

ske158

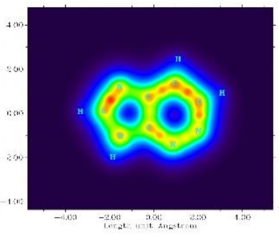

ske159

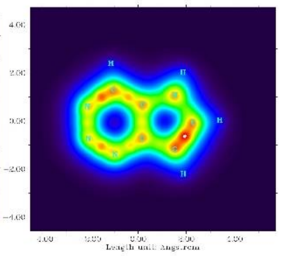

ske160

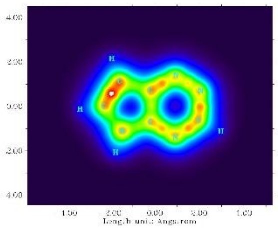

ske161

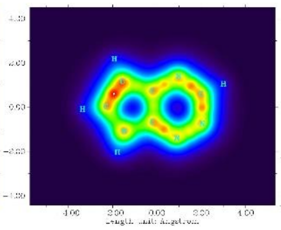

ske162

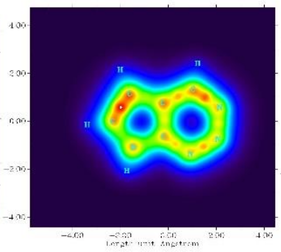

ske163

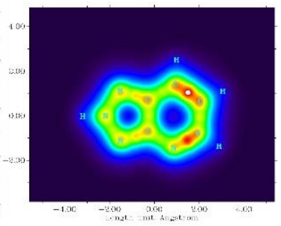

ske164

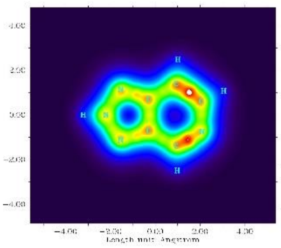

ske165

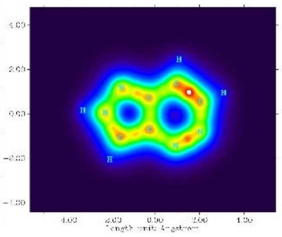

ske166

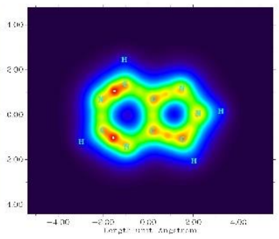

ske167

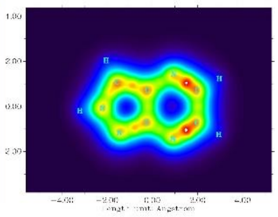

ske168

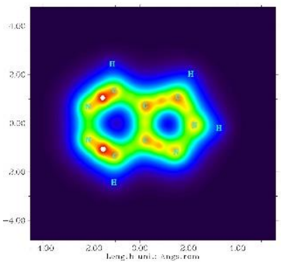
ske169

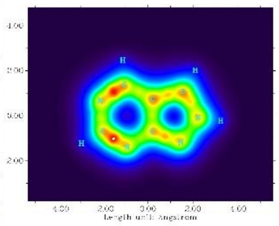
ske170

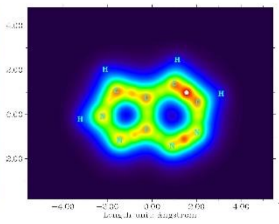
ske171

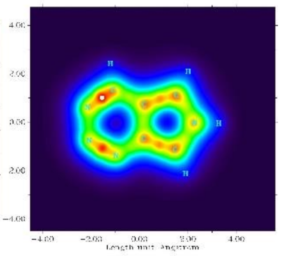
ske172

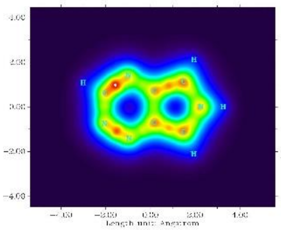
ske173

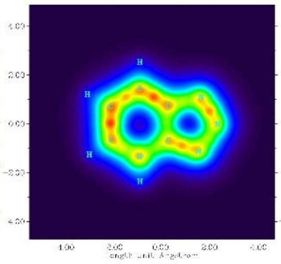
ske174

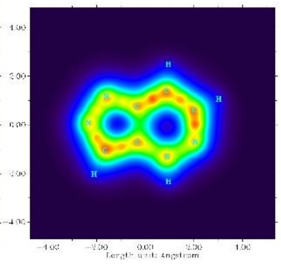
ske175

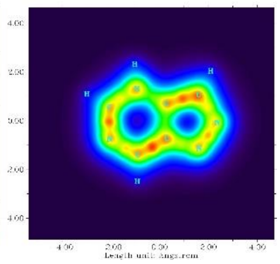
ske176

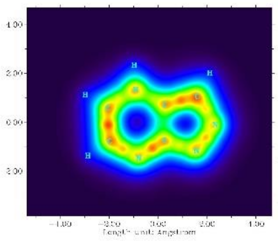
ske177

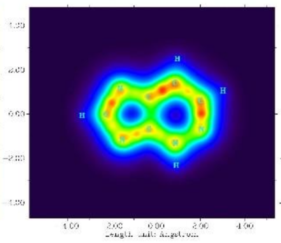
ske178

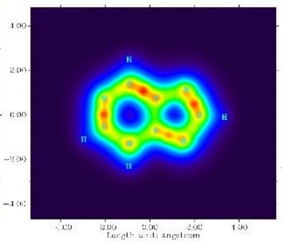
ske179

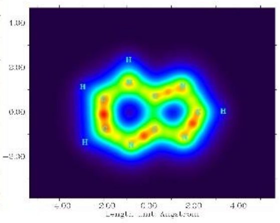
ske180

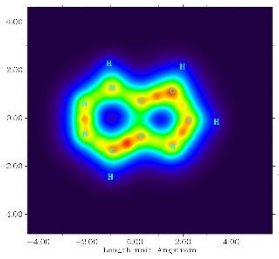
ske181

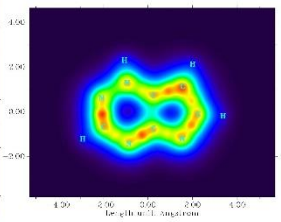
ske182

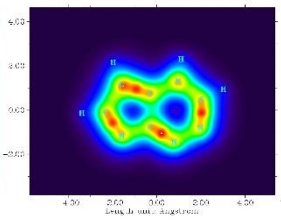
ske183

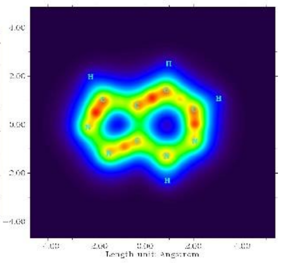
ske184

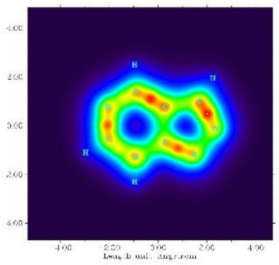
ske185

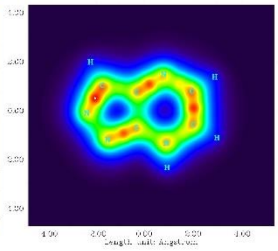
ske186

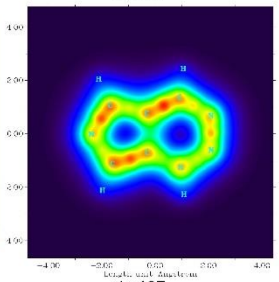
ske187

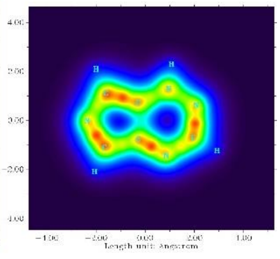
ske188

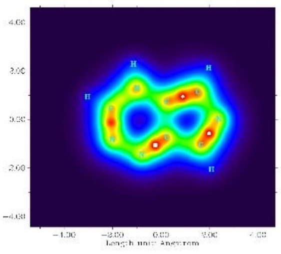
ske189

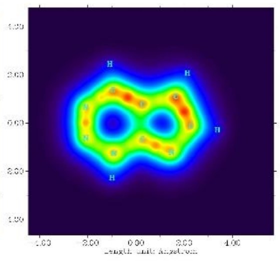
ske190

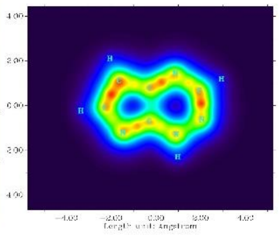
ske191

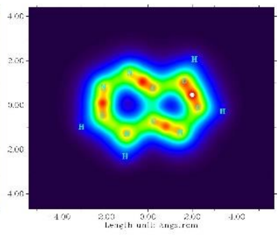
ske192

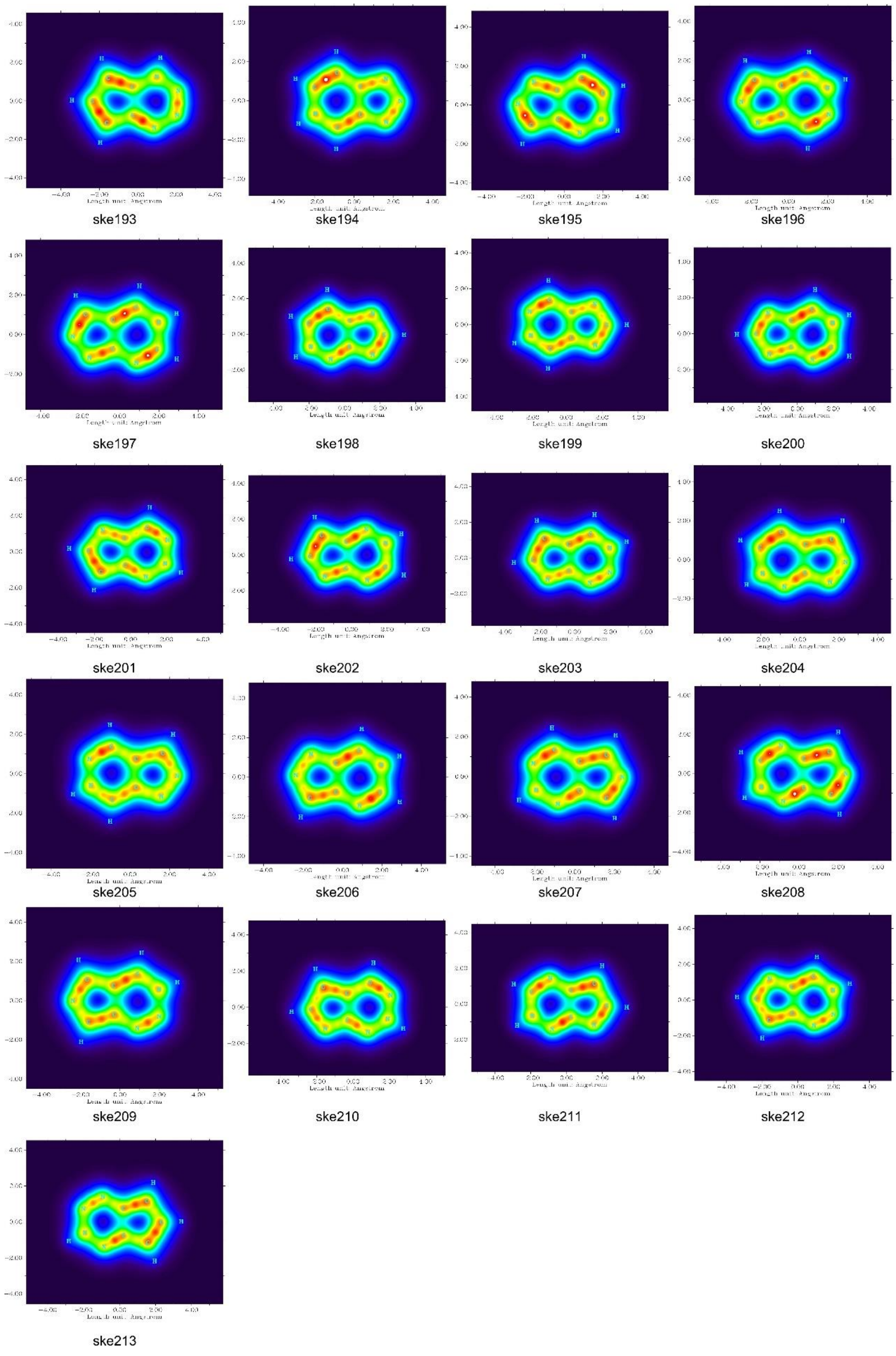

Figure S5 The LOL-π color-filled plots of FR213.

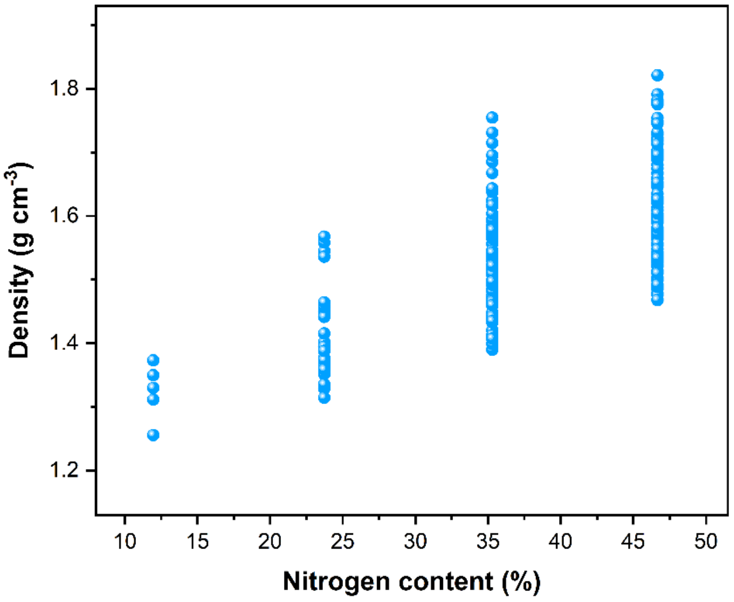

Figure S6 Distribution of densities for FR213 with different nitrogen content.

Calculation method for enthalpy of formation

The enthalpy change of producing one mole of a compound from its simple substances in their standard states is defined as the standard molar enthalpy of formation of the compound. Taking the compound $C_aH_bN_cO_d$ as an example, the corresponding general equation for its formation reaction is as follows:

$$aC + \frac{b}{2}H_2 + \frac{c}{2}N_2 + \frac{d}{2}O_2 \rightarrow C_aH_bN_cO_d$$

By definition, the enthalpy of formation is the reaction enthalpy of the above equation. The enthalpy of reaction is determined by calculating the difference between the enthalpy of the products and the enthalpy of the reactants. To calculate the enthalpy of each species involved in the reaction, quantum chemistry computational programs such as Gaussian or ORCA can be used. Carbon is a solid at room temperature, but the enthalpy value obtained from quantum chemistry calculation corresponds to its formation in the gaseous state. Subtracting the sublimation enthalpy ($H_{sub}(C)$) from

the enthalpy value ($H(C)$) obtained from quantum chemistry calculation is necessary to determine the enthalpy of carbon in its stable phase (solid).

In summary, the formula for calculating the enthalpy of formation using the definition method is as follows:

$$\Delta_f H_m^\theta = \Delta_r H_m^\theta = H(C_a H_b N_c O_d) - a[H(C) - H_{sub}(C)] - \frac{b}{2}H(H_2) - \frac{c}{2}H(N_2) - \frac{d}{2}H(O_2)$$

All the enthalpy values provided above are enthalpies at 298 K. The enthalpy represented by $H(C_a H_b N_c O_d)$ corresponds to the enthalpy of a single molecule. Therefore, the final result denotes the gas-phase enthalpy of formation of the compound at 298 K.

For the FR213 system, the enthalpy value is obtained by adding the enthalpy correction (H_{cor}), derived from geometry optimization and vibrational analysis performed at the M062X/def2TZVP level, to the electronic energy obtained from high-precision single-point calculations at the PWPB95-D3/def2-QZVPP level. The zero-point energy (ZPE) in H_{cor} is corrected, with a correction factor of 0.9710 (from Truhlar). The geometry optimization and vibrational analysis were conducted using Gaussian 09, while the high-precision single-point calculations were carried out using ORCA 5.02 (Neese, F. The ORCA Program System. *WIREs Comput Mol Sci* **2012**, *2* (1), 73–78. https://doi.org/10.1002/wcms.81.).
